# Conserved age‐related increases in hippocampal PDE11A4 cause unexpected proteinopathies and cognitive decline of social associative memories

**DOI:** 10.1111/acel.13687

**Published:** 2022-09-08

**Authors:** Katy Pilarzyk, Latarsha Porcher, William R. Capell, Steven D. Burbano, Jeff Davis, Janet L. Fisher, Nicole Gorny, Siena Petrolle, Michy P. Kelly

**Affiliations:** ^1^ Department of Pharmacology, Physiology & Neuroscience University of South Carolina School of Medicine Columbia South Carolina USA; ^2^ Instrument Resource Facility University of South Carolina School of Medicine Columbia South Carolina USA; ^3^ Department of Anatomy & Neurobiology University of Maryland School of Medicine Baltimore Maryland USA; ^4^ Center for Research on Aging University of Maryland School of Medicine Baltimore Maryland USA

**Keywords:** age‐related cognitive impairment, Alzheimer's disease, COS‐1, HEK293T, hippocampus, HT‐22, learning, memory, phosphodiesterase, proteopathy, STFP, TBI

## Abstract

In humans, associative memories are more susceptible to age‐related cognitive decline (ARCD) than are recognition memories. Reduced cAMP/cGMP signaling in the hippocampus may contribute to ARCD. Here, we found that both aging and traumatic brain injury‐associated dementia increased the expression of the cAMP/cGMP‐degrading enzyme phosphodiesterase 11A (PDE11A) in the human hippocampus. Further, age‐related increases in hippocampal PDE11A4 mRNA and protein were conserved in mice, as was the increased vulnerability of associative versus recognition memories to ARCD. Interestingly, mouse PDE11A4 protein in the aged ventral hippocampus (VHIPP) ectopically accumulated in the membrane fraction and filamentous structures we term “ghost axons.” These age‐related increases in expression were driven by reduced exoribonuclease‐mediated degradation of PDE11A mRNA and increased PDE11A4‐pS117/pS124, the latter of which also drove the punctate accumulation of PDE11A4. In contrast, PDE11A4‐pS162 caused dispersal. Importantly, preventing age‐related increases in PDE11 expression via genetic deletion protected mice from ARCD of short‐term and remote long‐term associative memory (aLTM) in the social transmission of food preference assay, albeit at the expense of recent aLTM. Further, mimicking age‐related overexpression of PDE11A4 in CA1 of old KO mice caused aging‐like impairments in CREB function and remote social—but not non‐social—LTMs. RNA sequencing and phosphoproteomic analyses of VHIPP identified cGMP‐PKG—as opposed to cAMP‐PKA—as well as circadian entrainment, glutamatergic/cholinergic synapses, calcium signaling, oxytocin, and retrograde endocannabinoid signaling as mechanisms by which PDE11A deletion protects against ARCD. Together, these data suggest that PDE11A4 proteinopathies acutely impair signaling in the aged brain and contribute to ARCD of social memories.

## Graphical abstract

We identify striking ectopic proteinopathies in the hippocampally‐enriched cAMP/cGMP‐degrading enzyme PDE11A4 as a conserved molecular determinant driving age‐related cognitive decline (ARCD) of associative long‐term memories. Mechanistically, these age‐related PDE11A4 proteinopathies are driven by increased translation and altered trafficking triggered by phosphorylation of serines S117 and S124 in the PDE11A4 regulatory domain. RNA and phosphoproteomic analyses identified cGMP‐PKG (as opposed to cAMP‐PKA), circadian entrainment, glutamate/choline, calcium, oxytocin, and retrograde endocannabinoid signaling as pathways by which PDE11A deletion protects against ARCD.

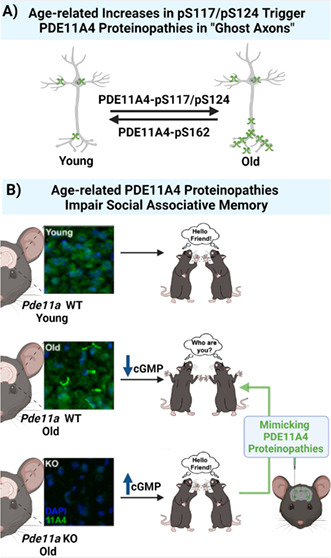

**Abbreviations**
2D‐DIGE: 2‐dimensional difference in gel electrophoresis
A.U.: arbitrary units
A/A: PDE11A4‐S117A/S124A
A: alanine
AC3: adenylyl cyclase 3
ADRD: Alzheimer’s Disease and related dementias
aLTM: associative long‐term memory
ARCD: age‐related cognitive decline
AStr: amygdalar‐striatal transition area
C: cysteine
cAMP: 3’,5’‐cyclic adenosine monophosphate
cGMP: 3’,5’‐cyclic guanosine monophosphate
CREB: cAMP response‐element binding protein
D/D: PDE11A4‐S117D/S124D
D: aspartate
dCA1: dorsal CA1
DHIPP: dorsal hippocampus
Fc: fold change
GFAP: glial fibrillary acidic protein
GFP: emerald green fluorescent protein
hPDE11A4: human PDE11A4
IBA‐1: ionized calcium binding adaptor molecule 1
IF: immunofluorescence
IHC: immunohistochemistry
KO‐O: old knockout
KO‐Y: young knockout
MAP2: microtubule associated protein
MBP: myelin basic protein
MDD: major depressive disorder
mPDE11A4: mouse PDE11A
MS: mass spectroscopy
NF‐L: neurofilament light chain
NSOR: non‐social odor recognition memory
PDE: 3’,5’‐cyclic nucleotide phosphodiesterase
PKA: protein kinase A
PKG: protein kinase G
PS: Ponceau stain
r.o.d.: relative optical density
rLTM: recognition long‐term memory
S117: PDE11A4 serine 117
S124: PDE11A4 serine 124
S162: PDE11A4 serine162
SOR: social odor recognition memory
STFP: social transmission of food preference
TBI: traumatic brain injury
vCA1: ventral CA1
VHIPP: ventral hippocampus
vSub: ventral subiculum
WT‐O: old wild‐type
WT‐Y: young wild‐type
Y: tyrosine

## 1 INTRODUCTION

After the age of 60, nearly all individuals experience some form of cognitive decline—particularly memory deficits—and no drugs prevent or reverse this loss (Abbott, 2012; Kelly, 2017). Even in the absence of dementia, age‐related cognitive decline (ARCD) increases healthcare costs and risk for disability (Plassman et al., 2008). ARCD is not a uniform process, with variability in symptom severity observed across individuals and cognitive domains (Abbott, 2012). For example, associative memories are more susceptible to ARCD in humans than are recognition memories (Bender et al., 2010; Bridger et al., 2017; Hargis & Castel, 2017; Hartman & Warren, 2005; Old & Naveh‐Benjamin, 2008; Overman & Becker, 2009; Troyer et al., 2011) for reasons that are not clear but may be related to deficient activation of anterior hippocampus (Dalton et al., 2013; Nordin et al., 2017) (a.k.a. ventral hippocampal formation in rodents, VHIPP). This lack of knowledge slows therapeutic development.

3′,5′‐cyclic adenosine monophosphate (cAMP) and 3′,5′‐cyclic guanosine monophosphate (cGMP) signaling are decreased in the aged and demented hippocampus (rodents and humans), particularly when there is a history of traumatic brain injury (TBI) (Bonkale et al., 1999; Titus et al., 2013; Zhang et al., 2014). Interestingly, cyclic nucleotide signaling deficits associated with neuropsychiatric and age‐related diseases of the brain can be more prominent in one subcellular compartment versus another (Bonkale et al., 1995; Bonkale et al., 1999; Chang et al., 2003; Fields et al., 1999; Kelly, 2018a; Rahman et al., 1997). Together, these findings suggest that select enzymes that generate (i.e., cyclases) and/or break down cAMP and cGMP (i.e., 3′,5′‐cyclic nucleotide phosphodiesterases, PDEs) not only change in expression and/or activity, but may also become ectopically localized (Houslay, 2010; Kokkonen & Kass, 2017). Such an ectopic localization could prove exceptionally deleterious since the discrete localization of these enzymes within specific subcellular domains allows a single cell to recognize and respond uniquely to multiple stimuli (Houslay, 2010; Kokkonen & Kass, 2017). In other words, where a cyclase or PDE is localized is just as important to its overall function as is its catalytic activity (Baillie et al., 2019).

Age‐related changes in cyclic nucleotide signaling may worsen cognition by promoting proteinopathies (a.k.a. proteopathies)—abnormalities in protein synthesis, post‐translational modification, folding, or deposition that occur with normal aging and age‐related diseases (Jo et al., 2020; Karanth et al., 2020; Moreno‐Gonzalez & Soto, 2011; Yanar et al., 2020). For example, reductions in hippocampal adenylyl cyclase correlate with the accumulation of amyloid plaques (Ohm et al., 1991), and increasing cyclic nucleotide signaling through the cAMP‐PKA and cGMP‐PKG pathways helps clear aggregated proteins that cause neurodegeneration (Goldberg et al., 2021; VerPlank et al., 2020; VerPlank & Goldberg, 2017). On the contrary, aberrant phosphorylation driven by cAMP/cGMP‐regulated kinases can drive the accumulation of tau and TDP‐43 in many neurodegenerative diseases (Gao et al., 2018; Moloney et al., 2021). Therefore, it is of great interest to determine how the dysregulation of cyclic nucleotides within specific brain regions and subcellular compartments may drive proteinopathies associated with aging and how that dysregulation may affect ARCD.

The age‐related decreases in hippocampal cyclic nucleotides described above are consistent with our observation that expression of PDE11A4 increases with age in both the mouse and rat hippocampus (Hegde, Capell, et al., 2016; Kelly et al., 2014). The PDE11A family is comprised of a single gene that is spliced into 4 isoforms, PDE11A1–4. The longest isoform, PDE11A4, is the isoform that is expressed in brain and is ~95% homologous across mouse, rat, and human (Yuasa, Kanoh, et al., 2001; Yuasa, Ohgaru, et al., 2001). All 4 isoforms are considered dual‐specific, in that they degrade both cAMP and cGMP; however, enzyme assays suggest PDE11A4 may hydrolyze cAMP with a higher *K*_m_ and *V*_max_ than cGMP (Weeks et al., 2005; Yuasa et al., 2000). PDE11A single nucleotide polymorphisms are associated with major depressive disorder (MDD) (Cabanero et al., 2009; Luo et al., 2009; Wong et al., 2006), suicide risk (Coon et al., 2013), sleep quality (Jones et al., 2019), antidepressant response in patients with MDD (Luo et al., 2009; Wong et al., 2006) (but see (Cabanero et al., 2009; Perlis et al., 2010)), and lithium response in patients with bipolar disorder (Couzin, 2008; Kelsoe, 2010; Mertens et al., 2015). It is, then, interesting to note that both MDD and BPD have been conceptualized as diseases of accelerated aging (Kinser & Lyon, 2013; Rizzo et al., 2014). PDE11A4 mRNA is strongly expressed in neurons of CA1, the subiculum, and the adjacently connected amygdalohippocampal area of the VHIPP (Hegde, Capell, et al., 2016; Kelly et al., 2010), with moderate expression in dorsal hippocampus (DHIPP) and little to no expression in other brain regions (Hegde, Capell, et al., 2016; Jäger et al., 2012; Kelly, 2015; Kelly et al., 2010; Pathak et al., 2017). Thus, PDE11A4 molecularly defines an exceptionally discrete neuronal population within a brain region key to associative memory, making it ripe for study in the context of ARCD.

Consistent with its enrichment in the VHIPP, PDE11A4 regulates preferences for social interactions as well as the consolidation of social memories (Hegde, Capell, et al., 2016; Hegde, Ji, et al., 2016; Kelly et al., 2010; Pilarzyk et al., 2019; Smith et al., 2021). The effect of PDE11A deletion on social memory in young adult mice is quite unique in that it triggers a transient amnesia that ultimately produces a stronger remote long‐term memory (LTM; i.e., intact short‐term memory, impaired recent LTM, improved remote LTM) (Pilarzyk et al., 2019). Further, we have shown PDE11A regulates signals important for social memory consolidation, including oxytocin signaling, glutamatergic signaling, calcium/calmodulin‐dependent kinase II signaling, CREB function, and protein synthesis (Hegde, Capell, et al., 2016; Kelly et al., 2010; Kelly et al., 2014; Pilarzyk et al., 2021; Smith et al., 2021). As such, here we test the hypothesis that age‐related increases in hippocampal PDE11A4 are conserved across species, occur in an ectopic subcellular compartment, and impair social associative memories.

## 2 RESULTS

### 2.1 Age‐related increases in PDE11A mRNA are conserved

**FIGURE 1:**
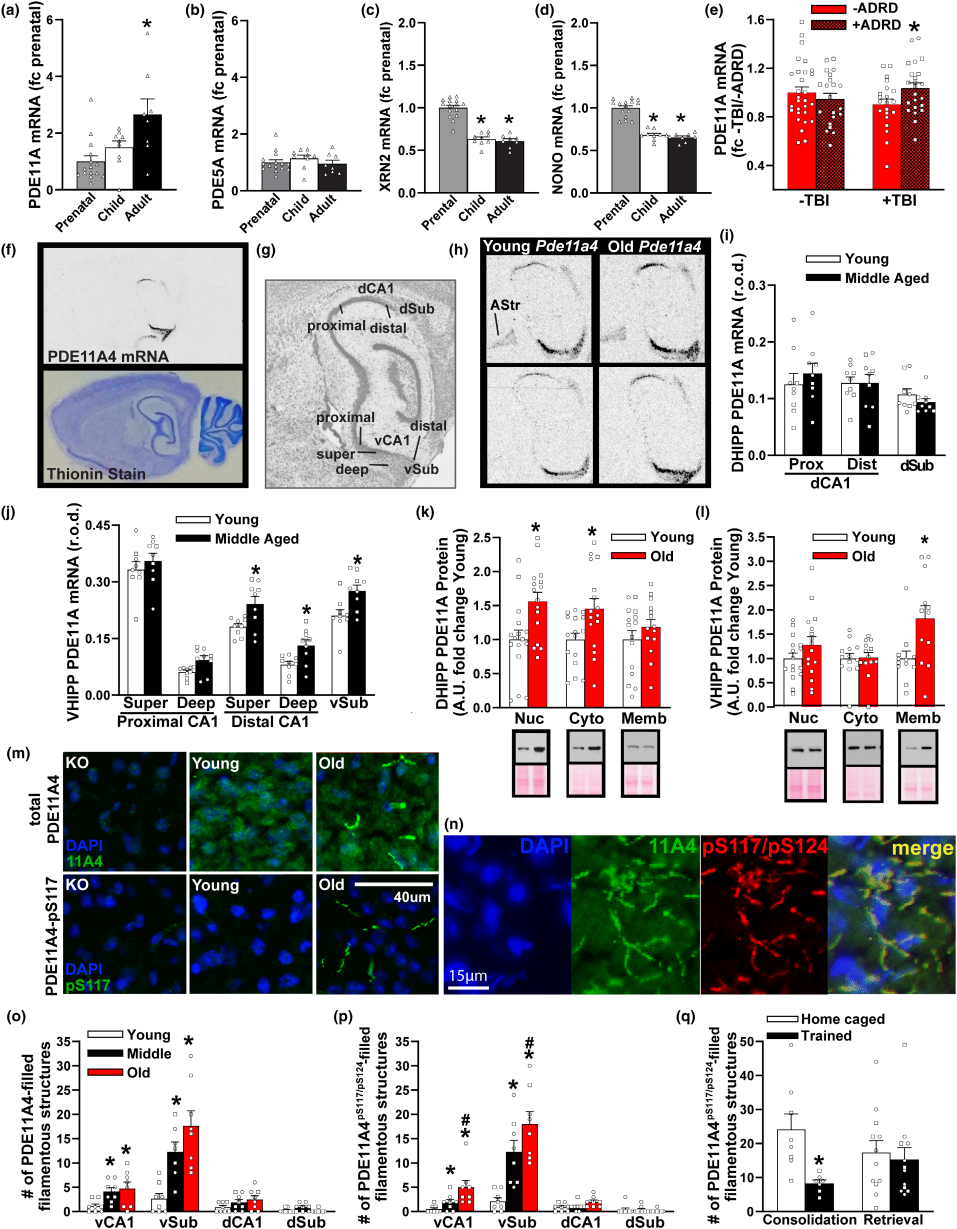
Hippocampal PDE11A mRNA expression uniquely increases across the human and rodent lifespan. (a) Data mined from the Allen Institute for Brain Sciences Brainspan database and expressed as a fold change (fc) of prenatal levels shows PDE11A mRNA expression in the human hippocampus robustly increases from the prenatal period (*n* = 15; sex not specified), to childhood (*n* = 9), through to adulthood (*n* = 8; H(2) = 9.48, *p* = 0.009; post hoc prenatal vs adult, *p* = 0.008). (b) in contrast, mRNA for PDE5A—The next closest related PDE—shows no change in expression (*F*[2,29] = 0.72, *p* = 0.496). This age‐related increase in PDE11A mRNA expression correlates with age‐related decreases in mRNA expression of (c) the nuclear protein p54^nrb^/NONO (*F*[2,29] = 66.29, *p* < 0.001; post hoc, each vs prenatal, *p* < 0.001) and (d) the exoribonuclease XRN2 (H(2) = 23.35, *p* < 0.001; post hoc, each vs. prenatal, *p* < 0.001), both of which work together to target PDE11A4 mRNA for degradation (Lu & Sewer, 2015). (e) Data mined from the Aging, Dementia, and Traumatic Brain Injury (TBI) database (expressed as fc ‐TBI/‐ADRD) shows that among elderly humans with a history of TBI, *Pde11a* mRNA is significantly higher in those that developed Alzheimer's disease or a related dementia (*n* = 10 M,13F) versus those that did not (*n* = 15 M,6F; effect of TBI × dementia: *F*[1,90] = 4.07, *p* = 0.047; post hoc + TBI/−dementia vs. +TBI/+dementia, *p* = 0.049). (f) To delineate where within the hippocampus age‐related increases in *Pde11a4* mRNA expression may be occurring, we turned to rodent studies. In situ hybridization shows *Pde11a4* mRNA in the brain is restricted to the extended hippocampal formation. (g) Robust expression is observed in all mice in CA1, the subiculum, and the amygdalohippocampal area. (h) To our surprise, a subset of male and female young and old mice also showed *Pde11a4* mRNA expression in the amygdalar‐striatal transition area (AStr; top) while others did not (bottom)—AStr staining had not been previously noted in the mouse brain with weaker probes (Hegde, Capell, et al., 2016; Kelly et al., 2010; Kelly et al., 2014). (i) Dorsal hippocampal subfields show no effect of age on PDE11A mRNA expression (*n* = 4 M,5F/age). (j) in contrast, select subfields of the ventral hippocampus show increased PDE11A4 mRNA expression in middle‐aged versus young mice (2‐way RM ANOVA on all ventral subfields failed normality), in particular the superficial and deep subfields within distal CA1 (effect of age: *F*[1,16] = 8.96, FDR‐*p* = 0.024) and the ventral subiculum (sub; *t*(16) = 2.97, FDR‐*p* = 0.024). (k) despite showing no age‐related changes in PDE11A mRNA expression, the DHIPP does exhibit an age‐related increase in PDE11A4 protein expression in the nuclear (Young, *n* = 9 M,7F; Old, *n* = 11 M,5F; *t*[30] = −2.91, *p* = 0.0068) and cytosolic fractions (Young, *n* = 9 M,7F; Old, *n* = 11 M,5F; *t*[30] = −2.54, *p* = 0.017) but not membrane fraction (Young, *n* = 9 M,6F; Old, *n* = 9 M,5F; *t*(27) = −1.07, *p* = 0.296). (l) In contrast, the ventral hippocampus shows no changes in PDE11A4 protein expression in the nuclear (Young, *n* = 9 M,7F; Old, *n* = 11 M,5F; *t*[30] = −1.34, *p* = 0.189) and cytosolic compartment (Young, *n* = 6 M,7F; Old, *n* = 8 M,5F; failed normality; rank sum test for effect of age: T[13,13] = 171.0, *p* = 0.837) but does show an age‐related increase in the membrane compartment (Young, *n* = 6 M,6F; Old, *n* = 7 M,5F; *t*[22] = −2.674, *p* = 0.014). (m) Immunofluorescence also revealed age‐related increases in VHIPP PDE11A4 protein are compartment specific, with PDE11A4 accumulating in filamentous structures we term ghost axons (see Figure 2). (n) PDE11A4 phosphorylated at serines 117 and 124 appears to be found exclusively in these ghost axons, and all PDE11A4 found in these ghost axons appears to be phosphorylated at S117/S124 as indicated by a complete overlap in staining patterns of the total and phospho‐specific antibodies. (o) Quantification (*n* = 4/sex/age) shows significant age‐related increases of PDE11A4‐filled structures in vCA1 (*F*[2,14] = 5.05, *p* = 0.022; post hoc vs Y: M *p* = 0.028, O *p* = 0.026) and vSub (*F*[2,13] = 13.55, *p* < 0.001; post hoc vs Y: M *p* = 0.005, O *p* < 0.001) as well as (p) PDE11A4‐pS117/pS124‐filled structures in vCA1 (fails normality; ANOVA on ranks for effect of age: *H*(2) = 12.62, *p* = 0.001; post hoc vs Young, each *p* < 0.05) and vSub (*F*[2,14] = 29.60, *p* < 0.001; post hoc vs Young, each *p* < 0.001). (q) Increased presence of these pS117/pS124‐PDE11A4‐filled ghost axons may be deleterious since normally memory consolidation following STFP training is associated with a reduction in their presence (home caged, *n* = 3 M,5F; trained, *n* = 3 M,4F; *t*[13] = 3.16, *p* = 0.007) although 24‐h memory retrieval is not (home caged, *n* = 4 M,8F; trained, *n* = 6 M,6F; failed normality; rank sum test for effect of age: *T*[12,12] = 162.0, *p* = 0.506). *vs prenatal, ‐ADRD, or young, *p* < 0.05–0.001; #vs. middle‐age, *p* < 0.05. Data plotted as individual points (females as circles, males as squares) and expressed as mean ± SEM. Histogram stretch, brightness, and/or contrast of images adjusted for graphical clarity. A.U., Arbitrary units; r.o.d., Relative optical density.

We previously showed that PDE11A4 mRNA and protein expression in the hippocampus increase across the lifespan in rodents (Hegde, Capell, et al., 2016; Kelly et al., 2014). Here, we assessed age‐related changes in expression in human HIPP by mining data from the Allen Institute for Brain Sciences Brainspan database. As in rodents, hippocampal PDE11A mRNA expression in humans increased across the lifespan (Figure 1a). The large 2.66‐fold increase in expression between the prenatal and adulthood period (18–44 years of age) is unique to PDE11A, as PDE5A—the closest related PDE—shows no such increase in hippocampus (Figure 1b). Further, our previous work on the next closest‐related PDEs shows only a modest 0.36‐fold increase in PDE2A mRNA along with a 0.42‐fold and 0.54‐fold decrease in PDE9A and PDE10A mRNA, respectively (Farmer et al., 2020; Patel et al., 2018). Next, we determined if this age‐related increase in PDE11A mRNA expression might be related to increased transcript stability by examining expression of p54^nrb^/NONO and the exoribonuclease XRN2, both of which form a complex with PDE11A4 mRNA to target it for degradation (Lu & Sewer, 2015). In concert with the age‐related increases in PDE11A mRNA expression, we see that p54^nrb^/NONO and XRN2 mRNA decrease (Figure 1c–d). Given evidence that TBI can exacerbate age‐related decreases in hippocampal cAMP levels (Titus et al., 2013), we next mined data from the Aging, Dementia, and Traumatic Brain Injury database that includes data from a group of 75+‐year‐old patients characterized with regard to their history of TBI and dementia. Among elderly humans with a history of TBI, hippocampal PDE11A mRNA expression was significantly higher in those that developed dementia versus those that did not (Figure 1e). In contrast, there was no difference between TBI patients with vs. without dementia in terms of PDE2A (−dementia, 0.95 ± 0.04; +dementia 0.90 ± 0.03), PDE5A (−dementia, 0.96 ± 0.02; +dementia, 0.97 ± 0.03), or PDE10A expression (−dementia, 0.83 ± 0.03; +dementia 0.86 ± 0.04). Together, these data suggest that PDE11A expression uniquely increases with age in the human hippocampus in part due to increases in transcript stability, and that these age‐related increases can be exacerbated in those with dementia and a history of TBI.

### 2.2 Age‐related increases in PDE11A4 protein expression occur in a compartment‐specific manner

**FIGURE 2:**
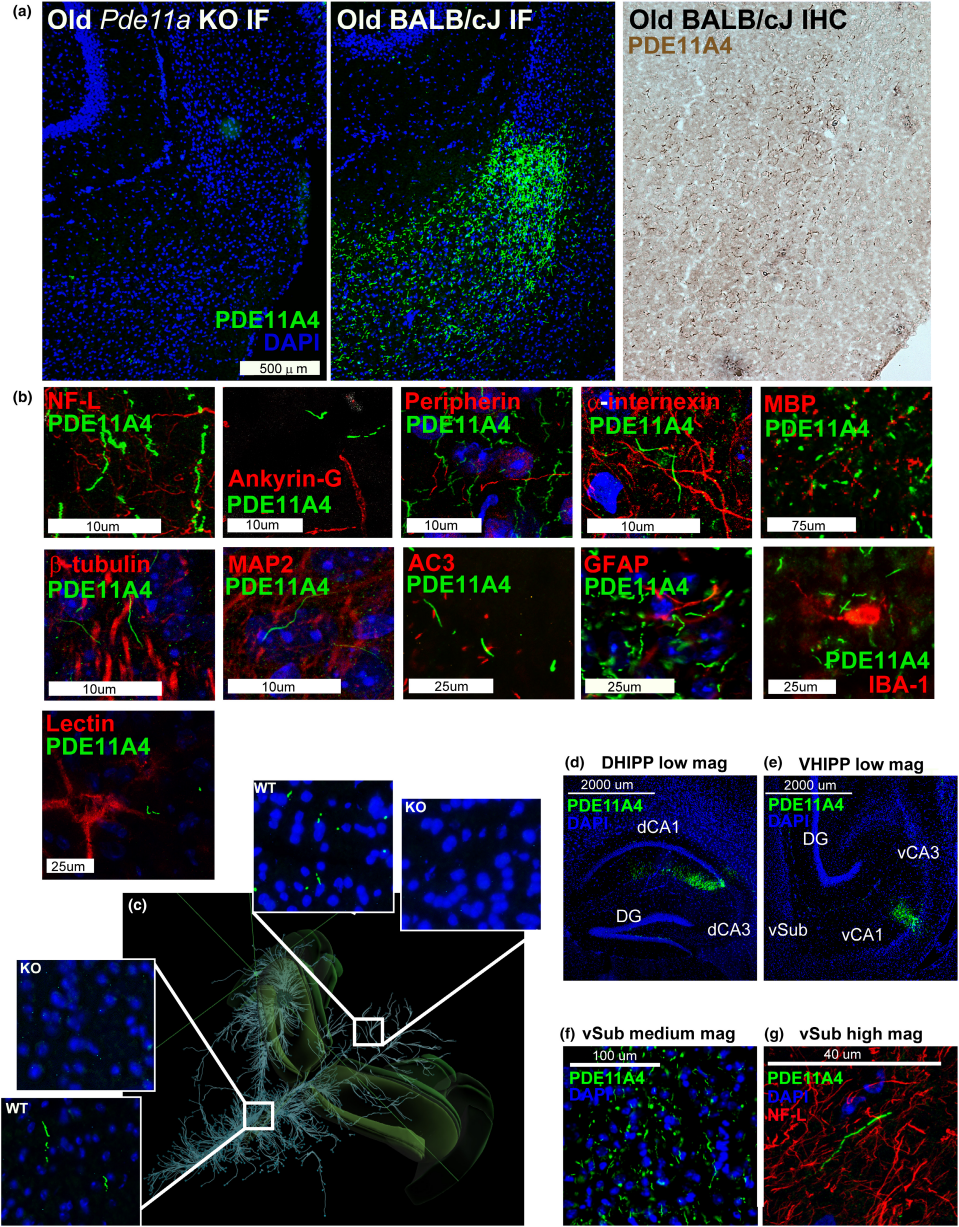
Age‐related increases in PDE11A4 protein expression accumulate ectopically in ghost axons. (a) Immunofluorescence with the PDE11A4‐pS117/pS124 antibody (green; nuclear marker DAPI shown in blue) shows no signal in the ventral subiculum (vSub) of a *Pde11a* KO mouse on a BALB/cJ background but does show the accumulation of PDE11A4‐filled filamentous structures throughout the ventral subiculum (vSub) of a *Pde11a* WT mouse on a BALB/cJ background. The structures can also be visualized in a WT mouse using immunohistochemistry. (b) Staining for PDE11A4‐filled structures appears very much like that of intermediate filaments in terms of diameter and tortuosity; however, PDE11A4‐filled structures fail to colocalize with markers for axons (NF‐L, Ankryin‐G, peripherin, internexin, MBP), neurons (tubulin), dendrites (MAP2), cilia (AC3), glia (IBA‐1–microglia, GFAP–astrocytes) or perineuronal nets (lectin). (c) Given these structures look very much like axonal markers, coupled with the fact that several age‐related diseases are associated with the aberrant accumulation of proteins in axons (Moloney et al., 2021; Uchihara, 2014) we determined if such PDE11A4‐filled structures could be found in other terminal fields of vCA1 projections that themselves do not express PDE11A4 mRNA (terminal fields defined by Allen Institute for Brain Science's mouse brain connectivity; projections shown from top left perspective of mouse brain). Indeed, PDE11A4‐filled structures can be found in such terminal fields (shown: Hypothalamic nuclei in left insets, colliculi in right insets), strongly suggesting PDE11A4 is accumulating in terminal regions of axons projecting from CA1. (d) To confirm this, we injected GFP‐tagged mPDE11A4 into the dorsal and ventral CA1 (dCA1, vCA1) of *Pde11a* KO mice on the C57BL/6J background. Despite the fact that PDE11A4 expression was only being generated in CA1, (e) PDE11A4‐filled structures emerged in vSub consistent with an accumulation of the protein within axons projecting from CA1 to vSub. (f) At medium magnification and (g) high magnification, like endogenous PDE11A4‐filled structures, recombinant PDE11A4‐filled structures appear identical in size and shape to NF‐L, but fail to colocalize with NF‐L. These studies, along with electron microscopy results (Figure S3), suggest high levels of PDE11A4 expression leads to an accumulation of PDE11A4 in axons that either occludes co‐localization of other axonal protein or possibly leads to the degeneration of the surrounding axon as occurs with tau ghost tangles (Moloney et al., 2021; Uchihara, 2014), hence the adoption of the term ghost axons herein. NF‐L, neurofilament light chain; MBP, myelin basic protein; MAP2, microtubule associated protein; AC3, adenylyl cyclase 3; IBA‐1, ionized calcium binding adaptor molecule 1; GFAP, glial fibrillary acidic protein. Histogram stretch, brightness, and/or contrast of images adjusted for graphical clarity

As noted above, PDEs are discretely localized to specific brain regions and subcellular domains (Houslay, 2010; Kokkonen & Kass, 2017). At the regional level, PDE11A4 mRNA is restricted to the extended hippocampal formation, with enrichment in VHIPP versus DHIPP. At the subcellular level, PDE11A4 is enriched in the cytosol vs. membrane and nucleus, which stands in contrast to PDE2A and PDE10A—the two most closely related PDEs expressed in hippocampus—both of which are greatly enriched in the membrane versus cytosol (Kelly, 2018b). As such, we determined if age‐related increases in PDE11A4 were uniformly distributed or were restricted to discrete, potentially ectopic, compartments. In situ hybridization shows that PDE11A4 mRNA in the middle‐aged mouse brain remains largely restricted to the extended hippocampal formation (Figure 1f), including CA1, subiculum, the AHi, and—in a subset of male and female mice—the amygdalar‐striatal transition area (AStr) that sits just anterior to the hippocampus (Figure 1h). Note that expression in the AStr had not been noted with previous weaker probes (Hegde, Capell, et al., 2016; Kelly et al., 2010; Kelly et al., 2014). In DHIPP, there was no effect of age on PDE11A4 mRNA expression (Figure 1i); however, protein expression was increased in the nuclear and cytosolic compartments of old versus young mice (Figure 1k; see Figure S1A for validation of biochemical fractionations). In contrast, select subfields of the VHIPP did show increased PDE11A mRNA expression in middle‐aged versus young mice (i.e., the superficial and deep subfields within distal CA1 and the ventral subiculum; Figure 1j), and PDE11A4 protein was increased only in the membrane compartment of old versus young mice (Figure 1l). Native gels suggest that PDE11A4 is not associated with the membrane directly (e.g., via palmitoylation), but rather via protein–protein interactions (Figure S1B). Phosphorylation is a post‐translational modification known to alter PDE protein–protein interactions (Baillie et al., 2019). As such, we validated antibodies that recognize phosphorylation of PDE11A4 serines 117 and 124 (Figure S1C). Immunofluorescence with antibodies that detect all PDE11A4 (Figure 1m, n), PDE11A4‐pS117 (not shown but see Source Data), or PDE11A4‐pS117/pS124 (Figure 1m, n; see Figure S1 for antibody validation) confirmed the differential compartment‐specific localizations of PDE11A4 protein in the aged DHIPP versus VHIPP, with PDE11A4 in VHIPP accumulating in filamentous structures that are rarely seen in young C57BL/6J mice (Figure 1o, p). This increased accumulation of PDE11A4 in the aged VHIPP is also observed in BALB/cJ (Figure S2A, C) and 129S6/SvEv mice (Figure S2D). We term these PDE11A4‐filled structures ghost axons (see Figure 2) and find they are enriched for PDE11A4 that is phosphorylated at serine 117 and 124 (pS117/pS124; Figure 1m bottom and 1n). This age‐related increase in PDE11A4‐pS117/pS124‐filled structures may be deleterious since associative memory consolidation in the social transmission of food preference assay—but not memory retrieval—normally triggers a reduction in their presence (Figure 1q).

### 2.3 Preventing age‐related increases in PDE11A is sufficient to prevent cognitive decline of social associative memory

**FIGURE 3:**
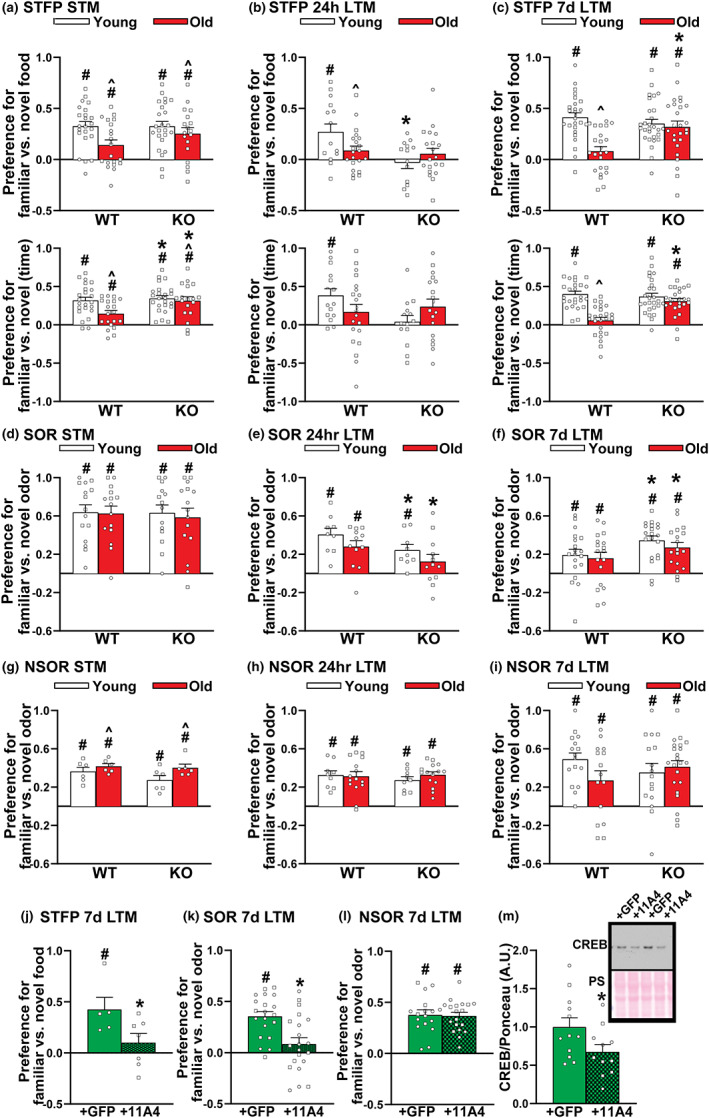
Preventing age‐related increases in PDE11A4 is sufficient to prevent age‐related cognitive decline of social associative memories (aLTMs) due to the acute loss of PDE11A4 in the aged hippocampus. (a) WT‐Y (*n* = 16 M,7F), KO‐Y (*n* = 18 M,6F), WT‐O (*n* = 13 M,7F), and KO‐O mice (*n* = 15 M,3F) each showed STFP STM when memory was defined by the amount of food eaten (top graphs) or the time spent eating the food (bottom graphs); however, old WT mice in particular demonstrated weaker memory than young mice (food effect of age: *F*(1,81) = 6.59, *p* = 0.012; time effect of age: *F*(1,81) = 5.02, *p* = 0.028; time effect of genotype: *F*(1,81) = 5.02, *p* = 0.028). (b) 24 h after training, only WT‐Y mice (*n* = 7 M,8F) showed STFP memory, with KO‐Y (*n* = 8 M,6F), KO‐O (*n* = 8 M,12F), and WT‐O mice (*n* = 8 M,13F) performing significantly worse (food effect of genotype × age: *F*(1,62) = 4.31, *p* = 0.042; post hoc vs. WT‐Y: WT‐O *p* = 0.028, KO‐Y *p* = 0.002, KO‐O *p* = 0.029; time effect of genotype x age: *F*(1,60) = 3.83, *p* = 0.055). (c) 7 days after STFP training, WT‐Y (*n* = 14 M,12F), KO‐Y (*n* = 18 M,10F), and KO‐O mice (*n* = 14 M,12F) exhibit stronger remote aLTM than WT‐O mice (*n* = 13 M,11F; food effect of genotype × age: *F*(1,96) = 11.68, *p* = 0.0009; post hoc: WT‐O vs each group, *p* < 0.001; time effect of genotype x age: *F*(1,95) = 13.34, *p* = 0.0004; post hoc: WT‐O vs each group, *p* = 0.0001). (d) all groups demonstrated strong STM for SOR, including WT‐Y (*n* = 8 M,7F), KO‐Y (*n* = 7 M,7F), WT‐O (*n* = 8 M,7F), and KO‐O (*n* = 8 M,7F) with no difference among groups. (e) 24 h after SOR training, WT‐Y (*n* = 4 M,5F) and WT‐O mice (*n* = 7 M,6F) exhibit significantly stronger rLTM than KO‐Y (*n* = 5 M,4F) and KO‐O mice (*n* = 6/sex; effect of genotype: *F*[1,38] = 5.67, *p* = 0.022). (f) 7 days after SOR training, however, KO‐Y (*n* = 10 M,8F) and KO‐O mice (*n* = 10 M,8F) exhibit stronger rLTM than WT‐Y (*n* = 9/sex) and WT‐O mice(*n* = 11 M,7F; (*F*[1,67] = 5.25, *p* = 0.025) consistent with our previous observation in young KO mice (Pilarzyk et al., 2019). (g) each group showed strong NSOR STM, with old mice slightly outperforming young mice (*n* = 3/sex/age/genotype; effect of age: *F*[1,20] = 5.08, *p* = 0.036). (h) There were no differences among groups, however, for NSOR recent rLTM (WT‐Y *n* = 4 M,5F; KO‐Y *n* = 5/4F; WT‐O *n* = 6 M,9F; KO‐O *n* = 6 M,9F) or (i) NSOR remote rLTM (WT‐Y *n* = 7 M,9F; KO‐Y *n* = 9 M,7F; WT‐O *n* = 9 M,6F; KO‐O *n* = 13 M,9F). (j**)** Next, we determined if overexpressing PDE11A4 in dorsal and ventral CA1 of old KO mice (see Figure 2D for expression pattern observed 2 weeks following injection and Figure S8A for map of expression pattern observed >2 months injections) would be sufficient to eliminate their protective phenotype by infusing a lentivirus expressing either GFP alone or a GFP‐tagged mPDE11A4 (Pilarzyk et al., 2019) that exhibits robust catalytic activity (*n* = 8 biological replicates of HT‐22 cells/group; cGMP: (*t*(14) = −16.58, *p* < 0.001; cAMP: *T*(14) = −14.61, *p* < 0.001). Relative to infusion of GFP only, infusion of PDE11A4 significantly impaired remote aLTM for STFP (KO‐GFP *n* = 2 M,3F; 11A4 *n* = 4 M/3F; *t*(10) = −2.24, *p* = 0.049) and (k) SOR (KO‐11A4 *n* = 10 M,12F; KO‐GFP *n* = 8 M,11F; effect of virus: *F*[1,37] = 11.47, *p* = 0.002), (l) but not NSOR (KO‐11A4 *n* = 8 M,11F; KO‐GFP *n* = 7 M/10F; effect of virus: *F*[1,32] = 0.09, *p* = 0.763). (m) Overexpressing PDE11A4 in KOs is also sufficient to elicit an aging‐like biochemical phenotype in the VHIPP, namely decreased CREB in the nucleus (GFP *n* = 6 M,6F; 11A4 *n* = 6 M,5F; *t*(21) = 2.11, *p* = 0.047). *vs WT or GFP, *p* = 0.049 to <0.001, ^vs. Young, *p* = 0.028 to <0.001; #has memory (i.e., significantly >0; see Table 1 for individual statistics), *p* = 0.018 to <0.001. Data plotted as individual points (females as circles, males as squares) and expressed as mean ± SEM. Brightness and/or contrast of images adjusted for graphical clarity. PS, Ponceau stain

Human studies show that associative memories involving verbal and/or non‐verbal stimuli (e.g., faces and names) are more susceptible to age‐related cognitive decline than are recognition memories for individual items (Bender et al., 2010; Bridger et al., 2017; Hargis & Castel, 2017; Hartman & Warren, 2005; Old & Naveh‐Benjamin, 2008; Overman & Becker, 2009; Troyer et al., 2011). We were able to recapitulate a differential sensitivity of aLTMs vs recognition LTMs (rLTMs) in C57BL/6J mice using social memory tasks (Figure S4). To test the hypothesis that ARCD of aLTMs is driven by the age‐related increases in PDE11A4 expression described above, we compared male and female young vs. old *Pde11a* WT and KO mice in social transmission of food preference (STFP). [Note that female *Pde11a* WT and KO mice are indistinguishable in terms of cycle length or reproductive senescence (Figure S5), consistent with our previous report showing no gross peripheral pathology in middle‐aged KO mice (Kelly et al., 2010).] Our initial focus on social aLTM stems from the fact that in young adult mice, PDE11A4 regulates social behaviors and social memory, but not non‐social memory (Hegde, Capell, et al., 2016; Hegde, Ji, et al., 2016; Kelly et al., 2010; Pilarzyk et al., 2019; Smith et al., 2021). To assess the specificity of effects for aLTMs, we also tested these mice for social odor recognition memory (SOR) and non‐social odor recognition memory (NSOR). In all assays, we tested short‐term memory (STM), recent LTM 24 h after training, and remote LTM 7 days after training given that we previously showed deletion of PDE11A produces transient amnesia for social memories in adolescent and young adult mice (e.g., intact STM, impaired recent LTM, and intact/improved remote LTM). Old *Pde11a* WT mice exhibited STM for STFP but it was weaker than that of young WT mice (Figure 3a, Figure S6A; Table 1). This deficit worsened as time progressed with old WT mice failing to demonstrate any aLTM for STFP at the 24‐h and 7‐day timepoints (Figure 3b, c, Figure S6B, C). Despite the fact that both young and old *Pde11a* KO mice demonstrate a transient amnesia for recent aLTM 24 hours after STFP training (Figure 3b, Figure S6B), old KO mice show stronger STM and remote aLTM 7 days after STFP training relative to old WT mice (Figure 3a, c, Figure S6A–C). Middle‐aged *Pde11a* HT and KO mice similarly show enhanced remote aLTM relative to middle‐aged WT mice 7 days after STFP training (Figure S6F), despite middle‐aged KO mice exhibiting no recent aLTM (Figure S6B). Consistent with our observations in the C57BL/6J mice (Figure S4), there was no ARCD of SOR or NSOR in any genotype at any time point; however, both young and old *Pde11a* KO mice exhibited a transient amnesia for SOR in line with our previous work in young mice (Figure 3d–f) (Pilarzyk et al., 2019). Importantly, the effect of genotype on these memory tasks cannot be explained by performance aspects of these assays. Although old mice consistently ate less food during STFP and spent less time investigating during SOR than young mice, and females ate more food during STFP and spent more time investigating during SOR than males as we previously described (Pilarzyk et al., 2019), there were no main effects or interactions with the effect of genotype (Table S1). This suggests the effects of age and genotype on memory versus food consumption and total time investigating social odors are dissociable. Thus, old *Pde11a* KO mice exhibit transient amnesia for social but not non‐social memories much like young KO mice, and the ability of *Pde11a* deletion to prevent ARCD of STFP aLTMs is related to the associative nature of the memory as opposed to age‐related changes in recognizing the social or non‐social components.

**TABLE 1:** To determine whether or not a given group exhibited a signifciant memory in a given task at a given time point, preference ratios for each group were analyzed by one‐sample t‐test to determine if their performance significantly differed from chance (i.e., 0), with resulting *p*‐values corrected for multiple comparisons within an experiment using false discovery rate correctio (FDR)

Datasets	WT‐Y	WT‐O	KO‐Y	KO‐O
Figure 3a food STFP STM	*t*(22) = 7.29, FDR‐*p* < 0.001	*t*(19) = 2.84, FDR‐*p* = 0.011	*t*(23) = 6.81, FDR‐*p* < 0.001	*t*(17) = 4.29, FDR‐*p* < 0.001
Figure 3a time STFP STM	*t*(22) = 7.42, FDR‐*p* < 0.001	*t*(19) = 3.63, FDR‐*p* = 0.002	*t*(23) = 9.38, FDR‐*p* < 0.001	*t*(17) = 5.92, FDR‐*p* < 0.001
Figure 3b food STFP 24 h LTM	*t*(14) = 3.48, FDR‐*p* = 0.015	*t*(20) = 1.97, FDR‐*p* = 0.126	*t*(13) = 0.57, FDR‐*p* = 0.574	*t*(19) = 1.09, *p* = 0.388
Figure 3b time STFP 24 h LTM	*t*(14) = 4.51, FDR‐*p* = 0.002	*t*(20) = 1.72, FDR‐*p* = 0.135	*t*(13) = 0.49, FDR‐*p* = 0.635	*t*(17) = 2.45, FDR‐*p* = 0.051
Figure 3c food STFP 7 d LTM	*t*(25) = 9.35, FDR‐*p* < 0.001	*t*(23) = 1.93, FDR‐*p* = 0.066	*t*(27) = 8.44, FDR‐*p* < 0.001	*t*(25) = 5.66, FDR‐*p* < 0.001
Figure 3c time STFP 7 d LTM	*t*(25) = 11.12, FDR‐*p* < 0.001	*t*(24) = 1.46, FDR‐*p* = 0.157	*t*(26) = 8.53, FDR‐*p* < 0.001	*t*(24) = 8.7, FDR‐*p* < 0.001
Figure 3d SOR STM	*t*(14) = 8.12, FDR‐*p* < 0.001	*t*(14) = 8.11, FDR‐*p* < 0.001	*t*(13) = 7.60, *p* < 0.001	*t*(14) = 6.09, FDR‐*p* < 0.001
Figure 3e SOR 24 h LTM	*t*(8) = 6.06, FDR‐*p* = 0.001	*t*(11) = 4.66, FDR‐*p* = 0.001	*t*(8) = 4.23, FDR‐*p* = 0.004	*t*(11) = 1.72, FDR‐*p* = 0.113
Figure 3f SOR 7 d LTM	*t*(18) = 3.19, FDR‐*p* = 0.007	*t*(17) = 2.44, FDR‐*p* = 0.026	*t*(19) = 7.46, FDR‐*p* < 0.001	*t*(17) = 5.33, FDR‐*p* < 0.001
Figure 3g NSOR STM	*t*(5) = 8.06, FDR‐*p* < 0.001	*t*(5) = 14.83, FDR‐*p* < 0.001	*t*(5) = 5.64, *p* < 0.001	*t*(5) = 10.53, FDR‐*p* < 0.001
Figure 3h NSOR 24 h LTM	*t*(8) = 10.57, FDR‐*p* < 0.001	*t*(14) = 9.25, FDR‐*p* < 0.001	*t*(8) = 7.43, *p* < 0.001	*t*(14) = 10.58, FDR‐*p* < 0.001
Figure 3i NSOR 7 d LTM	*t*(15) = 7.39, FDR‐*p* < 0.001	*t*(14) = 2.68, FDR‐*p* = 0.018	*t*(15) = 3.66, *p* = 0.003	*t*(21) = 6.38, FDR‐*p* < 0.001
Figure S7C SOR 7 d LTM retest	*t*(22) = 4.03, FDR‐*p* < 0.001	*t*(28) = 6.13, FDR‐*p* < 0.001	*t*(22) = 4.27, FDR‐*p* < 0.001	*t*(27) = 6.25, FDR‐*p* < 0.001
		**KO‐GFP‐PDE11A4**		**KO‐GFP**
Figure 3k STFP 7 d LTM		*t*(6) = 1.13, *p* = 0.301		*t*(4) = 3.61, FDR‐*p* = 0.044
Figure 3l SOR 7 d LTM		*t*(20) = 1.38, *p* = 0.183		*t*(19) = 7.73, FDR‐*p* < 0.001
Figure 3m NSOR 7 d LTM		*t*(18) = 10.32, FDR‐*p* < 0.001		*t*(16) = 8.58, FDR‐*p* < 0.001
	**Young**	**Old**		
Figure S4A C57 food STFP 7 d LTM	*t*(22) = 385; FDR‐*p* = 0.012	*t*(22) = −0.28; FDR‐*p* = 0.781		
Figure S4B C57 SOR 7 d LTM	*t*(21) = 3.94, FDR‐*p* < 0.001	*t*(22) = 2.64, FDR‐*p* = 0.015		
Figure S4C C57 NSOR 7 d LTM	*t*(13) = 8.21, FDR‐*p* < 0.001	*t*(13) = 8.02, FDR‐*p* < 0.001		
		**WT‐MA**	**HT‐MA**	**KO‐MA**
Figure S6A food STFP STM		*t*(12) = 2.71, FDR‐*p* = 0.028	*t*(7) = 0.62, FDR‐*p* = 0.554	*t*(14) = 8.77, FDR‐*p* < 0.001
Figure S6A time STFP STM		*t*(12) = 2.70, FDR‐*p* = 0.029	*t*(7) = 0.49, FDR‐*p* = 0.638	*t*(14) = 9.57, FDR‐*p* < 0.001
Figure S6B food STFP 24 h LTM		*t*(14) = 4.42, FDR‐*p* = 0.002	*t*(7) = 1.57, FDR‐*p* = 0.241	*t*(14) = 1.24, FDR‐*p* = 0.237
Figure S6B time STFP 24 h LTM		*t*(14) = 6.27, FDR‐*p* < 0.001	*t*(7) = 2.19, FDR‐*p* = 0.097	*t*(14) = 1.36, FDR‐*p* = 0.194
Figure S6C food 7 d LTM		*t*(13) = −1.93, FDR‐*p* = 0.113	*t*(6) = 1.54, FDR‐*p* = 0.17	*t*(14) = 4.49, FDR‐*p* = 0.002
Figure S6C time 7 d LTM		*t*(13) = –1.72, FDR‐*p* = 0.164	*t*(6) = 0.82, FDR‐*p* = 0.445	*t*(14) = 5.58 FDR‐*p* < 0.001

### 2.4 Mimicking age‐related overexpression of PDE11A4 in *Pde11a* KO is sufficient to trigger aging‐like deficits in biochemical and behavioral endpoints

To determine whether *Pde11a* KO mice are protected against age‐related cognitive decline specifically due to the loss of PDE11A4 signaling in the aged brain or due to compensatory changes across the lifespan, we determined if overexpressing PDE11A4 in old KO mice would be sufficient to eliminate their protective phenotype. Infusions of a lentivirus expressing either GFP alone or a GFP‐tagged mPDE11A4 (Pilarzyk et al., 2019) were targeted to dorsal and ventral CA1 of KO mice (KO‐GFP vs. KO‐11A4, respectively; Figure 2d and Figure S8A) since our previous work identified this as the hippocampal subfield within which PDE11A4 critically regulates social memory consolidation (Pilarzyk et al., 2019). The recombinant GFP‐tagged mPDE11A4 behaves similarly to endogenous mPDE11A4 in that it exhibits robust cAMP/cGMP‐hydrolytic activity (Figure 1j), undergoes phosphorylation at S117/S124 (Figure S8b, c), and traffics to relevant compartments of the hippocampus (i.e., the cell body and dendritic layer of dorsal and ventral CA1, Figure 1d; ghost axons vSub, Figure 1e). As expected, KO‐GFP mice demonstrated strong remote LTM for STFP, much like unsurgerized KO mice, but the overexpression of PDE11A4 in the aged KO CA1 was sufficient to reverse this protective phenotype (Figure 3k). There was no difference between treatments in terms of the total amount of food eaten during the test (KO‐GFP, 1.07 ± 0.11 gm; KO‐11A4, 1.01 ± 0.14 gm; *t*[11] = −0.34, *p* = 0.737). Although KO‐GFP and KO‐11A4 mice learned equally well during training for SOR (Figure S8D), KO‐GFP mice demonstrated strong remote rLTM for SOR but KO‐11A4 did not (Figure 3l) as previously reported for young adult mice (Pilarzyk et al., 2019). In contrast, KO‐GFP and KO‐11A4 mice showed both strong learning (Figure S8E) and remote rLTM for NSOR (Figure 3M). Overexpressing PDE11A4 in KOs was also sufficient to elicit aging‐like decreases in VHIPP CREB activity (Kelly, 2018a), as indicated by reduced nuclear localization of CREB (Figure 3n). Together, these data suggest the protective phenotype observed in old *Pde11a* KO mice is directly related to the absence of PDE11A4 signaling in the aged brain as opposed to a compensatory mechanism triggered by the chronic loss of PDE11A across the lifespan.

### 2.5 RNA sequencing and phosphoproteomics of the VHIPP implicate many of the same pathways in the protective effect of PDE11A deletion

To gain insight into how the absence of PDE11A4 in the adult hippocampus provides protection against ARCD, we conducted an RNA sequencing study in one cohort of mice followed by a confirmatory phosphoproteomic study in a second cohort of mice using VHIPP samples from aged *Pde11a* KO and WT mice. Importantly, changes in gene expression and protein phosphorylation were enriched in many of the same pathways (Table 2). Consistent with PDE11A4 regulating cyclic nucleotide signaling and social behaviors, both studies identified the cGMP‐PKG signaling pathway and oxytocin signaling pathway. Interestingly, both studies also identified the calcium signaling, glutamatergic synapse, cholinergic synapse, and Alzheimer's disease pathways, all of which have been implicated in ARCD and/or dementia (see *Discussion*). Given the age‐related accumulation of PDE11A4 in ghost axons, it is also interesting to note that both studies identified pathways related to the axon and axon guidance, regulation of transport, chemical synaptic transmission, as well as the synapse, presynapse, and synaptic vesicle. An effect of PDE11A deletion on these latter pathways is consistent with PDE11A4 colocalizing with adaptin, a marker of intracellular transport vesicles (Figure S9C). Additional age‐related pathways of note that were identified by one technique but not the other included longevity regulating pathway (RNA sequencing, strength 0.46, FDR‐*p* = 0.001), longevity regulating pathway‐multiple species (RNA sequencing, strength 0.55, FDR‐*p* < 0.001) and cellular senescence (phosphoproteomics, strength 1.25, FDR‐*p* = 0.008).

**TABLE 2:** Pathways identified as significantly enriched for both mRNA changes (a total of 1597 identified) and phosphoproteomic changes (a total of 22 identified) in VHIPP of old *Pde11a* KO vs WT mice

#Term ID	Term description	Background gene count	RNA sequencing pathway analyses results			Phosphoproteomics pathway analyses		
			observed gene count	strength	FDR‐*p*	observed gene count	strength	FDR‐*p*
*KEGG Pathways*								
mmu04020	Calcium signaling pathway	180	37	0.45	9.17E−06	4	1.35	0.001
mmu04723	Retrograde endocannabinoid signaling	145	26	0.39	0.00097	4	1.44	0.001
mmu04724	Glutamatergic synapse	113	23	0.45	0.00067	3	1.42	0.0035
mmu04921	Oxytocin signaling pathway	149	20	0.27	0.049	3	1.3	0.0067
mmu04022	cGMP−PKG signaling pathway	164	32	0.43	7.29E−05	3	1.26	0.0079
mmu05010	Alzheimer's disease	167	22	0.26	0.0454	3	1.26	0.0079
mmu04971	Gastric acid secretion	72	12	0.36	0.049	2	1.44	0.0169
mmu04727	GABAergic synapse	87	19	0.48	0.00097	2	1.36	0.0211
mmu05032	Morphine addiction	91	26	0.6	4.95E−06	2	1.34	0.0219
mmu04713	Circadian entrainment	95	26	0.58	7.62E−06	2	1.32	0.0227
mmu04725	Cholinergic synapse	112	24	0.47	0.00025	2	1.25	0.0241
mmu05225	Hepatocellular carcinoma	168	23	0.28	0.0285	2	1.08	0.0382
mmu04360	Axon guidance	174	33	0.42	7.65E−05	2	1.06	0.0395
*GO Processes*								
GO:0050896	Response to stimulus	6616	664	0.14	7.61E−19	17	0.41	0.0034
GO:0010243	Response to organonitrogen compound	867	105	0.22	3.86E−05	7	0.91	0.0052
GO:0006950	Response to stress	2899	248	0.07	0.043	11	0.58	0.0072
GO:0051049	Regulation of transport	1782	202	0.19	9.30E−08	9	0.7	0.0072
GO:0009987	Cellular process	12459	1138	0.1	6.33E−29	21	0.23	0.0093
GO:0072347	Response to anesthetic	81	14	0.38	0.0441	3	1.57	0.01
GO:0010035	Response to inorganic substance	505	61	0.22	0.0035	5	1	0.0118
GO:0043279	Response to alkaloid	115	18	0.33	0.0376	3	1.42	0.0182
GO:0007268	Chemical synaptic transmission	321	55	0.37	2.54E−06	4	1.1	0.0183
GO:0010038	Response to metal ion	344	42	0.23	0.0193	4	1.07	0.0183
GO:0032501	Multicellular organismal process	5888	643	0.18	6.03E−27	14	0.38	0.0183
GO:0042493	Response to drug	926	114	0.23	8.11E−06	6	0.81	0.0183
GO:0051128	Regulation of cellular component organization	2337	272	0.21	7.00E−12	9	0.59	0.0183
*GO Component*								
GO:0120025	Plasma membrane bounded cell projection	2172	273	0.24	1.22E−15	12	0.74	2.11E−05
GO:0043005	Neuron projection	1429	207	0.3	4.05E−17	10	0.85	2.11E−05
GO:0005737	Cytoplasm	9909	858	0.08	3.08E−10	21	0.33	2.54E−05
GO:0008021	Synaptic vesicle	183	24	0.26	0.0465	5	1.44	3.27E−05
GO:0045202	Synapse	968	150	0.33	2.44E−14	8	0.92	6.43E−05
GO:0005622	Intracellular	12,462	1099	0.09	6.33E−20	22	0.25	7.08E−05
GO:0030425	Dendrite	694	117	0.37	2.56E−13	7	1	7.08E−05
GO:0098793	Presynapse	429	60	0.29	9.86E−05	6	1.15	7.08E−05
GO:0032991	Protein−containing complex	4701	479	0.15	2.56E−13	14	0.47	0.00023
GO:0005829	Cytosol	3326	283	0.07	0.0249	12	0.56	0.00023
GO:0098796	Membrane protein complex	1009	119	0.21	1.56E−05	7	0.84	0.00035
GO:0005623	Cell	14,044	1233	0.08	1.32E−26	22	0.2	0.00041
GO:0030424	Axon	712	123	0.38	1.48E−14	6	0.93	0.00044

*Note*: All KEGG pathways shown, but only top 13 pathways shown for other classifications for sake of space (sorted by phosphoproteomic FDR‐*p* value). See Tables S2 and S3 for full list of pathways identified by both studies. See Appendix S4 and Appendix S5 for list of individual genes/proteins identified and all pathways identified by each study.

### 2.6 Phosphorylation of serine 117 (S117) and serine 124 (S124), but not serine 162 (S162), are sufficient to increase PDE11A4 expression and drive PDE11A4 accumulation

**FIGURE 4:**
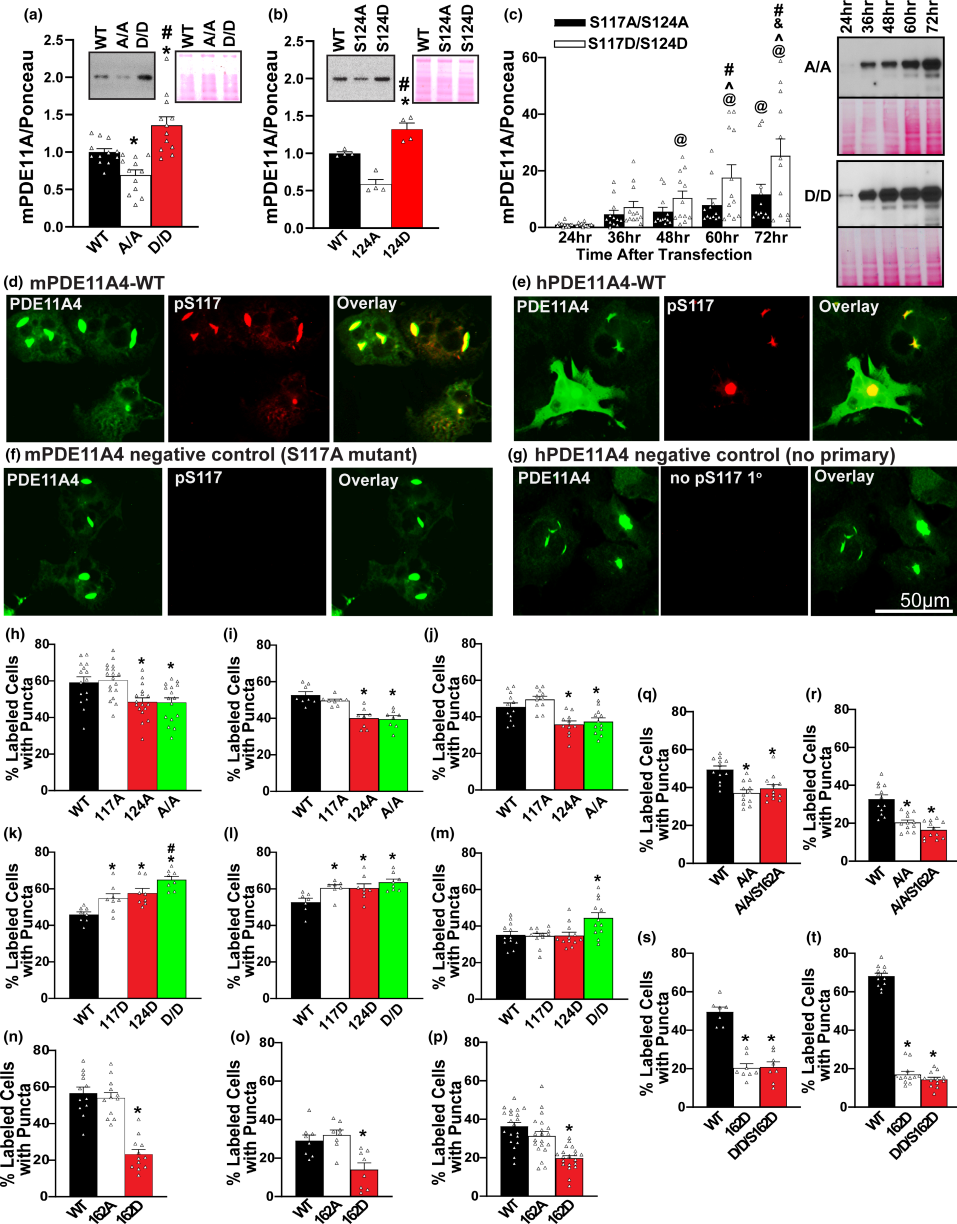
Phosphorylation of serine 117 (S117) and serine 124 (S124), but not serine 162 (S162), are sufficient to increase PDE11A4 expression and drive the accumulation of PDE11A4. Previous studies suggested PDE11A4 regulates its own protein expression levels (Hegde, Ji, et al., 2016; Smith et al., 2021). As such we determined if the increased phosphorylation of PDE11A4 at S117 and S124 observed in the aged hippocampus (Figure 1) was sufficient to cause age‐related increases in PDE11A4 protein expression and accumulation. To do so we tested the effect of phospho‐resistant (alanine, A) versus phosphomimic (aspartate, D) mutations. (a) in 2 experiments using HT‐22 cells harvested 24 h following transfection (combined data shown here), preventing phosphorylation at S117/S124 (A/A) decreased PDE11A4 protein expression while mimicking phosphorylation (D/D) increased protein expression relative to wild‐type (WT; *n* = 12 biological replicates/group; *F*[2,33] = 17.03, *p* < 0.001; post hoc: WT vs. A/A *p* = 0.011, WT vs. D/D *p* = 0.004, A/A vs. D/D *p* < 0.001. (b) This pattern was replicated in HT‐22 cells when preventing vs. mimicking phosphorylation only at S124 (S124A vs S124D, respectively; *n* = 4 biological replicates/group; *F*[2,9] = 35.94, *p* < 0.001; post hoc: WT vs. S124A *p* = 0.001, WT vs. S124D *p* = 0.005, S124A vs. S124D *p* < 0.001). (c) COS1 cells were then harvested 24–72 h following transfection and expression levels for each mutant were normalized to their own 24‐h baseline in order to determine if the differential expression noted 24 h following transfection would escalate over time. Indeed, across 2 experiments (combined data shown) the difference in expression between S117A/S124A (A/A) vs. S117D/S124D (D/D) continues to grow over time (*n* = 12 biological replicates/mutant/time; effect of mutation × time: *F*[4,44] = 4.89, *p* = 0.002; post hoc A/A 60 h vs D/D 60 h *p* = 0.003, A/A 72 h vs D/D 72 h *p* < 0.001), with D/D showing significant increases in expression over its own 24 h baseline as early as 48 hours following transfection (post hoc, D/D 24 h vs D/D 48 h *p* = 0.013) but A/A not showing a significant increase in expression over its own 24‐h baseline until 72 h after transfection (A/A 24 h vs A/A 72 h *p* = 0.011). (d) Immunocytochemistry of transfected COS1 cells with an antibody that recognizes PDE11A4‐pS117 on both mouse PDE11A4 (mPDE11A4) and (e) human PDE11A4 (hPDE11A4) shows PDE11A4‐pS117 is enriched in the accumulated pools of PDE11A4. The lack of pS117 signal in (f) the phosphoresistant mutant (S117A) and the (g) no primary antibody conditions argues the pS117 signal observed is specific. Next, we determined if preventing/mimicking phosphorylation at S117 and S124 would be sufficient to reduce/increase trafficking of mPDE11A4 into puncta. Although S117A alone had no effect, both S124A and S117A/S124A reduced the presence of PDE11A4‐filled puncta in (h) COS‐1 cells (*n* = 15–18 biological replicates/group; *F*[3,62] = 7.02, *p* < 0.001; post hoc vs WT: S124A *p* = 0.004, S117A/S124A *p* = 0.01), (i) HEK293T cells (*n* = 8 biological replicates/group; *F*[3,28] = 16.45, *p* < 0.001; post hoc: WT vs. S124A and S117A/S124A, *p* < 0.001), and (j) HT‐22 cells (*n* = 11–12 biological replicates/group; *F*[3,42] = 10.78, *p* < 0.001; post hoc vs. WT: S124A *p* = 0.004, S117A/S124A *p* = 0.005). In contrast, S117D and S124D alone or in combination increased the presence of PDE11A4‐filled puncta in (k) COS‐1 cells (*n* = 8 biological replicates/group; *F*[3,28] = 13.17, *p* < 0.001; post hoc vs. WT: S117D *p* = 0.008, S124D *p* = 0.002, S117D/S124D *p* < 0.001; post hoc vs. S117D/S124D: S117D *p* = 0.007, S124D *p* = 0.023), (l) HEK293T cells (*n* = 7–8 biological replicates/group; *F*[3,26] = 5.29, *p* = 0.006; post hoc vs WT: S117D *p* = 0.033, S124D *p* = 0.012, S117D/S124D *p* = 0.004), and (m) HT‐22 cells (*n* = 12 biological replicates/group; *F*[3,44] = 5.39, *p* = 0.003; post hoc vs S117D/S124D: WT *p* = 0.003, S117D *p* = 0.008, S124D *p* = 0.005). The ability of phosphorylation to increase the accumulation of PDE11A4 appears to be selective for S117 and S124 in that a phosphomimic mutation at S162 (S162D) reduces the accumulation of PDE11A4 in puncta in (n) COS‐1 cells (n =12 biological replicates/group; F[2,33] = 38.72, *p* < 0.001; post hoc: WT vs. S162D, *p* < 0.001), (o) HEK293T cells (*n* = 8 biological replicates/group; F[2,21] = 10.05, *p* < 0.001; post hoc: WT vs. S162D, *p* < 0.001) and (p) HT‐22 cells (*n* = 20 biological replicates/group; F[2,57] = 18.46, *p* < 0.001; post hoc: WT vs. S162D, *p* < 0.001). The ability of S117A/S124A to reduce the presence of PDE11A4‐filled puncta does not require phosphorylation of S162 in (q) COS‐1 cells, (*n* = 12 biological replicates/group; *F*[2,33] = 12.63, *p* < 0.001; post hoc: WT vs. S117A/S124A/S162A, *p* < 0.001) or (r) HT‐22 cells (*n* = 12 biological replicates/group; *F*[2,33] = 22.89, *p* < 0.001; post hoc: WT vs. S117A/S124A/S162A, *p* < 0.001). In contrast, phosphomimic mutation at S162 is able to prevent S117D/S124D‐induced accumulation of PDE11A4 in (s) COS‐1 cells (7–8 biological replicates/group; *F*[2,20] = 44.10, *p* < 0.001; post hoc: S162D vs. S117D/S124D/S162D *p* = 0.885) and (*t*) HT‐22 cells (*n* = 12 biological replicates/group; *F*[2,33] = 447.17, *p* < 0.001; post hoc: S162D vs. S117D/S124D/S162D *p* = 0.201). *vs. WT, *p* = 0.011–<0.001; #vs. other mutant(s), *p* = 0.023– <0.001; @greater than 24 h, *p* = 0.011– <0.001; ^greater than 48 h, *p* = 0.026– <0.001; ^&^greater than 60 h, *p* = 0.019. Data plotted as individual data points and mean ± SEM. Histogram stretch, brightness, and/or contrast of images adjusted for graphical clarity

Previous studies have suggested PDE11A4 regulates its own protein expression levels (Hegde, Ji, et al., 2016; Smith et al., 2021). As such we determined if the increased phosphorylation of PDE11A4 at S117 and S124 observed in the aged hippocampus (Figure 1k–n and Figure S2) was sufficient to cause aging‐like increases in PDE11A4 protein expression and accumulation. To do so, we tested the effect of phosphoresistant (alanine) versus phosphomimic mutations (aspartate, D) at these sites in vitro. We also assessed the specificity of these signals by similarly targeting S162, as it is also a PKA/PKG‐regulated serine in the N‐terminal of PDE11A4 (Kelly, 2015; Kelly, 2018b). Across multiple experiments and cell lines (i.e., COS‐1 and HT‐22), preventing phosphorylation at S117/S124 or S124 alone was sufficient to decrease PDE11A4 protein expression while mimicking phosphorylation at S117/S124 or S124 alone increased expression (Figure 4a–c).

As is observed in the aging brain, mouse and human PDE11A4 accumulates in punctate structures in vitro and PDE11A4‐pS117 is preferentially found in these puncta (Figure 4d–e). As such, we next determined if preventing/mimicking phosphorylation at S117 and S124 would be sufficient to reduce/increase trafficking of mPDE11A4 into puncta. Although S117A alone had no effect, both S124A and S117A/S124A reduced the presence of PDE11A4‐filled puncta in COS‐1, HEK293T and HT‐22 cells (Figure 4h–j). In contrast, S117D and S124D alone or in combination increased the presence of PDE11A4‐filled puncta in COS‐1 and HEK293T cells (Figure 4k–l); however, only S117D/S124D in combination was sufficient to increase puncta in HT‐22 cells (Figure 4m). The ability of phosphorylation to increase the accumulation of PDE11A4 in puncta appears to be selective for S117 and S124 in that a phosphomimic mutation at S162 (i.e., S162D) had the opposite effect of reducing the accumulation of PDE11A4 in COS‐1, HEK293T, and HT‐22 cells (Figure 4n–p). In COS‐1 and HT‐22 cells, the ability of S117A/S124A to reduce the presence of PDE11A4‐filled puncta does not require phosphorylation of S162 (Figure 4q); however, a phosphomimic mutation of S162 is able to prevent S117D/S124D‐induced accumulation of PDE11A4 (Figure 4s–t). Together, these data suggest that pS117/pS124 is a key molecular mechanism by which PDE11A4 expression and accumulation increase with age.

## 3 DISCUSSION

Here, we show that age‐related increases in hippocampal PDE11A4—but not PDE5A—are conserved across humans and rodents, as is the vulnerability of associative memories—but not recognition memories—to ARCD. Our work is consistent with previous studies showing cGMP and cAMP are decreased in the aged and demented hippocampus (rodents and humans), particularly when there is a history of TBI (Bonkale et al., 1999; Titus et al., 2013; Zhang et al., 2014). Age‐related increases in PDE11A4 appear to be driven by factors influencing both transcript and protein stability, with age‐related increases in PDE11A4 protein expression occurring in a subcellular compartment‐specific manner. Strikingly, age‐related increases in PDE11A4 in the VHIPP ectopically accumulate in filamentous structures we term ghost axons due to increased phosphorylation of PDE11A4 at S117/S124 (PDE11A4pS117/pS124; Figures 1m, n and 4a–c), reminiscent of proteinopathies caused by hyperphosphorylation of tau (c.f., Kelly, 2018a). This age‐related increase in PDE11A4‐pS117/pS124 is thought to be deleterious since STFP memory consolidation is normally associated with reduced levels (Figure 1q). Indeed, we show here that preventing age‐related increases in PDE11A via genetic deletion is sufficient to protect against ARCD of social associative memories, while mimicking age‐related overexpression of PDE11A4 in CA1 of old *Pde11a* KO mice is sufficient to cause aging‐like impairments in memory (Figure 3k) and CREB function (Figure 3n). This suggests the protective effect of PDE11A deletion in old mice is due to the absence of PDE11A4 signaling in the aged brain and a subsequent increase in VHIPP CREB activity (Smith et al., 2021), as opposed to a compensatory mechanism triggered by the chronic loss of PDE11A across the lifespan (Figure 3k–m). Notably, all of these age‐related effects appear to be relatively consistent between females (data points plotted in figures as circles) and males (data points plotted in figures as squares). RNA sequencing and phosphoproteomics analysis of the VHIPP from old *Pde11a* KO versus WT mice suggest PDE11A4 overexpression contributes to ARCD of social memories primarily via the cGMP‐PKG pathway as opposed to the cAMP‐PKA pathway and confirm downstream effects on multiple pathways associated with ARCD and Alzheimer's disease (Table 2). Together, these results suggest that increases in PDE11A expression that occur with age and TBI‐associated dementia contribute to cognitive decline.

### 3.1 Age‐related increases in PDE11A4 are driven by increased mRNA stability and phosphorylation of N‐terminal residues S117 and S124

Parsimoniously, age‐related increases in PDE11A protein expression might arise from increased transcription/translation and/or increased transcript/protein stability. With regard to PDE11A4 transcript stability, the nuclear protein p54^nrb^/NONO and the exoribonuclease XRN2 appear to be major players (Lu & Sewer, 2015). When p54^nrb^/NONO activity is compromised, XRN2 fails to bind PDE11A4 transcripts and PDE11A4 mRNA expression significantly increases, particularly in the nucleus (Lu & Sewer, 2015). Interestingly, cAMP stimulates PDE11A4 mRNA degradation via p54^nrb^/NONO (Lu & Sewer, 2015), and cellular senescence has been associated with a loss of nuclear p54^nrb^/NONO function (Huang et al., 2016). Together, these studies suggest that age‐related decreases in cAMP signaling (Kelly, 2018a) lead to increased PDE11A mRNA expression, at least in part, by virtue of the age‐related reductions in p54^nrb^/NONO‐XRN2 expression measured here (Figure 1c, d). That said, the fact that mouse DHIPP exhibits age‐related increases in PDE11A4 protein in absence of any upregulation of mRNA suggests there are additional factors at play regulating translation and/or protein stability.

Here, we identified PDE11A4‐pS117/pS124 as a key intramolecular signal that increases both protein expression and accumulation of PDE11A4. Post‐translational modifications have been shown to regulate the subcellular compartmentalization of other PDE families in vitro, including PDE2, PDE4, PDE5, and PDE10 (c.f., Baillie et al., 2019). For example, phosphorylation of PDE10A blocks palmitoylation and prevents membrane insertion (Charych et al., 2010). PDE11A4 most likely associates with the membrane by binding to a macromolecular complex as opposed to direct membrane insertion, given native gels of membrane fractions show PDE11A is localized to large macromolecular complexes and is not found as a dimer (Figure S1B). In contrast, PDE11A4 appears as a free dimer and as part of macromolecular complexes in the nuclear and cytosolic fractions. We demonstrate that pS117, pS124, and pS162 all regulate the subcellular localization of PDE11A4, with pS117/pS124 promoting the accumulation of PDE11A4 and pS162 causing dispersal. PKA and PKG phosphorylate S117 and S162 in vitro (Yuasa, Kanoh, et al., 2001) and are predicted to phosphorylate S124 (Kelly, 2015). Thus, PDE11A4 may regulate its own function through a direct feedback/feedforward loop (PDE11A4→cAMP/cGMP→PKA/PKG→PDE11A4‐pS117/pS124/pS162), as has been described for other PDE families (Baillie et al., 2019).

### 3.2 Age‐related increases in PDE11A occur in a subcellular compartment‐specific manner

We find here that age‐related increases in VHIPP PDE11A4 do not occur in a uniform/distributed manner, but rather are ectopically localized. Age‐related increases in VHIPP PDE11A4 are restricted to the membrane fraction, a compartment that normally expresses very low levels of PDE11A4 (Figure S1B; Kelly, 2018b). These findings add to a growing body of literature showing that PDE11A4 in the VHIPP membrane is the pool more readily altered by genetic and environmental factors (Hegde, Ji, et al., 2016; Pathak et al., 2017; Pilarzyk et al., 2021). We also find that age‐related increases in VHIPP PDE11A4 accumulate ectopically in filamentous structures we term “ghost axons” (Figure 2). Ectopic protein expression and accumulation is a hallmark of age‐related brain pathologies (Yanar et al., 2020). For example, in Alzheimer's disease, the hyperphosphorylation of tau within neurons leads to neurofibrillary tangle that overwhelm the neuron to the point of cell death (Moloney et al., 2021). Similarly, we show that age‐related increases in the phosphorylation of PDE11A4‐S117/S124 drives PDE11A4 to accumulate in filamentous structures that strongly resemble axonal intermediate filaments in terms of diameter and tortuosity. Despite their physical appearance, PDE11A4‐filled structures fail to colocalize with markers of axons, dendrites, cilia, or glia (Figure 2), even when injection of recombinant PDE11A4 into CA1 of a *Pde11a* KO mouse generates PDE11A4‐filled structures in brain regions distal to the injection site (Figure 2d). We speculate that PDE11A4 accumulation within the axon overwhelms the system, occluding the co‐localization of other axonal proteins or causing degeneration of the surrounding structure. Thus, we name these PDE11A4‐filled structures “ghost axons” in homage to tau “ghost tangles” (Moloney et al., 2021; Uchihara, 2014). The ectopic nature of the age‐related accumulation in PDE11A4 raises the interesting possibility that its deleterious effects on cognition are not related to a simple gain of PDE11A4 catalytic activity, but rather may reflect PDE11A4 interfering with the binding/localization of another protein (i.e., a loss of function). Notably, mRNA expression and phosphoproteomic changes in VHIPP of old *Pde11a* WT vs KO mice were enriched for pathways related to Alzheimer's disease, axon guidance, regulation of transport, chemical synaptic transmission, as well as the axon, synapse, presynapse, and synaptic vesicle (Table 2). It will be of interest to future studies to determine whether the lack of PDE11A4 co‐localization with axon markers may reflect occlusion or axonal degeneration.

Although the age‐related increases in PDE11A4 protein expression are clearly deleterious to aLTM, as demonstrated by the protective phenotype observed in *Pde11a* KO mice, it is possible that the accumulation of PDE11A4 in ghost axons reflects a protective mechanism attempting to sequester and/or inactivate the excess protein. Such sequestration is reported to occur with select PDE4A isoforms when they are both catalytically inhibited and conformationally altered (Christian et al., 2010). If so, it would be important for a PDE11A4‐targed therapeutic to not only reduce the accumulation of PDE11A4, but also to promote its clearance from the system. Disruption of PDE11A4 homodimerization may then represent a refined mechanism for therapeutically targeting age‐related proteinopathies in PDE11A4 since in vitro studies show that disrupting PDE11A4 homodimerization both reduces the punctate accumulation of PDE11A4 and promotes proteolytic degradation of membrane‐associated PDE11A4 (Pathak et al., 2017).

### 3.3 Age‐related increases in PDE11A4 are more deleterious in combination with brain injury

In elderly humans with a history of TBI, hippocampal PDE11A mRNA expression is significantly higher in those that developed Alzheimer's disease or a related dementia versus those that did not (Figure 1e). This is particularly interesting given that pathway analysis of our previous proteomics study of VHIPP from *Pde11a* KO versus WT mice (Pilarzyk et al., 2021), along with pathway analyses of the RNA sequencing and phosphoproteomics studies reported herein (Table 2), identified the Alzheimer's disease pathway. Together, our findings complement those of Qin and colleagues who associated rare PDE11A mutations with early‐onset Alzheimer's Disease (Qin et al., 2021). The TBI‐associated dementia‐related increase in PDE expression is unique to PDE11A in that PDE2A, PDE5A, and PDE10A expression remained unchanged. That said, we do acknowledge that inhibition and/or genetic deletion of several PDE families has shown efficacy in models of cognitive impairment (Devan et al., 2014; Orejana et al., 2012; Palmeri et al., 2013), suggesting a PDE itself does not have to change with age/disease in order for its targeting to elicit therapeutic effects. The fact that age‐related increases in PDE11A are exacerbated in elderly patients with TBI‐associated dementia is consistent with reports in humans that TBI worsens age‐related decreases in hippocampal cAMP levels (Titus et al., 2013) as well as ARCD (Crane et al., 2016). Further, TBI accelerates the onset of dementia (Barnes et al., 2018; Fann et al., 2018; Nordström & Nordström, 2018), Alzheimer's disease (Schaffert et al., 2018), and Parkinson's disease (Gardner et al., 2018). Even surgical injury in aged humans contributes to post‐operative cognitive dysfunction and increases risk of developing dementia (Alam et al., 2018). This raises the interesting possibility that brain injury itself causes an aging‐like dysfunction of PDE11A4, leading those with higher baseline levels or larger age‐related increases in PDE11A4 to transition from simple ARCD to dementia. Alternatively, age‐related dysfunction of PDE11A4 may interact with some other pathology induced by injury, such as inflammation (Pathak et al., 2017; Pilarzyk et al., 2021; Porcher et al., 2021) or altered proteostasis (c.f., Saikumar & Bonini, 2021). Indeed, whereas unsurgerized aged mice with elevated PDE11A4 expression demonstrate intact remote rLTM for SOR (Figure 3f), hippocampally‐surgerized aged mice with elevated PDE11A4 expression show deficits (Figure 3l). Importantly, all mice show intact remote rLTM for NSOR, pointing to a hippocampus‐specific deficit. It will be of interest to future studies to determine how PDE11A4 may play a role in the pathological consequence of brain injury across the lifespan.

### 3.4 Deletion of PDE11A triggers changes in pathways related to ARCD and dementia

Pathway analyses from an RNA sequencing and confirmatory phosphoproteomics study comparing VHIPP from old *Pde11a* WT vs KO mice identified multiple mechanisms by which PDE11A4 may contribute to ARCD and/or dementia (Table 2). Of note, the cGMP‐PKG signaling was identified but not the cAMP‐PKA pathway, which is consistent with literature reporting age‐related decreases in cGMP but not cAMP in the hippocampus (Chalimoniuk & Strosznajder, 1998; Kelly, 2018a; Vallebuona & Raiteri, 1995). Indeed, several studies show nootropic effects of cGMP‐elevating compounding in the context of ARCD and neurodegenerative diseases (Cossenza et al., 2014; Fiorito et al., 2013; Fiorito et al., 2017; Kleppisch & Feil, 2009; Palmeri et al., 2013; Puzzo et al., 2009). In addition to regulating learning and memory, the cGMP‐PKG pathway is also required for regulating sleep and circadian rhythms (Agostino et al., 2007; Langmesser et al., 2009; Ribeiro & Kapas, 2005). Sleep quality reduces with age (Bliwise, 1993; Cajochen et al., 2006; Ohayon et al., 2004) and age‐related diseases (Brayet et al., 2016; Hita‐Yanez et al., 2012; Petit et al., 2004), and sleep disruption appears to negatively impact cognitive function more severely in aged versus young adults (Liu et al., 2019; Mendelson & Bergmann, 2000). As such, we find it highly interesting that circadian entrainment was one of the top PDE11A‐regulated pathways identified in this study, particularly when a PDE11A mutation (Y727C) has been associated with sleep quality in humans (Jones et al., 2019). It will be of interest to future studies to determine if the ability of PDE11A deletion to prevent age‐related cognitive decline of social aLTMs is a direct consequence of ameliorating age‐related deficits in circadian rhythms and/or quality of sleep.

### 3.5 Conclusions

In summary, proteinopathies in PDE11A4 develop with age, and age‐related increases in PDE11A4 expression that are conserved across species are sufficient to cause ARCD of social associative memories. PDE11A4 is unique because in brain it is the only PDE to be expressed preferentially in the hippocampal formation, a structure critical for associative memories (Kelly et al., 2010; Kelly et al., 2014). This—along with the fact that PDE11A is a highly druggable enzyme (Kelly, 2015)—makes PDE11A a very attractive therapeutic target because it stands to selectively restore aberrant cyclic nucleotide signaling in a brain region affected by age‐related decline without directly affecting signaling in other brain regions or peripheral organs that might lead to unwanted side effects (Kelly, 2015). By gaining a further understanding of the intramolecular signals controlling PDE11A4 subcellular localization (e.g., N‐terminal phosphorylation, homodimerization), we hope to develop even more sophisticated therapeutics that can target disease‐specific PDE11A4 proteinopathies.

## 4 EXPERIMENTAL PROCEDURES

### 4.1 Mouse subjects

Mating trios (1 male × 2 females) of C57BL/6J and BALB/cJ mice were originally obtained from Jackson laboratory and then bred onsite either at the University of South Carolina School of Medicine or the University of Maryland School of Medicine. Old 129S6/SvEv were originally obtained from Taconic at approximately 2 months of age and were then aged onsite at the University of South Carolina School of Medicine, with young mice ordered from Taconic 2 weeks prior to tissue harvest for comparison. As previously published (e.g., Kelly et al., 2010; Pilarzyk et al., 2019; Smith et al., 2021), the *Pde11a* mouse line was originally obtained from Deltagen (San Mateo, CA) and then maintained on a mixed C57BL6 background (99.8% multiple C57BL/6 substrains and 0.2% 129P2/OlaHsd; Smith et al., 2021) as well as a 98.8% BALB/cJ background (Smith et al., 2021). Note that knockouts on the BALB/cJ background were only used as negative controls in immunofluorescent staining experiments using PDE11A antibodies; no behavior was conducted in the BALB/cJ line. See figure legends for specific *n*'s/sex/group/experiment. For experiments herein, young was defined as 2–6 months old, middle aged was defined as 10–15 months, and old was defined as 18–22 months. Animals were housed on a 12:12 light:dark cycle (experiments conducted during lights on) and allowed ad lib access to food and water, except when undergoing STFP. Experiments were conducted in accordance with the National Institutes of Health Guide for the Care and Use of Laboratory Animals (Pub 85–23, revised 1996) and were fully approved by the Institutional Animal Care and Use Committee of the University of South Carolina and the University of Maryland, Baltimore. For more detailed information, see the Appendix S1.

### 4.2 Assays

Studies were largely conducted as per previously published methods (Farmer et al., 2020; Hegde, Capell, et al., 2016; Hegde, Ji, et al., 2016; Kelly, 2014; Kelly et al., 2014; Patel et al., 2018; Pathak et al., 2017; Pilarzyk et al., 2019; Pilarzyk et al., 2021; Porcher et al., 2021; Smith et al., 2021). In brief, age‐related changes in human mRNA were assessed by mining RNA sequencing data from the 2014 Allen Institute for Brain Science Brainspan database and the Aging, Dementia, and Traumatic Brain Injury (TBI) database, as we previously published (Farmer et al., 2020; Patel et al., 2018). In situ hybridization was conducted as previously described (Kelly, 2014; Kelly et al., 2014) using an ^35^S–labeled antisense probe (5′‐ccaccagttcctgttttccttttcgcatcaagtaatc‐3′), the sense correlate of which yielded no signal. As previously described, biochemical fractionation (Patel et al., 2018; Pathak et al., 2017; Porcher et al., 2021), immunofluorescence (IF)/immunohistochemistry (IHC) (Hegde, Capell, et al., 2016), social transmission of food preference (Hegde, Capell, et al., 2016; Pilarzyk et al., 2019), odor recognition (Hegde, Capell, et al., 2016; Pilarzyk et al., 2019), plasmid generation (Pathak et al., 2017), cell culture and transfections (Pathak et al., 2017), phosphodiesterase activity assays (Smith et al., 2021), stereotaxic surgery (Pilarzyk et al., 2019), and RNA sequencing (Hegde, Ji, et al., 2016) were conducted. 2‐dimensional difference in gel electrophoresis (2‐D DIGE) and mass spectroscopy (MS) for the phosphoproteomic study was conducted by Applied Biomics (Hayward, CA; https://www.appliedbiomics.com/2d‐dige/phosphoproteomics/), similarly to a study previously described (Pilarzyk et al., 2021) with some modification. See the Appendix S1 for a detailed description of each Method.

### 4.3 Data analysis

Data were collected blind to treatment and experiments were designed to counterbalance technical variables across biological variables. Data points greater than two standard deviations away from the mean were removed as outliers prior to analyses, as previously described (e.g., Kelly et al., 2009; Kelly et al., 2014; Pathak et al., 2015; Pilarzyk et al., 2019). Outliers removed/total *n*: Figure 1o, 1/24; Figure 3a, 4/89; Figure 3b, 3/73; Figure 3c, 7/110; Figure 3i, 4/73; Figure 3l, 2/43; Figure 3m, 5/4; Figure 4l, 2/48; Figure S6F, 2/38; Figure S7C, 5/108. Data were analyzed for effect of genotype, age, behavioral parameter (e.g., bead and food), and sex for experiments with greater than 6/sex/genotype (Hegde, Capell, et al., 2016; Kelly et al., 2010; Kelly et al., 2014; Pilarzyk et al., 2019)). Where datasets met assumptions of normality (Shapiro–Wilk test) and equal variance (Levene's test), the following parametric statistical analyses were run on Sigmaplot 11.2 (San Jose, CA, USA): ANOVA (F), Student's *t*‐test (*t*), one‐sample *t*‐test (*t*; Table 1). In the case of one‐sample t‐tests (determining whether or not a given group demonstrated memory), a false‐rate discovery (FDR) correction was applied to all *p*‐values within an experiment to mitigate the risk of Type I error associated with multiple comparisons. Where normality and/or equal variance assumptions failed, the following non‐parametrical statistical analyses were run: Kruskal–Wallis ANOVA (H), Mann–Whitney rank sum test (T), or Wilcoxon Signed Rank Test (Z). Repeated measures analyses were used where appropriate (e.g., in analysis of behavior across multiple trials). Post hoc analyses were performed according to the Student–Newman–Keuls or Dunn's method and significance was defined as *p* < 0.05. Source data can be found in Appendix S2 and original uncropped images can be found in Appendix S3.

## AUTHOR CONTRIBUTIONS

KP, LP, WRC, SDB, JD, NG, and SP collected/analyzed data and wrote parts of the manuscript. JLF created essential reagents and wrote part of the manuscript. MPK conceptualized the study, collected/analyzed data, and wrote/edited the manuscript. All authors read and approved the final manuscript.

## CONFLICT OF INTEREST

The authors report no conflicts of interest.

## Supporting information


**Appendix S1**: Supplemental Experimental Procedures and Figures with Legends.

**Appendix S2**: Source Data for Figures

**Appendix S3**: Uncropped images.

**Appendix S4**: RNA sequencing data.

**Appendix S5**: Phosphoproteomics data.

**Table S1**: Amount of food (grams) eaten during STFP tests or amount of time spent invenstigating (seconds) during SOR/NSOR tests by young (Y) and old (O) wild‐type (WT) and knockout(KO) mice during the short‐term memory (STM), 24‐hour long‐term memory (LTM), and 7‐day LTM tests (Data expressed as mean ±SEM).

**Table S2**: GO Processes Pathways identified as significantly enriched for both mRNA changes and phosphoproteomic changes in VHIPP old *Pde11a* KO vs WT

**Table S3**: GO Component Pathways identified as significantly enriched for both mRNA changes and phosphoproteomic changes in VHIPP old *Pde11a* KO vs WT mice.

## Data Availability

All source data and uncropped original images contained in this manuscript can be found as [Link], [Link].
