# Supplementary material for: Conserved age‐related increases in hippocampal PDE11A4 cause unexpected proteinopathies and cognitive decline of social associative memories
Source: Aging Cell. 2022 Sep 8;21(10):e13687. doi: 10.1111/acel.13687 (PMC9577960; doi:10.1111/acel.13687)
Supplement: Supplementary file 1 — Appendix S1: Supplemental Experimental Procedures and Figures with Legends. [file ACEL-21-e13687-s001.pdf]

Supplemental Experimental Procedures and Figures for Pilarzyk, Porcher, Capell et al.

SUPPLEMENTAL EXPERIMENTAL PROCEDURES

Human expression data. To assess age-related changes in PDE11A, PDE5A, XRN2, and NONO mRNA, RNA sequencing data from human hippocampi were mined from the 2014 Allen Institute for Brain Science Brainspan database, as we previously published [1, 2]. Data were downloaded by navigating to the following website and using the enzyme names listed above in the search box: https://www.brainspan.org/rnaseq/search/index.html. Methods used for this RNA sequencing study have been previously published [3] and can be found here: http://help.brain-map.org/display/devhumanbrain/Documentation. Data were also mined from the 2016 Allen Institute for Brain Science Aging, Dementia, and traumatic brain injury (TBI) study, a study conducted in a cohort of adults 75+ years old. Data for the entire study were downloaded from http://aging.brain-map.org/download/index, and the methods for this RNA sequencing study can be found here: http://help.brain-map.org/display/aging/Documentation. Subjects from the Brainspan database were grouped into the following age categories based on our previous studies [1, 2]: prenatal, childhood (2 months-17 years old), and adulthood (18-40 years old). Subjects from the Aging, Dementia, and TBI database were divided into four groups based on whether they had a history of TBI and/or had been diagnosed with Alzheimer's disease or a related dementia (ADRD).

Mouse Subjects. Mating trios (1 male x 2 females) of C57BL/6J and BALB/cJ mice were originally obtained from Jackson laboratory and then bred onsite either at the University of South Carolina School of Medicine or the University of Maryland School of Medicine. Old 129S6/SvEv were originally obtained from Taconic at approximately 2 months of age and were then aged onsite at the University of South Carolina School of Medicine, with young mice ordered from Taconic 2 weeks prior to tissue harvest for comparison. As previously published (e.g., [4-6]), the *Pde11a* mouse line was originally obtained from Deltagen (San Mateo,

CA) and then maintained on a mixed C57BL6 background (99.8% multiple C57BL/6 substrains and 0.2% 129P2/OlaHsd [6]) as well as a 98.8% BALB/cJ background [6]. Note that knockouts on the BALB/cJ background were only used as negative controls in immunofluorescent staining experiments using PDE11A antibodies; no behavior was conducted in the BALB/cJ line. *Pde11a* mice were bred onsite in heterozygous (HT) x HT trio matings, with 3-5 same-sex wild-type (WT), heterozygous (HT), and knockout (KO) littermates (defined as any mice born at the same time to either dam) weaned together. As previously described [6, 7], litter effects are unlikely driving findings described herein as each dataset reflects either 1) a combination of 2+ subcohorts born and tested at different times or 2) is fully replicated in a separate cohort of mice. Further, any given litter of *Pde11a* mice typically contributes n=1-2 mice/genotype and, at most, parents contribute 2 litters to a cohort (i.e., a total of 2-4 mice/genotype). Males and females were used throughout each experiment, but not necessarily in sufficient numbers to power an analysis of sex effects (see figure legends for specific *n*'s/sex/group/experiment). For experiments herein, young was defined as 2-6 months old, middle aged was defined as 10-15 months, and old was defined as 18-22 months. As previously described [7], we do not conduct gross pathology, however, mice are generally healthy at the time of testing. Mice are routinely assessed by husbandry, veterinary and laboratory staff and mice with palpable tumors >1 cm, lethargy, altered gait, signs of malnutrition or dehydration are removed from study and euthanized. Mice demonstrated no evidence of a striking anatomical abnormality of the brain upon dissection (e.g., a pituitary tumor) [7]. Animals were housed on a 12:12 light:dark cycle and allowed ad lib access to food and water. Experiments were conducted in accordance with the National Institutes of Health Guide for the Care and Use of Laboratory Animals (Pub 85-23, revised 1996) and were fully approved by the Institutional Animal Care and Use Committee of the University of South Carolina and the University of Maryland, Baltimore.

Tissue Collection. As previously described [7], mice were euthanized during the light cycle via cervical dislocation. Mouse brains were harvested fresh, dissected on wet ice, and stored at -80 °C for further processing.

In situ hybridization. Brains were stored at −80 °C until being cryosectioned at −19 to -17 °C in the sagittal plane at 20 μm, with sections thaw mounted on superfrost slides. As previously described [8, 9], slides were pretreated in a series of 4% paraformaldehyde, 1M Triethanolamine/0.9% NaCl, ethanol (70-100%), chloroform, ethanol (100-70%), and sterile water and were then allowed to air dry before storing at -80 °C. *In situ* hybridization for PDE11A4 was also conducted as previously described [8, 9] using an S35-labelled antisense probe (5′-ccaccagttcctgttttcctttcgcatcaagtaatc-3′). The probe was end labeled with ^{35}S-dATPα using terminal deoxytransferase and extracted using Illustra Microspin G-25 columns (GE Healthcare, North America). Each slide was then hybridized with 600,000 cpms of labeled probe in 125 μl of hybridization buffer and slides incubated overnight in a humid chamber warmed to 37° C. The following day, slides were washed in a series of saline-sodium citrate (SSC) buffers with 0.1 M dithiothreitol (2X SSC room temperature then 0.2X SSC at 55° C). Slides were dehydrated in 70% ethanol and allowed to air dry. Once dry, slides were exposed to Kodak BioMax MR film for 14 days, generating optical densities that were within the linear range of the film. Note, antisense probes yielded consistent expression patterns (see Figure 1) while sense probes yielded no signal as previously published [8, 9].

Biochemical Fractionation and Western Blotting. Biochemical fractionation was performed as previously described [2, 7, 10] (see Figure S1A for validation of our fractionation protocol). In short, cells or tissue were sonicated in ice cold fractionation buffer (20 mM Tris-HCl, pH 7.5; 2 mM MgCl2; Thermo Pierce Scientific phosphatase tablet #A32959 and protease inhibitor 3 #P0044). For those samples processed by biochemical fractionation, successive rounds of differential centrifugation were conducted. Following a low-speed spin (1000 x g) to isolate the nuclear fraction pellet, the supernatant was transferred to a new tube and subjected to a high-speed spin (89,000 x g) to isolate the cytosolic proteins in the supernatant and the membrane proteins in the pellet. The pellet was then resuspended in fractionation buffer with 0.5% Triton-X 100 to solubilize the protein, with a

subsequent high-speed spin (60,000 x g) to isolate the supernatant containing soluble membrane proteins. Note that the nuclear and membrane pellets were "rinsed" prior to the resuspensions described above. That is, these pellets were resuspended in the previous buffer and respun with resulting supernatant discarded. Protein concentrations were determined by DC Protein Assay kit (Bio-Rad; Hercules, CA, USA) according to manufacturer protocol, and were subsequently equalized across samples. Samples were stored at -80°C until further processing. For western blotting, 10μg of total protein for cells, 30μg of total protein for tissue, or 20μg for membrane, cytosolic, or nuclear fractions from tissue were loaded onto 12% NuPAGE Bis-Tris gels (Invitrogen, Waltham MA) and electrophoresed for one hour at 180 volts. Previously verified hippocampal tissue from a PDE11A KO mouse was included in all PDE11A4 blots as a negative control. Protein was transferred onto a 0.45μm nitrocellulose membrane for two hours at 100 mA. Membranes were then washed twice in tris-buffered saline with 0.1% tween20 (TBS-T) before staining with Ponceau S to determine total protein loading. Note, Ponceau S was chosen over a housekeeping gene as a loading control based on the best-practice statement of the Journal of Biological Chemistry [11]. Images of the stained membranes were collected to later quantify the optical density of the total protein stain (i.e., spanning ~200kDa to 10kDa), and then the membranes were rinsed in TBS-T to remove the stain. Blots to be probed with the #1 antibody were blocked in 5% milk while all others were blocked in Superblock Blocking Buffer (ThermoFisher, Cat#37515), each with 0.1% Tween 20. Overnight primary antibody incubation at 4°C was completed for the following targets: PDE11A (Aves custom PDE11#1 at 1:10,000; Fabgennix PD11A-112 at 1:500), CREB (Cell Signaling #4820 at 1:10,000) as well as synaptophysin (BD Transduction #611880 at 1:200,000), histone 3 (Millipore 05-928 at 1:5000) and p-AKT (Cell Signaling 9271S at 1:1000). The next day, membranes were washed four times with TBS-T for ten minutes each. Secondary antibody incubation was completed for one hour at room temperature. The following Jackson Immunoresearch secondary antibodies were used: anti-rabbit (Jackson Immunoresearch, 111-035-144; 1:10 000), anti-chicken (Jackson Immunoresearch, 103-035-155; 1:40 000), and anti-mouse (Jackson Immunoresearch, 115-035-003; 1:10 000). Three final washes in TBS-T were done for fifteen

minutes each. The membranes were immersed in SuperSignal West Pico Chemilumiscent Substrate (ThermoScientific, Waltham MA) and then exposed to film. Multiple film exposures were taken to ensure signals were within the linear range, and Ponceau stain and western blot optical densities were quantified using Image J. To account for gel-gel variances in differences in film exposure, how well antibodies incorporated onto each membrane, how well chemiluminescence reacted between blots, etc., Western blot data were normalized to a control condition (e.g. EmGFP-mPDE11A4^{WT}) on each blot, as previously described (e.g., [2, 7, 8].

Immunofluorescence (IF)/Immunohistochemistry (IHC). For most experiments, fresh-harvested, flash-frozen brains were embedded in matrix, cryosectioned in the sagittal plane at 20 μM, and thaw-mounted two sections per slide. One exception was that perfused tissue was sectioned with a vibratome at 50 μm for labeling using the AC3 antibodies described below. As previously described [12], 4% paraformaldehyde in PBS for 20 minute was used to fix frozen tissue. After fixation, the tissue was washed three times for ten minutes each in PBS with bovine serum albumin and triton X-100 (PBT). To reduce non-specific labeling, antibodies hosted in rodent species were pretreated with anti-Mouse FabFragments (0.15mg/ml; Jackson Immunoresearch # 715-007-003) in PBS for 2 hours. The FabFragments were then washed off by 3x10 minute washes in PBT prior to adding primary antibody. Overnight incubation with antibodies to the following targets was done at 4°C. Antibodies were mixed in PBT at optimized dilutions: PDE11A (FabGennix PD11-112 at 1:100; Aves custom PDE11A4 #1 at 1:10,000; Fabgennix PPD11A-140AP at 1:1000; Fabgennix PPD11A-150AP at 1:500), NF-L (Cell Signaling #2837 at 1:10,000), β-tubulin (Covance PRB-435P at 1:100,000), MBP (Aves MBP at 1:5000), MAP2 (Neuromics CH22103 at 1:2000), AC3 (PhosphoSolutions 85-AC3 at 1:500 or Thermofisher Scientific PA5-35382 at 1:500 produced equivalent results), α-internexin (Phosphosolutions 100-AIN at 1:5000), peripherin (Invitrogen PA1-10018, 1:1000), Ankryin-G (UC Davis/NIH Neuromab Facility Incorporated 75-146 at 1:100), lectin (Sigma, L1516 at 1:500), IBA-1 (Wako 019-19741 at 1:1000), and GFAP (Cell Signaling 12389P at 1:10,000).

The following day, sections were washed 4x in PBT and incubated for 90 min in secondary antibody, with co-labeling studies prioritizing secondaries recognizing the PDE11A antibodies first (Alexafluor 488 AffiniPure Donkey Anti-Chicken, 1:1000, Jackson Immunoresearch #703-545-155 or Alexafluor 488 AffiniPure Donkey Anti-Rabbit, 1:1000, Jackson Immunoresearch # 711-545-152), followed by 3X10 min PBT washes, then the secondaries recognizing the co-labeling marker antibody (Alexafluor 594 AffiniPure species-specific, 1:1000, Jackson Immunoresearch). After the secondary antibody incubation(s), slides were washed in PBT three times for ten minutes each. The slides were then rinsed ten times in PBS. For IF, slides were then mounted with DAPI fluoromount (Southern Biotech, #0100-20). In the case of immunohistochemistry, slides were processed as previously described [13]. Slides were treated with ABC solution (Vector Laboratories, PK-6100) prepared in PBT for 90 minutes. More specifically, after washing 3X10 washes with PBS sections were subjected to a DAB reaction (Vector Laboratories, SK-4100) until fully developed. Slides were placed in PBS once the reaction was complete. After being dried at room temperature, slides were mounted with Permount mounting media (Fisher, SP15-100) and a coverslip. Slides were kept covered and refrigerated until viewed and imaged. For quantification of PDE11A-filled structures and some co-labeling studies, images were taken using a CX9000 camera and Neurolucida imaging software (MBF Bioscience; Williston VT) mounted on a Nikon Eclipse 80i bright-field microscope that is equipped with a dry 20X objective. For co-labeling studies, images were captured using a LASX software and a Leica SP8 confocal microscope with oil immersion objectives 20X-63X. The number of structures was quantified by an experimenter blind to treatment.

Social Transmission of Food Preference. As previously described [5, 12], mice were food restricted such that access to food was limited to 1 hour per day for 2 days prior to the start of training. 24 hours prior to training, all mice were first habituated to a powdered version of their regular food (i.e., their standard Envigo Teklad 8904 food pellets were pulverized using a standard kitchen blender) by being given access to a jar full of it in a clean home cage. The next day, a mouse designated as the “demonstrator” was moved to a clean home cage alone

and fed a powdered version of their food laced with a household spice for one hour (e.g. McCormick brand basil vs. ginger, thyme vs turmeric, mint vs cardamom, orange vs anise, or basil vs thyme available in any grocery store). Note that some cohorts of mice were trained and tested at a given retrieval time point with one spice combination and then trained and tested at a different retrieval time point with a different spice combination in order to reduce the total number of mice used herein. For example, data shown in Figure S6 are all from the same cohort of middle-aged mice that were trained/tested at different times, with a different spice combination used at each retrieval time point. We have extensively detailed elsewhere that such an approach does not confound interpretation of our data [5]. The demonstrator is then returned to the home cage and their "observer" cage mates interact with the demonstrator for 15 minutes, during which time the observers make an association between the non-social odor (household spice) and the social pheromones in the breath of the demonstrator. 15 minutes, 24 hours, or 7 days after training, the observer mice are then tested for 60 minutes by being placed in a clean home cage by themselves and given access to 2 powdered foods-- one laced with the spice that the demonstrator was given and the other a novel spice. The amount of food eaten and the amount of time spent eating the food (scored for 1 minute out of every 10 minutes) was calculated by an experimenter blind to group. All mice met minimum inclusion criteria of eating at least 0.25 gm of food and being observed to eat for at least 5 seconds. Memory formation/retrieval is defined as the observer mice eating more of the food laced with the familiar spice that was on their demonstrator's breath versus the novel spiced food (preference ratio: familiar-novel/familiar+novel).

Odor Recognition. As previously described [5, 12], 1'' round wooden beads (Woodworks) were placed in the subjects' home cages for at least 7 days prior to testing to allow for habituation and saturation of the beads with social odors. Training involved a habituation trial with 3 beads from the subject's home cage, followed by 2 subsequent trials that included 2 home-cage beads and 1 novel-scented bead. For social odor recognition (SOR), the novel-scented bead came from a cage of sex-matched mice of a different strain (e.g., C57BL/6J Jax

#000664, BALB/cJ Jax #000651, 129S6/ SvEv Taconic #129SVE, C3H/HeJ #000659, A/J #000646). For non-social odor recognition (NSOR), the novel-scented bead came from a baggie of bedding saturated with a household spice (e.g., basil, ginger, marjoram, cumin, etc.). Memory was then tested 1 hour (short-term memory, STM), 24 hours (recent long-term memory, LTM) or 7 days later (remote LTM). For SOR testing, mice were presented with one home cage bead, one bead from the familiar donor strain used during training, and one bead from a second novel donor strain. For NSOR, mice were presented with 1 bead scented with the training spice and 1 bead with a novel household spice. The donor/spice assigned as "novel" within a given set of scents was counterbalanced across subjects. Mice were allowed to investigate the beads for 2 minutes and time spent investigating beads was manually scored by an experimenter blind to the conditions. All mice met minimum inclusion criteria of spending at least 3 seconds sniffing. Memory is defined as spending more time investigating the novel vs familiar scent (preference ratio: novel-familiar/novel+familiar).

Plasmid generation. Plasmids were generated as previously described [10]. Briefly, Genscript (Piscataway, NJ) generated constructs expressing either EmGFP alone containing an A206Y mutation to prevent EmGFP dimerization [14] or the mouse *Pde11a4* (NM_001081033) sequence fused at the N-terminal with EmGFP. These constructs were initially generated on a pUC57 backbone and then subcloned into a pcDNA3.1+ mammalian expression vector (Life Technologies; Walthan, MA). *Pde11a4* mutations (i.e., S117A, S124A, S117D, S124D, S162A, S162D) were introduced using the QuickChange procedure and products as per manufacturer's instructions (Agilent Technologies; Santa Clara, CA). Oligonucleotide primers used in the QuickChange procedure were generated by Integrated DNA Technologies (Coralville, IA) and mutated DNA sequences were subsequently verified by Functional Biosciences (Madison, WI).

Cell culture and transfections. Cell culture and transfection were conducted as previously described [10], with cultures periodically confirmed to be mycoplasma negative by the laboratory of Dr. Mythreye Karthikeyan at

the University of South Carolina. Briefly, COS-1 (male monkey fibroblast cell line as per web.expasy.org), HEK293T (female human embryonic kidney cell line as per web.expasy.org), and HT-22 cells (undefined-sex mouse hippocampal cell line) were grown in t-75 flasks containing Dulbecco's Modified Eagle Medium (DMEM) (GIBCO; Gaithersburg, MD, USA) with added 10% fetal bovine serum (FBS) (GE Healthcare Life Sciences; Logan, UT, USA) and 1% Penicillin/Streptomycin (P/S) (Mediatech, a Corning subsidary; Manassas, VA, USA). Cells were incubated at 37°C/5% CO_2 and passaged once 70-90% confluent, using TrypLE Express (GIBCO; Gaithersburg, MD,USA) as a dissociation agent. One day prior to transfection, cells were plated in either 24-well plates or 6-well plates with cover slips for imaging experiments or 100 mm dishes for biochemistry, all of which contained DMEM+FBS+P/S. The following day, media was replaced with Optimem (GIBCO), and cells were transfected with the pcDNA3.1+ vectors using Lipofectamine 2000 (Invitrogen; Carlsbad, CA) according to manufacturer protocol using a ratio of 3.75μg DNA plus 10uL lipofectamine per 10 mLs of media. ~19 hours post-transfection (PT), Optimem + Lipofectamine was replaced with DMEM+FBS+P/S. For most studies, cells were allowed to grow for five hours before sample processing (e.g. fixation for imaging, sonication for Western blot, etc.) and, thus, were harvested ~24 hours following transfection. The one exception was in the case of the COS1 experiment that tracked protein expression changes between the S117A/S124A versus S117D/S124D mutants from 24 hours to 72 hours following transfection. For imaging experiments, cells were fixed with 4% paraformaldehyde in PBS for fifteen minutes and then kept in PBS for imaging using a CoolSNAP EZ CCD camera (Photometrics, Tuscon AZ) that was mounted on an inverted Leica (Wetzlar DE) DMIL microscope with a Fluotar 10X/0.3 ∞/ 1.2 objective. The images were captured using NIS-Elements BR-2.3 (Nikon,Tokyo Japan) software. Images were then systematically hand scored by an experimenter blind to condition. This systematic process involved placing a 4x5 grid over the image in powerpoint to help keep track of position and using the same digital zoom for all images within an experiment, typically between 250% to 400%, depending on the experimenter. The cells within each box of the grid were then classified as exhibiting cytosolic-only labeling versus punctate labeling, and data were then entered for that grid into a grid-shaped

template. Once all grids for an image were evaluated, data were typed into an .xls template that calculated the total number of cells that were labelled and the percent of labeled cells that exhibited cytosolic versus punctate labeling (data graphed as the latter).

PDE assay. As previously described [15], cells were harvested in buffer containing 20 mM Tris-HCl and 10 mM $MgCl_2$ and kept on ice until ready to use. [3H]cGMP (Perkin Elmer, NET337) or [3H]cAMP (Perkin Elmer, NET275) were prepared at 4x 10^4 disintegrations per minute in the same solution in which the cells were harvested. In a tube containing 50 uL of cell lysates, 50 uL of [3H]cGMP or [3H]cAMP was added and the reaction took place for 10 minutes. The reaction was then quenched with 0.1 M HCl and neutralized with 0.1 M Tris base. To convert the product of PDE reaction (5′AMP or 5′GMP) to the corresponding nucleoside and phosphate, 3.75 mg/mL of snake venom (Crotalus atrox, Sigma V-7000) was added to the samples for 10 minutes at 37°C. Samples were then loaded into 5′polystyrene chromatography columns with coarse filters (Evergreen, 208-3383-060) containing DEAE Sephadex A-25 resin (VWR, 95055-928). Once the sample completely flowed through the column, the columns were eluted with 4 washes of low salt buffer containing 20 mM Tris-HCl and 0.1% sodium azide. Once all 4 washes were complete, 4 mL of Ultima Gold XR scintillation fluid (Fisher, 50-905-0519) was added directly to the samples and vortexed vigorously. The samples were then run through a scintillation counter (Beckman LS 6000) to obtain the counts per minute. Data were then normalized to the total amount of protein per sample.

Stereotaxic Surgery. As previously described [5], the NeuroStar motorized stereotaxic and drill and injection robot (Tubingen, Germany) was used to conduct stereotaxic surgery. Mice were anesthetized with a mixture of oxygen and isoflurane at an induction rate of 3%. Upon initial administration, lack of reflexes was verified and isoflurane administration was maintained at 1.5-2%. A robotic drill was used to drill small holes at the following coordinates relative to Bregma (dCA1 AP, 1.7, dCA1 ML, +/ 1.6, vCA1 AP, 3.5, vCA1 ML, +/3.0).

Once the needle reached the appropriate depth (Bregma: dCA1 DV, - 1.3, vCA1 DV, 4.4), the injection robot was used to inject 2 μl of lentivirus (titers: eGFP-only, 10x106 TU/ml; eGFP-PDE11A4 7.4x106) suspended in 0.2M sucrose/42 mM NaCl/0.84 mM KCl/2.5 mM Na2HPO4/0.46 mM KH2PO4/0.35 mM EDTA at a rate of 0.167 μl/minute. The syringe was left in place for 3 minutes following injection before withdrawing the needle. Mice were allowed to recover for at least 2 weeks prior to behavioral testing.

RNA sequencing. As previously described [16], the Epigenomics Core at Weill Cornell Medicine performed the RNA and library preparation, sequencing, and post-processing of the raw data. The Functional Genomics Core of the University of South Carolina then performed the alignment and generation of the gene list of log-fold changes with their raw and FDR-corrected P-values. A cryoPREP TM Impactor was used to dry fracture tissue that had been stored at -80°C in a tissueTUBE device, using manufacturer recommendations (Covaris, Woburn, MA). Each pulverized sample was resuspended in 350uls of Qiagen RNeasy Plus Mini RTL lysis buffer and RNA extracted as per manufacturer recommendations (Qiagen, Valencia, CA). The Lab Chip GX (Perkin Elmer, Waltham, MA) assessed RNA integrity, with samples having a quality score >8.0. Established Illumina methods were used to prepare TruSeq RNA libraries (Illumina, San Diego, CA, Part #RS-122-2001). Samples were sequenced across 3 lanes, with each library being made with one of the TruSeq barcode index sequences. The pools were clustered at 6.5pM on a pair end read flow cell and sequenced for 100 cycles on an Illumina HiSeq 2500. Illumina's Real Time Analysis software (RTA) conducted primary processing of sequencing images. Image capture, base calling and demultiplexing was conducted using CASAVA 1.8.2 software. STAR v2.3.1 [17] was used to align sequences to the mouse mm10 genome (http://hgdownload.soe.ucsc.edu/goldenPath/mm10/bigZips/). Reads, which were summarized at exon, transcript, or gene level were, counted using the featureCounts function of the Subreads package [18] in R (https://www.R-project.org/) using Gencode M6 GTF (http://www.gencodegenes.org/mouse_stats/archive.html). Only those reads mapping uniquely to the genome

were used. A minimum threshold of 10 was used for mapping quality (MAPQ). The edgeR package within R [19] was used for differential expression analysis. The average read depth for the samples was 64 million reads, and only genes with at least 1 count per million average depth were considered for differential expression analysis. The trimmed mean of m-values (TMM) method was used to normalize raw counts. The estimateGLMRobustDisp function [20] was then used to calculate the dispersion estimates. The function glmFit [21] was then used to fit normalized read counts to a generalized linear model. The function glmLRT was then used to perform gene-wise tests for significant differential expression. Benjamini-Hochburg's FDR [22] was then used to correct the raw P-value for multiple testing. Genes that differed between WT versus KO mice with aLogFC >+/-0.5 and an FDR-P<0.01) were included in pathway analyses using String v11.0 (string-db.org) [23] (accessed 7/27/21).

2-dimensional difference in gel electrophoresis (2-D DIGE) and mass spectroscopy (MS). Phosphoproteomics studies were conducted by Applied Biomics (Hayward, CA; https://www.appliedbiomics.com/2d-dige/phosphoproteomics/). Ventral hippocampi from 4 cohorts of *Pde11a* WT and KO mice were harvested and homogenized as described above for Western blotting. Lysates for all WTs within a cohort (Cohort 1: 2F + 3M; Cohort 2: 3M; Cohort 3: 4F; Cohort 4: 6M), and all KOs within a cohort were pooled (Cohort 1: 2F + 3M; Cohort 2: 3M; Cohort 3: 4F; Cohort 4: 6M) to allow for sufficient starting material. Protein sample buffer was exchanged with 2D lysis buffer (30 mM Tris-HCl, pH 8.8, containing 7 M urea, 2 M thiourea and 4% CHAPS), with subsequent protein concentrations determined by Bio-Rad Protein Assay Kit II #500-0002 according to manufacturer's protocol and all sample concentrations adjusted to 6 mg/mL. These 4 sample sets were first characterized by a proteomic 2-D DIGE study, with total protein expression differences identified by MALDI-TOF MS and TOF/TOF tandem MS/MS reported elsewhere [13]. Subsequently, samples from Cohort 1 and 2 were combined as were samples from Cohort 3 and 4, to ultimately yield 2 WT samples and 2 KO samples with sufficient starting material for a phosphoproteomics study. For each sample, 30µg of protein was mixed

with the sample buffer (8 M urea, 4% CHAPS, 20 mg/ml DTT, 2% pharmalytes and trace amount of bromophenol blue) and 100 ul destreak solution and rehydration buffer (7 M urea, 2 M thiourea, 4% CHAPS, 20 mg/ml DTT, 1% pharmalytes and trace amount of bromophenol blue) for a total volume of 250 ul. Labeled samples were run with IEF (pH3-10 Linear) following the protocol provided by Amersham BioSciences, with IPG strips subsequently incubated for 15 minutes in freshly made equilibration buffer-1 (50 mM Tris-HCl, pH 8.8, containing 6 M urea, 30% glycerol, 2% SDS, trace amount of bromophenol blue and 10 mg/ml DTT) and rinsed for 10 minutes in freshly made equilibration buffer-2 (50 mM Tris-HCl, pH 8.8, containing 6 M urea, 30% glycerol, 2% SDS, trace amount of bromophenol blue and 45 mg/ml DTT). After rinsing in the SDS-gel running buffer, IPG strips were transferred into 12% SDS-gels that were run at 15°C until the dye front ran out of the gels. Gel images were scanned immediately following the SDS-PAGE using Typhoon TRIO (GE Healthcare), with the gels subsequently stained using Pro-Q ® Diamond Phosphoprotein Gel Stain following manufacturer's protocol (Invitrogen) and scanned again. Gels were analyzed using DeCyder v6.0 (GE Healthcare), along with manual inspection, to select differentially phosphorylated spots. Spots were then mapped in DeCyder and pixel counts exported for calculation of the phospho/total protein ratios. Spots with a KO/WT ratio $\leq$0.6 (with neither sample >0.7) or a ratio $\geq$1.58 (with neither sample <1.5) were subsequently identified by MALDI-TOF MS and TOF/TOF tandem MS/MS on an AB SCIEX TOF/TOF™ 5800 System (AB SCIEX). MALDI-TOF mass spectra were acquired in reflectron positive ion mode, averaging 4000 laser shots per spectrum. TOF/TOF tandem MS fragmentation spectra were acquired for each sample, averaging 4000 laser shots per fragmentation spectrum on each of the 10 most abundant ions present in each sample (excluding trypsin autolytic peptides and other known background ions). Peptide mass and the associated fragmentation spectra were then submitted to a GPS Explorer workstation equipped with the MASCOT search engine (Matrix science) to search the database of National Center for Biotechnology Information non-redundant. Signals with protein scores/C.I.% and total ion scores/C.I.% >100 were entered into String v11.0 (string-db.org; accessed 07/27/2021) for pathway analyses.

Methods for In vitro electron microscopy

2% paraformaldehyde/0.1% glutaraldehyde fixed cells were scraped from the surface of 100 mm dishes and embedded in 2% Agar. Thin slices of the agar pellets were placed in a Leica EM AFS2 outfitted with the EM FSP attachment and infiltrated with K11M resin (EMS 14350) using the progressive lowering of temperature (PLT) method outlined as per manufacturer's instructions. Modifications to the method included adding 1% p-phenylenediamine (Sigma P6001 (St. Louis, MO, USA)) to the 70% ethanol, a 36 hour acetone:0.1% uranyl acetate step added after the final ethanol dehydration, and resin infiltration performed with acetone in lieu of ethanol. The EM FSP was replaced with the LED UV lamp attachment and the K11M resin was cured at -60C for 8 hrs using a UV lamp on/off cycle of 30 min/10 min, followed by 18 hrs on, 16 hrs off as the temperature was raised to 20C and finally 48 hrs on. Cured blocks were cut on a Leica UltraCut R ultramicrotome and the gold colored sections (approx. 90 nm) collected on nickel grids. Grids were successively blocked with 0.05M glycine, 0.1% coldwater fish skin gelatin (EMS 25560), 5% BSA (JacksonImmuno 001-000-161) and 5% normal donkey serum (JacksonI 017-000-121). Sections were incubated with 1:5000 rabbit anti-GFP (abcam ab6556 (Cambridge, UK) in PBS/0.2% BSA-c (EMS 25557) overnight at 4C followed by 1:25 donkey anti-rabbit IgG:10 nm Au (EMS 25705) in PBS/0.2% BSA-c/0.1% Tween-20 (EMS 25564) for 2 hrs at room temperature. After a 10 min. fixation with 1% glutaraldehyde, sections were post-stained with 0.25% OsO_4 (aq) for 2 min., 1% uranyl acetate (aq) for 10 min. and Hanaichi calcined lead citrate for 4 min as per manufacturer's instructions. Sections were viewed with a JEM-1400 Plus (JEOL, USA) TEM and imaged with an AMT XR81 mid-mount camera.

Data Analysis. Data were collected blind to treatment and experiments were designed to counterbalance technical variables across biological variables. Data points greater than two standard deviations away from the mean were removed as outliers prior to analyses, as previously described (e.g., [5, 8, 24, 25]). Outliers removed/total n: Figure 1O, 1/24; Figure 3A, 4/89; Figure 3B, 3/73; Figure 3C, 7/110; Figure 3I, 4/73; Figure 3L, 2/43; Figure 3M, 5/4; Figure 4L, 2/48; Figure S6F, 2/38; Figure S7C, 5/108. Data were analyzed for effect of

genotype, age, behavioral parameter (e.g., bead, food, etc), and sex for experiments with greater than 6/sex/genotype [4, 5, 8, 12]). Where datasets met assumptions of normality (Shapiro-Wilk test) and equal variance (Levene's test), the following parametric statistical analyses were run on Sigmaplot 11.2 (San Jose, CA, USA): ANOVA (F), Student's t-test (t), one-sample t-test (t; Table 1). In the case of one-sample t-tests (determining whether or not a given group demonstrated memory), a false-rate discovery (FDR) correction was applied to all P-values within an experiment to mitigate the risk of Type I error associated with multiple comparisons. Where normality and/or equal variance assumptions failed, the following non-parametrical statistical analyses were run: Kruskal-Wallis ANOVA (H), Mann-Whitney rank sum test (T), or Wilcoxon Signed Rank Test (Z). Repeated measures analyses were used where appropriate (e.g., in analysis of behavior across multiple trials). *Post hoc* analyses were performed according to the Student-Newman-Keuls or Dunn's method and significance was defined as $P<0.05$.

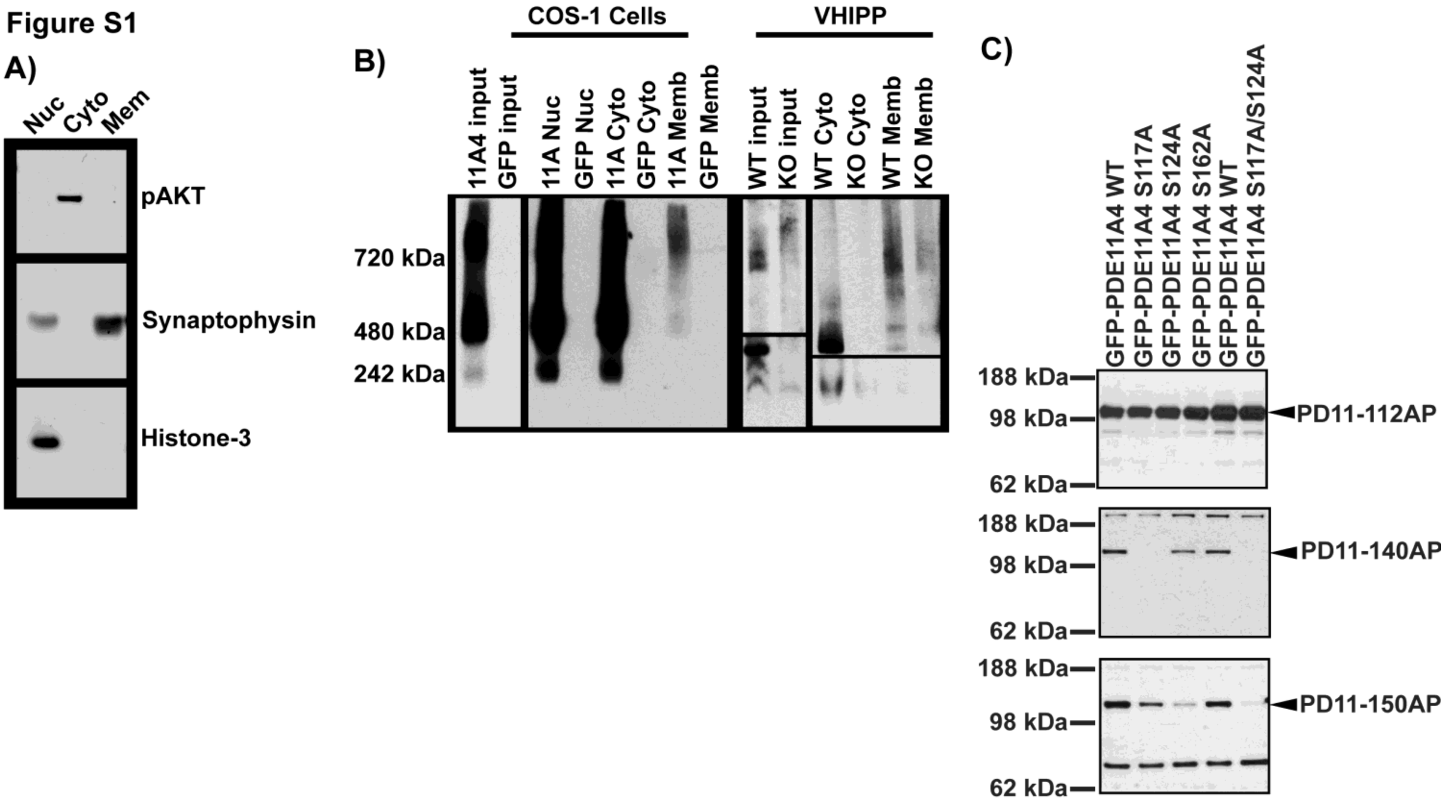

Figure S1. PDE11A4 dimers are only found in the nucleus and cytosol. A) Biochemical fractionation of VHIPP yields the expected distribution of fraction markers phospho-AKT (~60 kDa), synaptophysin (~39 kDa) and histone 3 (~17kDa). B) Lysates from COS-1 cells transfected with either GFP-mPDE11A4 (11A4) or GFP alone (GFP) as well as hippocampi from *Pde11a* WT and KO mice were run on a native gel (different portions of the film shown at different optimal exposures that yield no signal in the negative control samples). Bands corresponding to the molecular weight of a PDE11A4 homodimer (i.e, ~242 kDa for GFP-tagged PDE11A4 and ~200 kDa for endogenous PDE11A4) can be identified in inputs, nuclear fractions and cytosolic fractions but not membrane fractions. Additional specific bands are observed at ~450 kDa and above, pointing to the incorporation of PDE11A4 within macromolecular complexes as has been reported for other PDE families [26]. The fact that PDE11A4 dimers are not detected in the membrane fraction suggests PDE11A4 located in membrane fractions is not there via a direct interaction (i.e,. via palmitoylation, myristolation, etc), but rather via protein-protein interactions. C) Lysates from COS-1 cells were also loaded on a 4-12% Bis Tris denaturing gel to validate antibodies detecting total PDE11A4 (PD11-112AP), PDE11A4-pS117 (PD11-140AP) or PDE11A4-pS117/pS124 (PD11-150AP). Whereas PD11-112 detects wild-type (WT) and phosphoresistant versions of a GFP-PDE11A4 fusion protein around ~121 kDA (note: studies in *Pde11a* KO vs. WT mice show endogenous PDE11A4 migrates ~95 kDA), PD11-140AP recognizes WT, S124A, and S162A but not S117A nor S117A/S124A. PD11-150AP also recognizes WT and S162A, but shows a somewhat reduced signal with S117A, greatly reduced signal with S124A, and no signal with S117A/S124A. Together, this pattern suggests that PD11-150AP preferentially recognizes pS124 and weakly recognizes pS117. Brightness and/or contrast of images adjusted for graphical clarity.

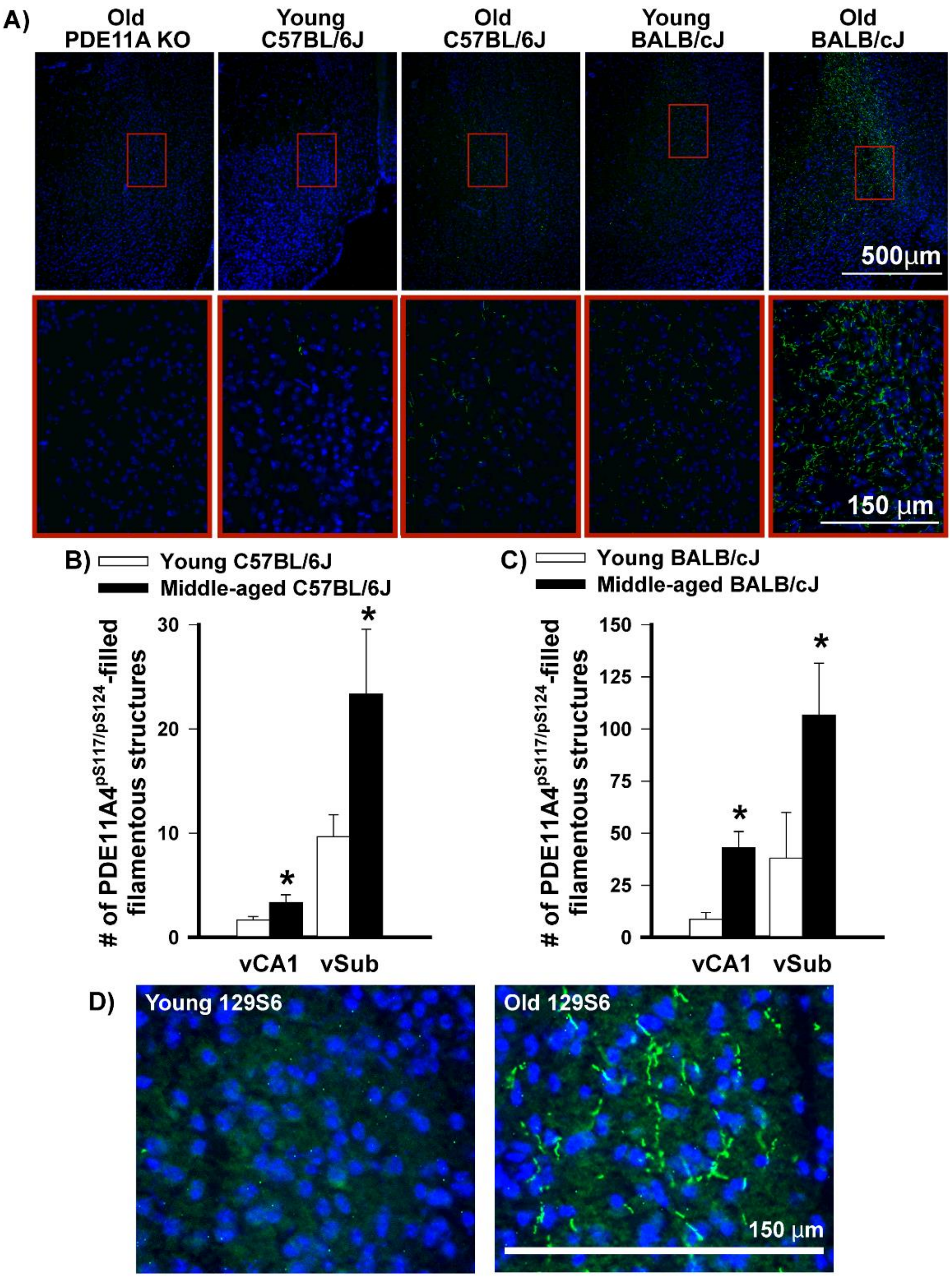

Figure S2. BALB/cJ and 129S6/SvEv mice show age-related increases in PDE11A-filled filamentous structures as observed in C57BL/6J mice. A) Immunofluorescence using an antibody that detects PDE11A4-pS117/pS124 (i.e., PD11-150AP) replicates and extends findings presented in Figure 1L-O by showing an age-related increase in PDE11A4-pS117/pS124 ghost axons occurs in both C57BL/6J and BALB/cJ mice. B) Quantification replicates the significant increase in PDE11A4-filled ghost axons that occurs in the C57BL/6J (n=3/age/sex) ventral CA1 (vCA1; failed normality; Rank Sum test for effect of age: $T(6,6)=26.5$, $P=0.041$) and ventral subiculum (vSub; failed normality; Rank Sum test for effect of age: $T(6,6)=25.0$, $P=0.026$) and C) shows this effect extends to vCA1 ($t(12)=-4.07$, $P=0.002$) and vSub (fails normality; Rank Sum test for effect of age: $T(7,7)=36.0$, $P=0.038$) of BALB/cJ mice (n=3M,4F/age). D) Qualitative assessments with antibodies recognizing total PDE11A4 (i.e, PD-112AP and 11A-#1) were also conducted in 129S6/SvEv mice (n=2M,1F/age), with PDE11A4-filled ghost axons rarely observed in vCA1 or vSub of young mice but plentiful in old mice. Together, these data suggest this phenomena is conserved across mouse strains. *vs. Young, P=0.041-0.002. Data plotted as mean ±SEM. Histogram stretch, brightness, and/or contrast of images adjusted for graphical clarity.

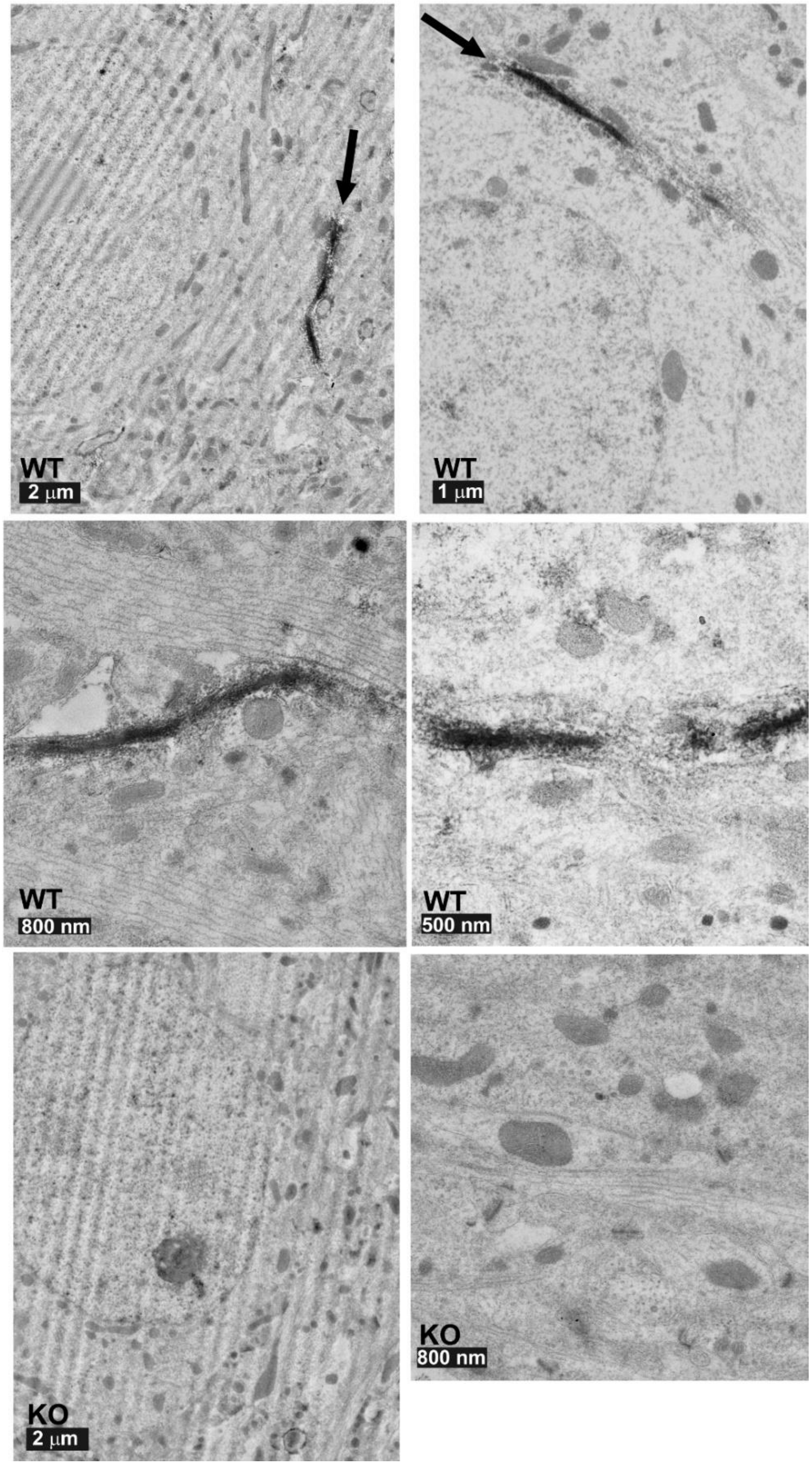

Figure S3. Electron microscopy (EM) suggest that age-related increases in PDE11A4 protein expression lead to an accumulation of PDE11A4 in axons that either occludes co-localization of other axonal proteins or possibly leads to the degeneration of the surrounding axon. Immunohistochemistry for PDE11A4 protein was conducted in ventral subiculum of *Pde11a* WT and KO mice followed by EM as per [27] by the Instrument Resource Facility at the University of South Carolina School of Medicine, except that 0.5% Triton X-100 was used (as in our normal immunoflourescence protocol) instead of the 0.04% triton that is normally used for electron microscopy. Unfortunately, this higher concentration of triton was necessary for the antibody to label the tissue, which led to compromising of the ultrastructure. Brightness and/or contrast of images adjusted for graphical clarity.

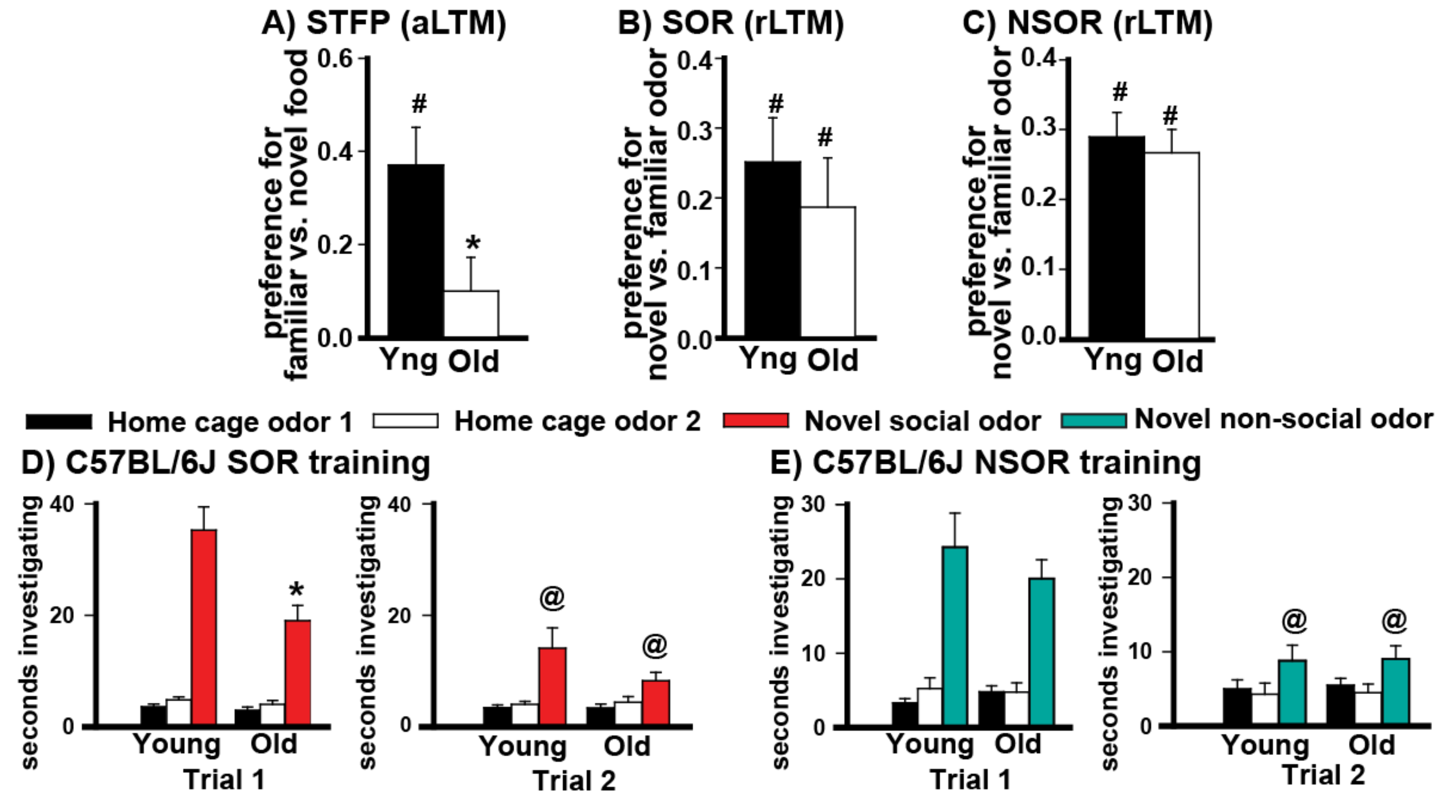

Figure S4. The fact that associative long-term memory (aLTM)—but not recognition long-term memory (rLTM)—is sensitive to age-related cognitive decline in humans [28-34] can be modeled in mice. A) 7 days after training, old mice (n=14M,9F) demonstrated worse remote aLTM for social transmission of food preference (STFP) than young mice (Yng; n=11/sex; effect of age: F(1,41)=4.98, P=0.031). Overall, old mice also ate less food than young mice (young 0.82 ±0.05 gm; old 0.69 ±0.1 gm; fails normality; Rank Sum test for effect of age: T(22,23)=616.5, P=0.012), which is consistent with reports of age-related deficits in homeostatic regulation of food intake [35-37]. B) In contrast, old mice (n=11M,12F) had equivalent remote rLTM relative to young mice (n=10M,12F) 7 days after training for social odor recognition (SOR; fails equal variance; Rank Sum test for effect of age: T(22, 23)=543.0, P=0.407), with old mice spending somewhat less time investigating the beads during the memory test (20.4 ±2.8 sec) relative to young mice (28.6 ±3.3 sec; fails normality; Rank Sum test for effect of age: T(22,23)=585, P=0.074). C) Old mice (n=6M,8F) also had equivalent remote non-social odor rLTM relative to young (n=7/sex) 7 days after training (effect of age: F(1,24)=0.33, P=0.574), with no difference in total time spent investigating the beads during the memory test (young 34.7 ±3.5 sec; old 29.0 ±3.3 sec; F(1,24)=1.62, P=0.215). D) Importantly, young and old mice demonstrated equivalent learning during SOR training, with a reduction in time spent exploring the novel bead from the first to the second training trial. That said, old mice spent less time exploring the novel SOR beads (effect of age x trial: F(1,41)=13.66, P=0.0006; *Post hoc*: trial 1 vs trial 2, P<0.001 for each age; old vs young trial 1, P=0.0009). E) Young and old mice also showed equivalent learning during NSOR training, with no differences in the total amount of time investigating the novel beads (effect of trial: F(1,24)=65.29, P<0.00001). *vs Young, P=0.031 to <0.001; @vs. Trial 1, P<0.001; m). [#]significant memory (i.e., greater than 0), FDR-P=0.015 to <0.001. Data plotted as mean ±SEM.

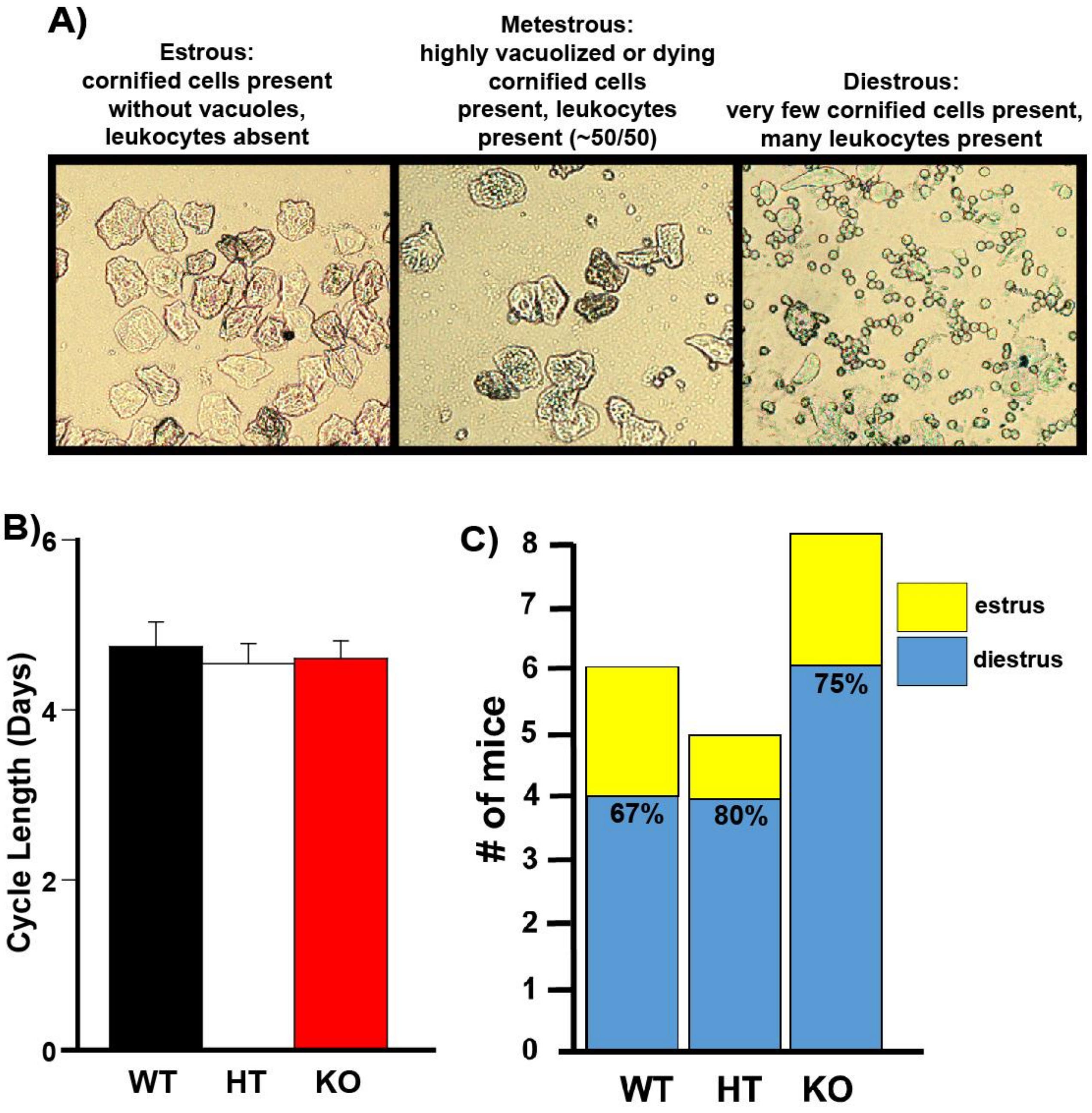

Figure S5. Deletion of PDE11A does not affect cycle length or reproductive senescence in female mice. As expected, vaginal smears conducted for a period of 12 days verified that young adult *Pde11a* WT, HT and KO females all demonstrate cycles of equivalent duration and all old *Pde11a* WT, HT and KO females similarly experience estropause (i.e., each genotype shows a similar distribution of mice paused in the persistent estrous vs. persistent diestrous phase). Indeed, cycling was unlikely to be a key driver of our PDE11A-related effects since 1) we see equivalent effect sizes and variability in our male and female mice, both in terms of age-related increases in PDE11A4 expression and in terms of the preservation of memory observed in our aged PDE11A mutant mice, and 2) the data shown herein were collected from multiple cohorts of mice tested across multiple years, which strongly decreases the likelihood that all females happen to have been tested at the same point in their cycle. Data in B plotted as mean ±SEM. Brightness and/or contrast of images adjusted for graphical clarity.

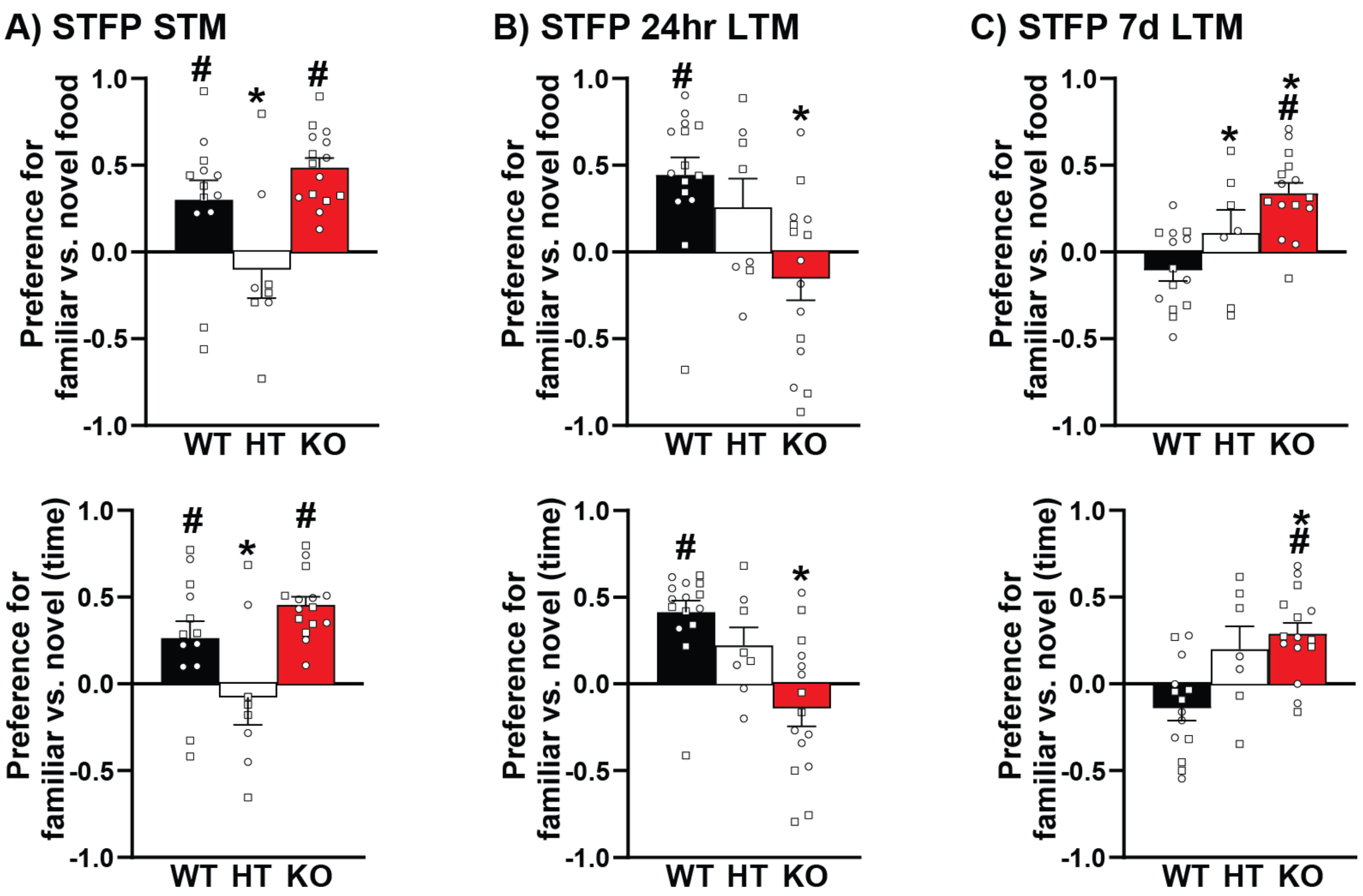

Figure S6. Deletion of PDE11A4 provides protection against age-related cognitive decline of social aLTMs in middle-aged (MA) mice 10-12 months old. A) WT-MA (n=7M,6F) and KO-MA mice (n=7M,8F) exhibited strong STM for STFP but HT-MA mice (n=5M,3F) did not (food effect of genotype: F(2,33)=7.32, P=0.002; *Post hoc* vs. HT-MA: WT-MA P=0.016, KO-MA P=0.002; time effect of genotype: F(2,33)=7.26, P=0.002; *Post hoc* vs. HT-MA: WT-MA P=0.023, KO-MA P=0.002). That said, the memory phenotype in the HT-MA mice is confounded by the fact that they ate significantly less food (0.54 ±0.06 gm) relative to WT-MA (0.74 ±0.08 gm) and KO-MA (0.82 ±0.06 gm; effect of genotype: F(2,33)=3.69, P=0.036). B) WT-MA mice continued to show a strong recent aLTM 24 hours after training; however, KO mice demonstrated transient amnesia (food effect of genotype: F(2,35)=7.00, P=0.003; *Post hoc* vs KO-MA: WT-MA P=0.002, HT-MA P=0.041; time effect of genotype: F(2,35)=11.04, P=0.0002; *Post hoc* vs. KO-MA: WT-MA P=0.0002, HT-MA P=0.016). Despite having no memory, KO-MA ate the same total amount of food (0.75 ±0.07 gm) as did WT-MA (0.78 ±0.09 gm) and HT-MA mice (0.71 ±0.13 gm; effect of genotype: F(2,35)=0.12, P=0.885). C) In contrast, HT-MA (n= 5M,2F) and KO-MA mice (n=7M,8F) demonstrated stronger remote aLTM 7 days after training relative to WT-MA mice (n=7M,7F; food effect of genotype: F(2,33)=9.17, P=0.0007; *Post hoc* vs. WT-MA: HT-MA P=0.012, KO-MA P=0.0007; time effect of genotype: F(2,33)=10.64, P=0.0003; *Post hoc* vs. WT-MA: HT-MA P=0.08, KO-MA P=0.0002). There was no difference between WT-MA (0.58 ±0.07 gm), HT-MA (0.49 ±0.07 gm) and KO-MA (0.70 ±0.08 gm) in terms of the total amount of food eaten during the remote LTM test (fails normality; ANOVA on Ranks for effect of genotype: H(2)=1.77, P=0.412). #significant memory (i.e., greater than 0), FDR-P=0.029 to <0.001; *vs WT -MA, P=0.023 to <0.001. Data plotted as individual points (females as circles, males as squares) and expressed as mean ±SEM.

Figure S7

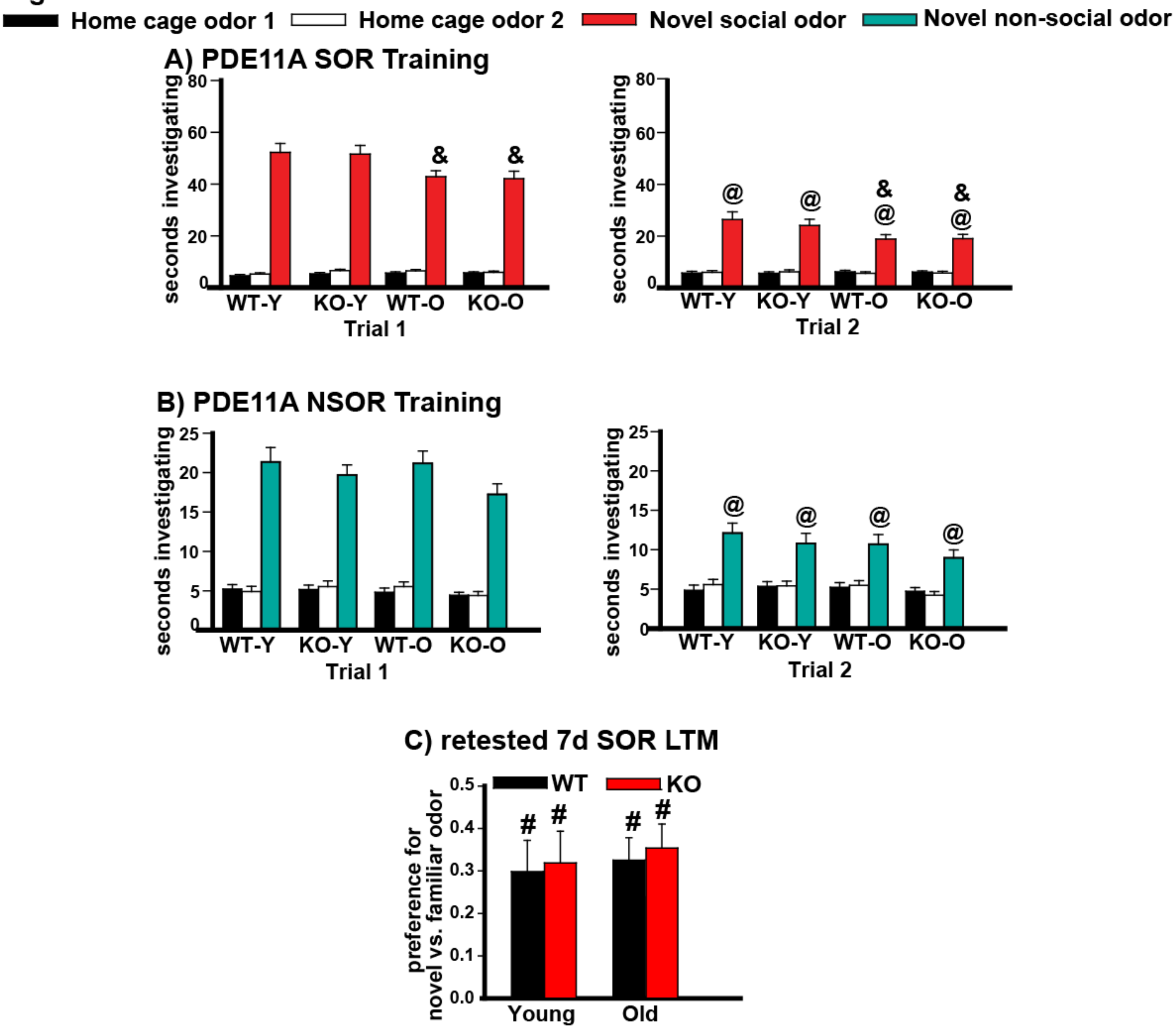

Figure S7. ***Pde11a*** **KO and WT mice exhibit equivalent learning during training for social odor recognition (SOR) and non-social odor recognition (NSOR)**. A) During SOR training (combined data for STM, 24hr and 7d test groups shown in Figure 3), there was an effect of age—but not genotype nor sex—on the total amount of time mice spent investigating the novel-scented bead (3-factor with RM ANOVA across trials fails normality; 3 Way ANOVA on trial 1 effect of age: $F(1,169)=10.01$, $P=0.0018$) and trial 2 (3 Way ANOVA on trial 2 fails normality and equal variance; Rank Sum effect of age: $T(87,90)=8490$, $P=0.028$). That said, all groups learned as indicated by a significant reduction in the amount of time spent exploring the novel scent during trial 2 vs. trial 1 (Wilcoxon Signed Rank Test for effect of trial: $Z=-11.45$, $P<0.0001$). B) During NSOR training (combined data for STM, 24hr and 7d test groups), there were no significant differences in the total amount of time each group spent exploring the novel bead during trial 1 or trial 2, and all groups learned as indicated by a significant reduction in the amount of time spent exploring the novel scent during trial 2 vs. trial 1 (3-factor with RM ANOVA fails normality; Wilcoxon Signed Rank test for effect of trial: $Z=-9.94$, $P<0.0001$). C) When mice are tested for STM or recent LTM for SOR (from Figure3D-E) and then retested for remote LTM 7 days after SOR training, WT-Young (n=12M,11F), WT-Old (n=14M,15F), KO-Young (n=13M,10F) and KO-Old mice (14M,14F) all show strong memory that does not differ from one another, although overall males

demonstrated stronger memory than females (effect of sex: $F(1,95)=4.51, P=0.036$). This stands in contrast to the enhanced memory that can be observed in KO versus WT mice when mice are tested for the first time on day 7 (Figure 3F). The ability to detect an enhancement in KOs when tested for the first time on day 7 is due to the decline in memory that is seen in the WT mice in absence of an earlier retrieval test (Figure 1F), and is consistent with our previous observation in young mice alone [5]. $^{@}$main effect of trial (i.e., trial 2 vs trial 1 across groups), $P<0.001$; $^{\&}$main effect of age (i.e., old versus young across genotypes), $P=0.028$-0.001; $^{\#}$significant memory (i.e., greater than 0), FDR-$P<0.001$. Data plotted as mean ±SEM.

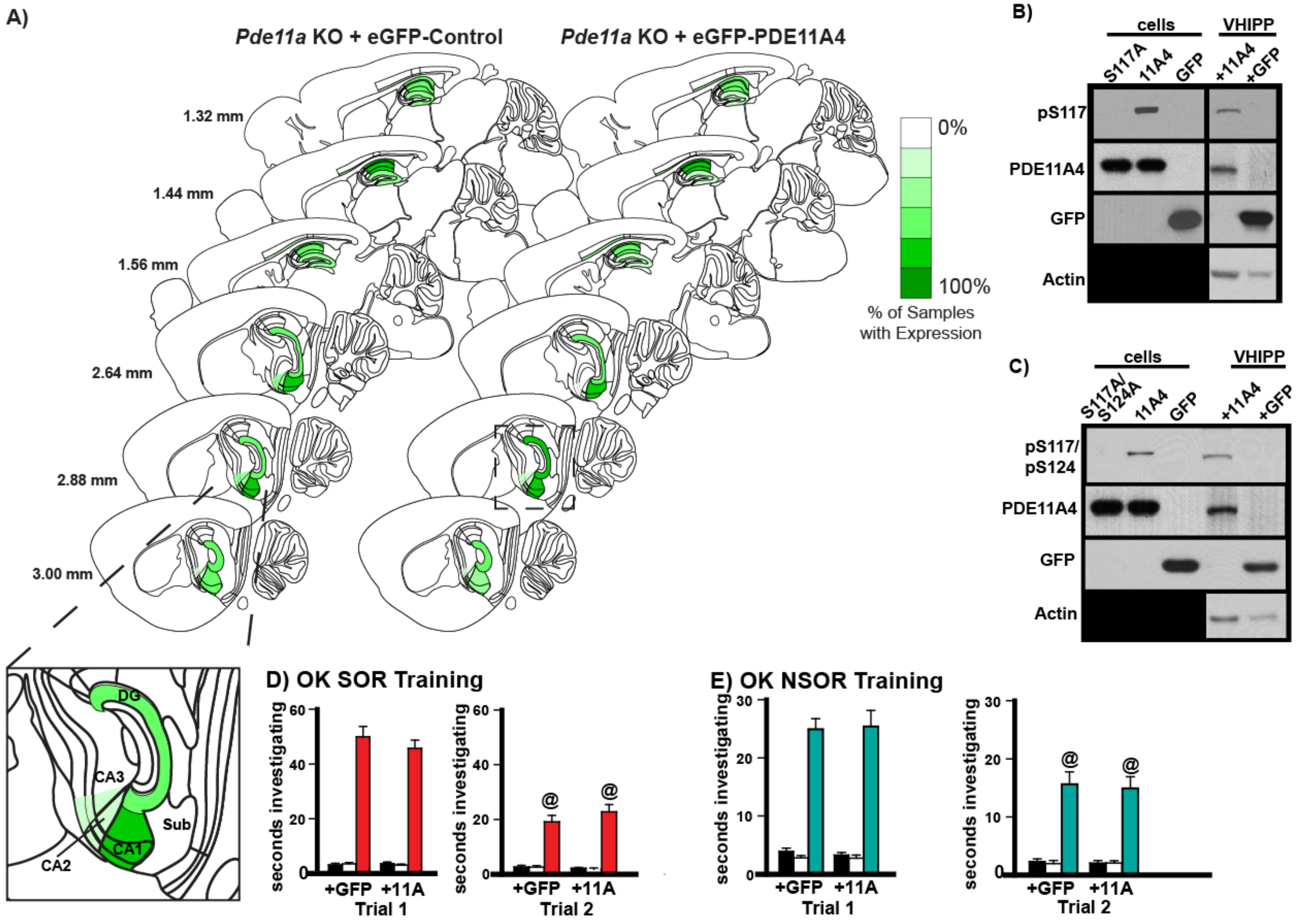

Figure S8. Recombinant GFP-mPDE11A4 performs similarly to endogenous PDE11A4. A) Mapping of GFP expression observed >2 months following injection shows all mice received a targeted injection of dorsal and ventral CA1, with a minority of mice showing spread outside the targeted subfield. B) Recombinant PDE11A4 is phosphorylated at S117 and S124 in COS-1 cells and the ventral hippocampus (i.e., *in vivo*). Antibody signals are specific when compared to expression of GFP-alone (in cells and hippocampus) or expression of the S117A or S117AS124A phosphoresistant mutants (COS-1 cells). Molecular weights: GFP-tagged PDE11A4 constructs = ~124 kDa; GFP alone= 27 kDa; Actin = ~45 kDa. C) KO-11A4 mice (n = 10M,12F) and KO-GFP mice (n= 8M,11F) learned equally well during training for SOR (effect of group: F(2,41)=0.008, P=0.93; effect of trial: F(1,41)=111.75, P<0.0001), and E) NSOR (KO-11A4 n=10M,12F; KO-GFP, n=8M,11F; effect of group: F(1,37)=0.02, P=0.897; effect of trial: F(1,37)=77.63, P<0.0001). @main effect of trial (i.e, vs. Trial 1), P<0.001. Brightness & contrast adjusted for clarity. Data plotted as mean ±SEM.

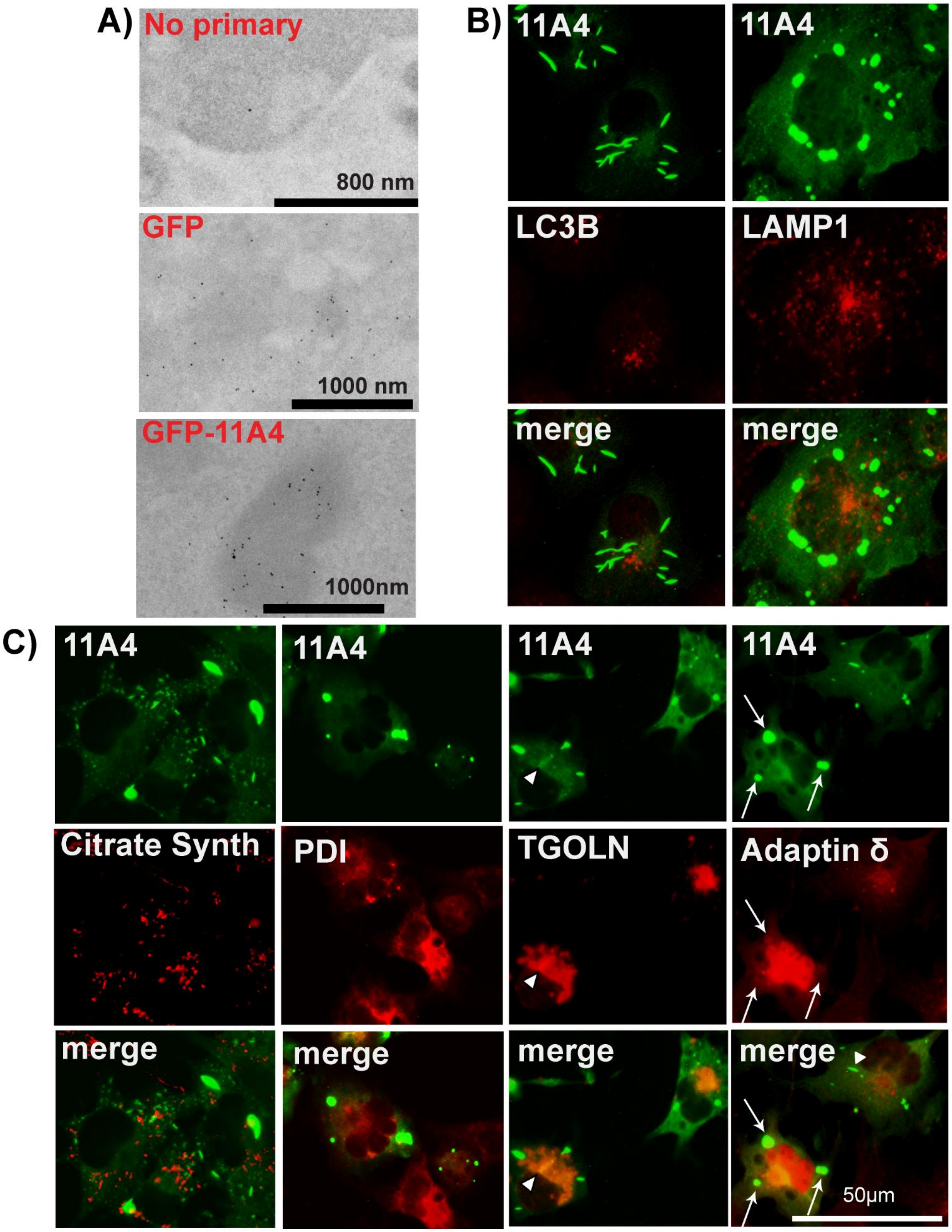

Figure S9. PDE11A4 accumulates in vitro in electron dense structures that fail to colocalize with typical organelle markers. A) Electron microscopy reveals GFP-mPDE11A4 (GFP-11A4) accumulates within electron-dense structures in COS-1 cells fixed twenty hours after transfection (i.e., 5 hours after replacement of transfection media with fetal bovine serum-supplement DMEM). In contrast, COS-1 cells transfected with GFP

alone show labeling throughout the cell. Antibody specificity verified by a no-primary condition. B) Immunocytochemistry of autophagosomes with LC3B and of lysosomes with LAMP1 exhibits no colocalization with GFP-mPDE11A4. C) Co-labeling of GFP-mPDE11A4 with a marker of mitochondria (citrate synthase) or endoplasmic reticulum (PDI), show no colocalization. Co-transfection of cells with GFP-mPDE11A4 and TGOLN-C-tRFP indicates that puncta are not within the Golgi apparatus; however, a pool of non-punctate GFP-mPDE11A4 overlaps with TGOLN-C-tRFP. Finally, some puncta appear to colocalize with adaptin δ, a marker of intracellular transport vesicles. Note that while COS-1 cells are not themselves secretory cells, recombinant expression of regulated cargo proteins is sufficient to generate secretory granules therein [38]. Antibody specificity verified by no-primary conditions (not shown). Colocalization indicated by arrows; non-colocalized puncta indicated by triangle. Histogram stretch, brightness, and/or contrast of images adjusted for graphical clarity.

SUPPLEMENTAL REFERENCES

1. Farmer, R., et al., *Phosphodiesterases PDE2A and PDE10A both change mRNA expression in the human brain with age, but only PDE2A changes in a region-specific manner with psychiatric disease.* Cell Signal, 2020. **70**: p. 109592.
2. Patel, N.S., et al., *Identification of new PDE9A isoforms and how their expression and subcellular compartmentalization in the brain change across the life span.* Neurobiol Aging, 2018. **65**: p. 217-234.
3. Miller, J.A., et al., *Transcriptional landscape of the prenatal human brain.* Nature, 2014. **508**(7495): p. 199-206.
4. Kelly, M.P., et al., *Phosphodiesterase 11A in brain is enriched in ventral hippocampus and deletion causes psychiatric disease-related phenotypes.* Proc Natl Acad Sci U S A, 2010. **107**(18): p. 8457-62.
5. Pilarzyk, K., et al., *Loss of Function of Phosphodiesterase 11A4 Shows that Recent and Remote Long-Term Memories Can Be Uncoupled.* Curr Biol, 2019. **29**(14): p. 2307-2321 e5.
6. Smith, A.J., et al., *A genetic basis for friendship? Homophily for membrane-associated PDE11A-cAMP-CREB signaling in CA1 of hippocampus dictates mutual social preference in male and female mice.* Mol Psychiatry, 2021.
7. Porcher, L., et al., *Aging triggers an upregulation of a multitude of cytokines in the male and especially the female rodent hippocampus but more discrete changes in other brain regions.* J Neuroinflammation, 2021. **18**(1): p. 219.
8. Kelly, M.P., et al., *Select 3',5'-cyclic nucleotide phosphodiesterases exhibit altered expression in the aged rodent brain.* Cell Signal, 2014. **26**(2): p. 383-97.
9. Kelly, M.P., *Putting together the pieces of phosphodiesterase distribution patterns in the brain: A jigsaw puzzle of cyclic nucleotide regulation.*, in *Cyclic Nucleotide Phosphodiesterases in the Central Nervous System: From Biology to Disease*, N.J. Brandon and A.R. West, Editors. 2014, John Wiley & Sons, Inc: New Jersey.
10. Pathak, G., et al., *PDE11A negatively regulates lithium responsivity.* Mol Psychiatry, 2017. **22**(12): p. 1714-1724.
11. Fosang, A.J. and R.J. Colbran, *Transparency Is the Key to Quality.* J Biol Chem, 2015. **290**(50): p. 29692-4.
12. Hegde, S., et al., *Phosphodiesterase 11A (PDE11A), Enriched in Ventral Hippocampus Neurons, is Required for Consolidation of Social but not Nonsocial Memories in Mice.* Neuropsychopharmacology, 2016. **41**(12): p. 2920-2931.
13. Pilarzyk, K., et al., *The Role of PDE11A4 in Social Isolation-Induced Changes in Intracellular Signaling and Neuroinflammation.* Front Pharmacol, 2021. **12**: p. 749628.
14. Jackson, R.J., M.T. Howell, and A. Kaminski, *The novel mechanism of initiation of picornavirus RNA translation.* Trends Biochem Sci, 1990. **15**(12): p. 477-83.
15. Smith, A.J., et al., *A genetic basis for friendship? Homophily for membrane-associated PDE11A-cAMP-CREB signaling in CA1 of hippocampus dictates mutual social preference in male and female mice.* Molecular Psychiatry, 2021.
16. Hegde, S., et al., *PDE11A regulates social behaviors and is a key mechanism by which social experience sculpts the brain.* Neuroscience, 2016. **335**: p. 151-69.
17. Dobin, A., et al., *STAR: ultrafast universal RNA-seq aligner.* Bioinformatics, 2013. **29**(1): p. 15-21.
18. Liao, Y., G.K. Smyth, and W. Shi, *The Subread aligner: fast, accurate and scalable read mapping by seed-and-vote.* Nucleic Acids Res, 2013. **41**(10): p. e108.

19. Robinson, M.D., D.J. McCarthy, and G.K. Smyth, *edgeR: a Bioconductor package for differential expression analysis of digital gene expression data.* Bioinformatics, 2010. **26**(1): p. 139-40.
20. Zhou, X., H. Lindsay, and M.D. Robinson, *Robustly detecting differential expression in RNA sequencing data using observation weights.* Nucleic Acids Res, 2014. **42**(11): p. e91.
21. McCarthy, D.J., Y. Chen, and G.K. Smyth, *Differential expression analysis of multifactor RNA-Seq experiments with respect to biological variation.* Nucleic Acids Res, 2012. **40**(10): p. 4288-97.
22. Benjamini, Y.H., Y., *Controlling the False Discovery Rate: A Practical and Powerful Approach to Multiple Testing.* Journal of the Royal Statistical Society, Series B (Methodological), 1995. **57**(1): p. 289-300.
23. Szklarczyk, D., et al., *STRING v10: protein-protein interaction networks, integrated over the tree of life.* Nucleic Acids Res, 2015. **43**(Database issue): p. D447-52.
24. Pathak, G., et al., *Amphetamine sensitization in mice is sufficient to produce both manic- and depressive-related behaviors as well as changes in the functional connectivity of corticolimbic structures.* Neuropharmacology, 2015. **95**: p. 434-47.
25. Kelly, M.P., et al., *The supra-additive hyperactivity caused by an amphetamine-chlordiazepoxide mixture exhibits an inverted-U dose response: negative implications for the use of a model in screening for mood stabilizers.* Pharmacol Biochem Behav, 2009. **92**(4): p. 649-54.
26. Baillie, G.S., G.S. Tejeda, and M.P. Kelly, *Therapeutic targeting of 3',5'-cyclic nucleotide phosphodiesterases: inhibition and beyond.* Nat Rev Drug Discov, 2019. **18**(10): p. 770-796.
27. McDonald, A.J., G.C. Jones, and D.D. Mott, *Diverse glutamatergic inputs target spines expressing M1 muscarinic receptors in the basolateral amygdala: An ultrastructural analysis.* Brain Res, 2019. **1722**: p. 146349.
28. Bender, A.R., M. Naveh-Benjamin, and N. Raz, *Associative deficit in recognition memory in a lifespan sample of healthy adults.* Psychol Aging, 2010. **25**(4): p. 940-8.
29. Old, S.R. and M. Naveh-Benjamin, *Differential effects of age on item and associative measures of memory: a meta-analysis.* Psychol Aging, 2008. **23**(1): p. 104-18.
30. Bridger, E.K., et al., *Age effects on associative memory for novel picture pairings.* Brain Res, 2017. **1664**: p. 102-115.
31. Troyer, A.K., et al., *Age-related differences in associative memory depend on the types of associations that are formed.* Neuropsychol Dev Cogn B Aging Neuropsychol Cogn, 2011. **18**(3): p. 340-52.
32. Overman, A.A. and J.T. Becker, *The associative deficit in older adult memory: Recognition of pairs is not improved by repetition.* Psychol Aging, 2009. **24**(2): p. 501-6.
33. Hartman, M. and L.H. Warren, *Explaining age differences in temporal working memory.* Psychol Aging, 2005. **20**(4): p. 645-56.
34. Hargis, M.B. and A.D. Castel, *Younger and older adults' associative memory for social information: The role of information importance.* Psychol Aging, 2017. **32**(4): p. 325-330.
35. Wolden-Hanson, T., *Mechanisms of the anorexia of aging in the Brown Norway rat.* Physiol Behav, 2006. **88**(3): p. 267-76.
36. Black, B.J., Jr., et al., *Senescent terminal weight loss in the male F344 rat.* Am J Physiol Regul Integr Comp Physiol, 2003. **284**(2): p. R336-42.
37. Kessler, B.A., et al., *Age-related loss of orexin/hypocretin neurons.* Neuroscience, 2011. **178**: p. 82-8.
38. Beuret, N., et al., *Expression of regulated secretory proteins is sufficient to generate granule-like structures in constitutively secreting cells.* J Biol Chem, 2004. **279**(19): p. 20242-9.
