# Supplementary material for: Conserved age‐related increases in hippocampal PDE11A4 cause unexpected proteinopathies and cognitive decline of social associative memories
Source: Aging Cell. 2022 Sep 8;21(10):e13687. doi: 10.1111/acel.13687 (PMC9577960; doi:10.1111/acel.13687)

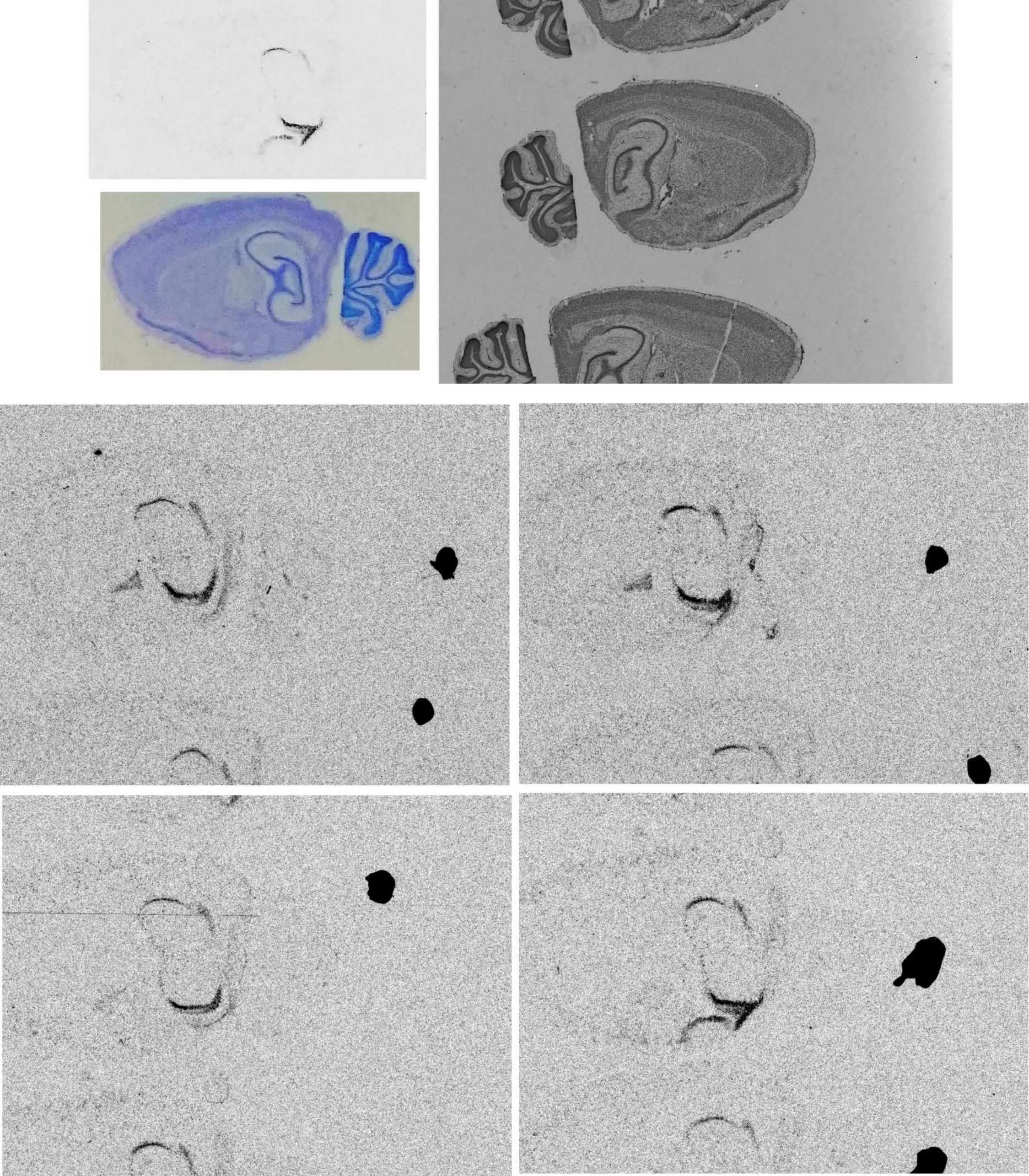

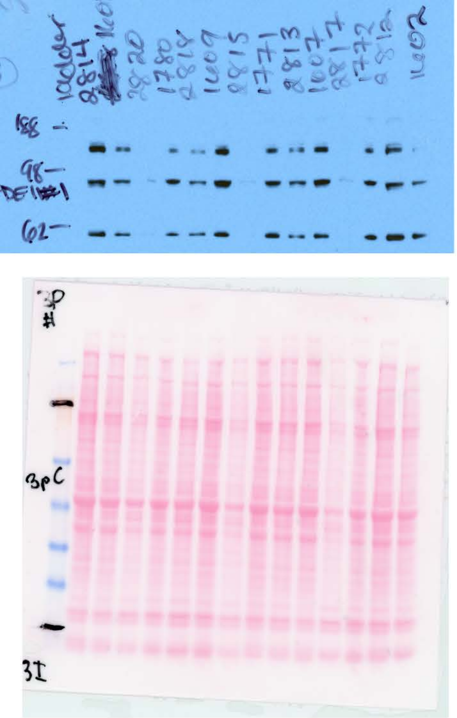
188
98
PDE11#1
62
3pC
3I

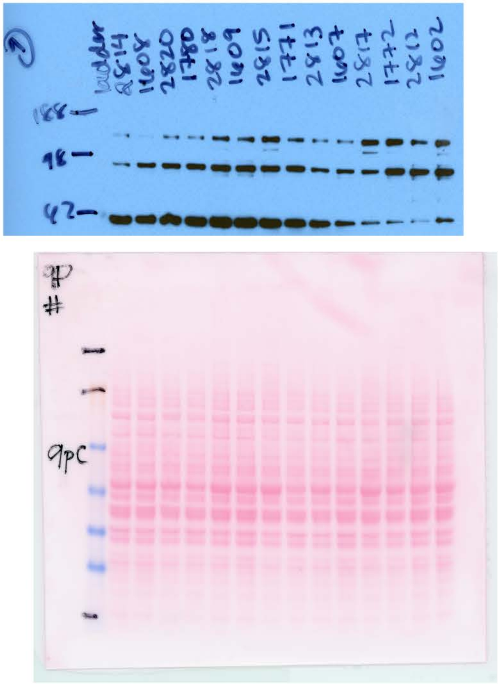
ladder
2814
1608
2820
1780
2818
1609
2815
1771
2813
1607
2817
1772
2812
1602
188
98
62
9pC

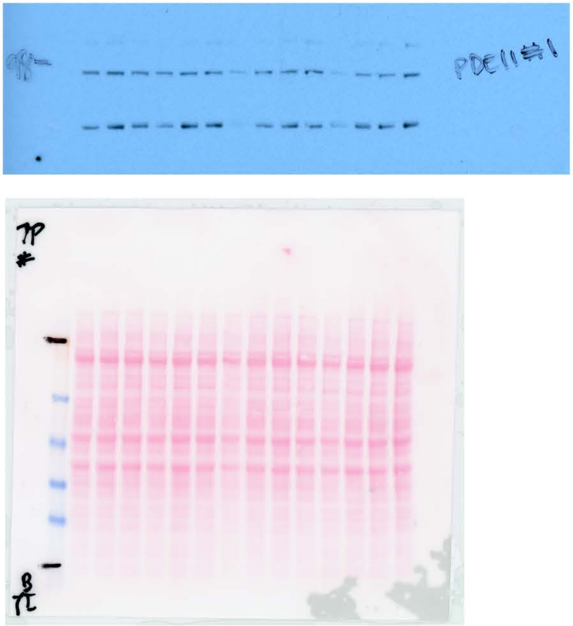
98
PDE11#1

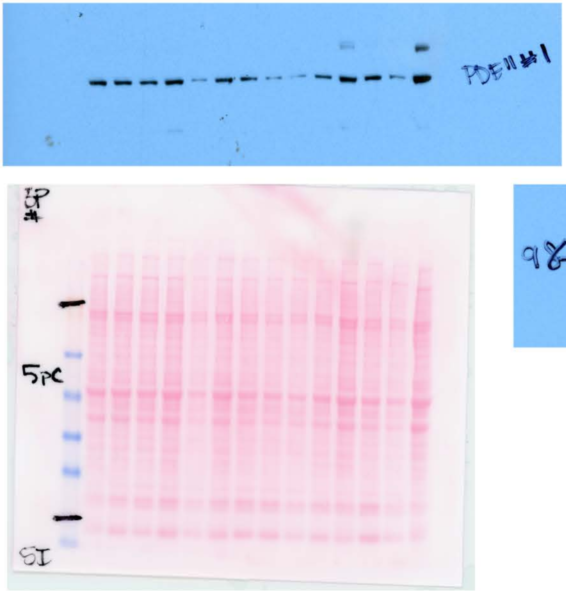
PDE11#1
5pC
5I

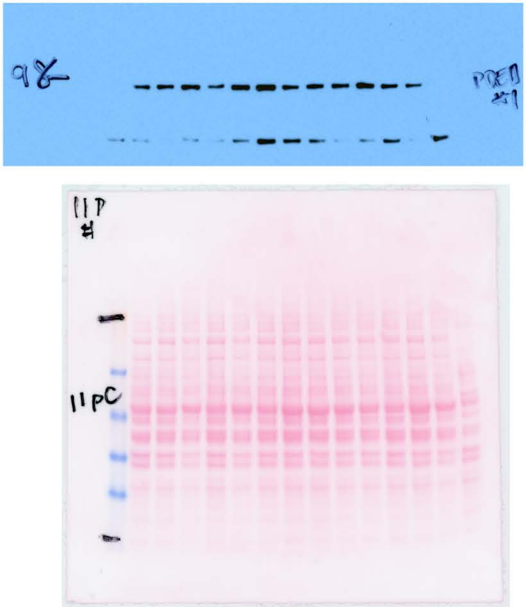
98
PDE11 #1
11pC

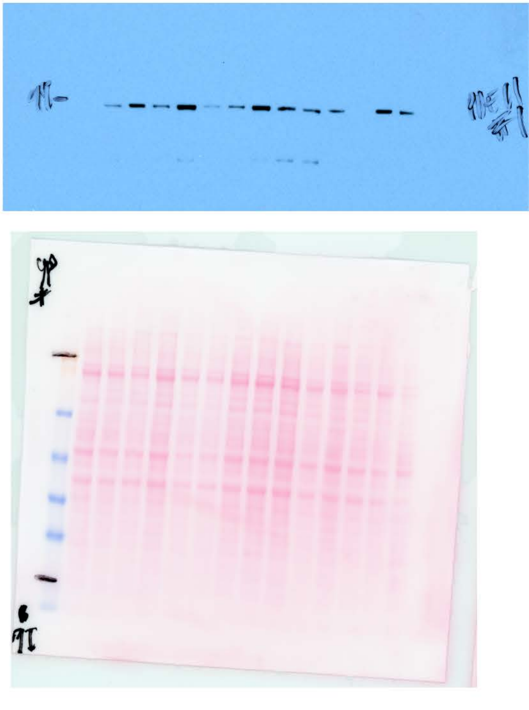
98
PDE11 #1
9I

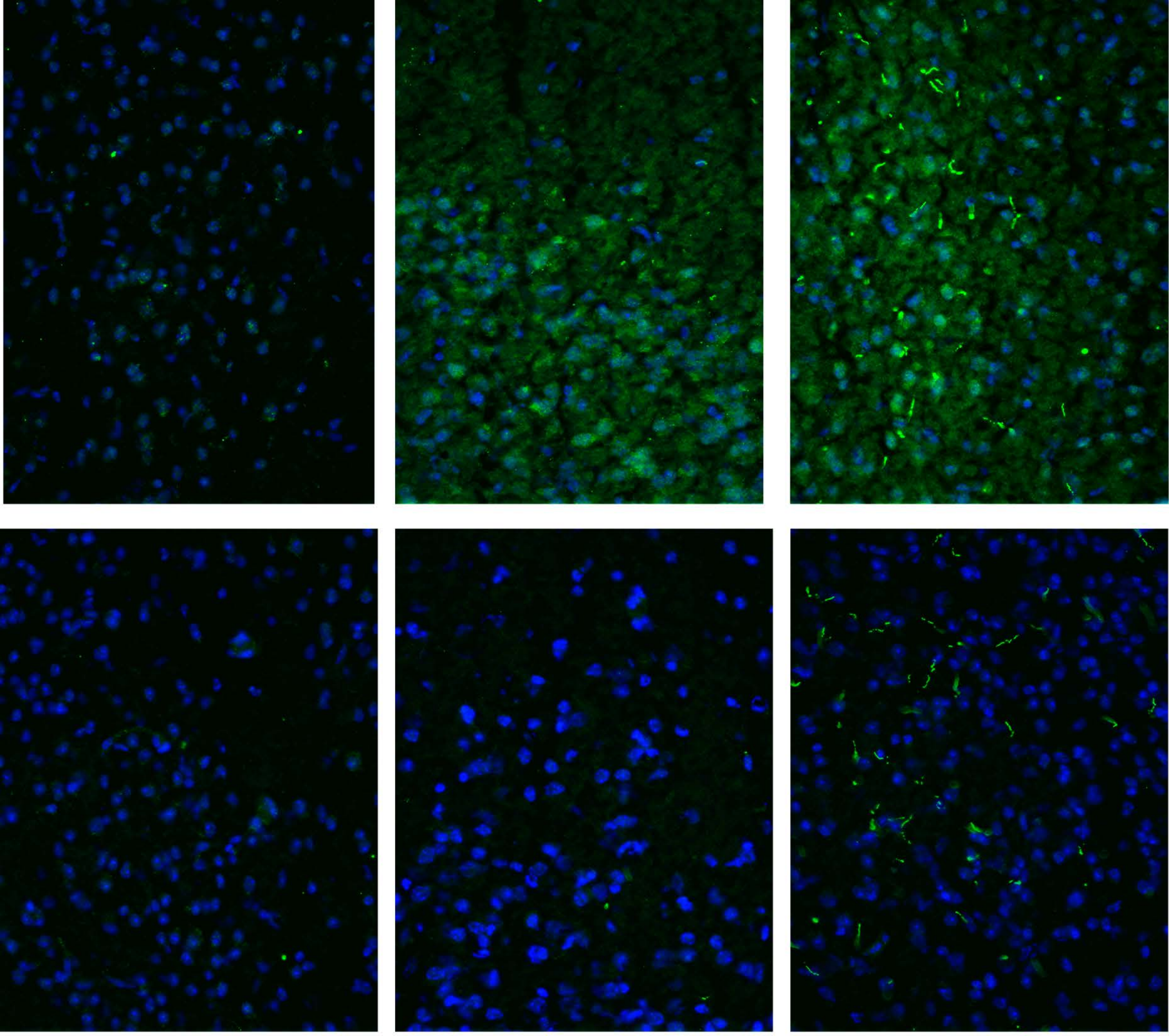

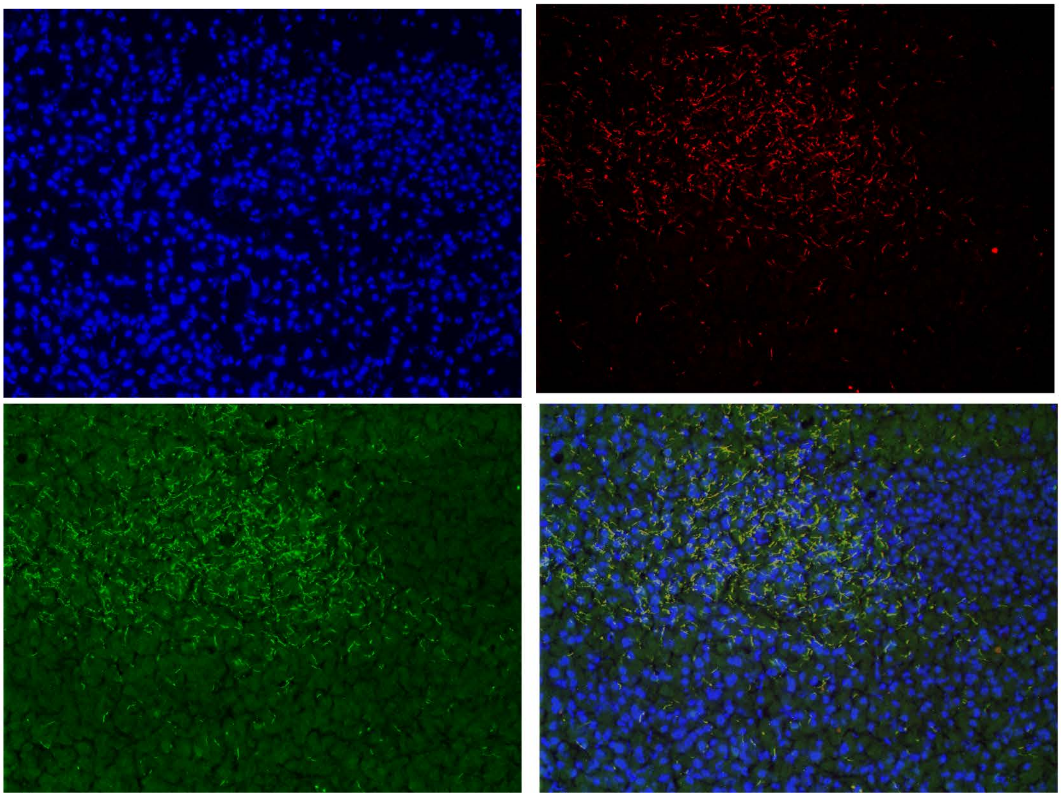

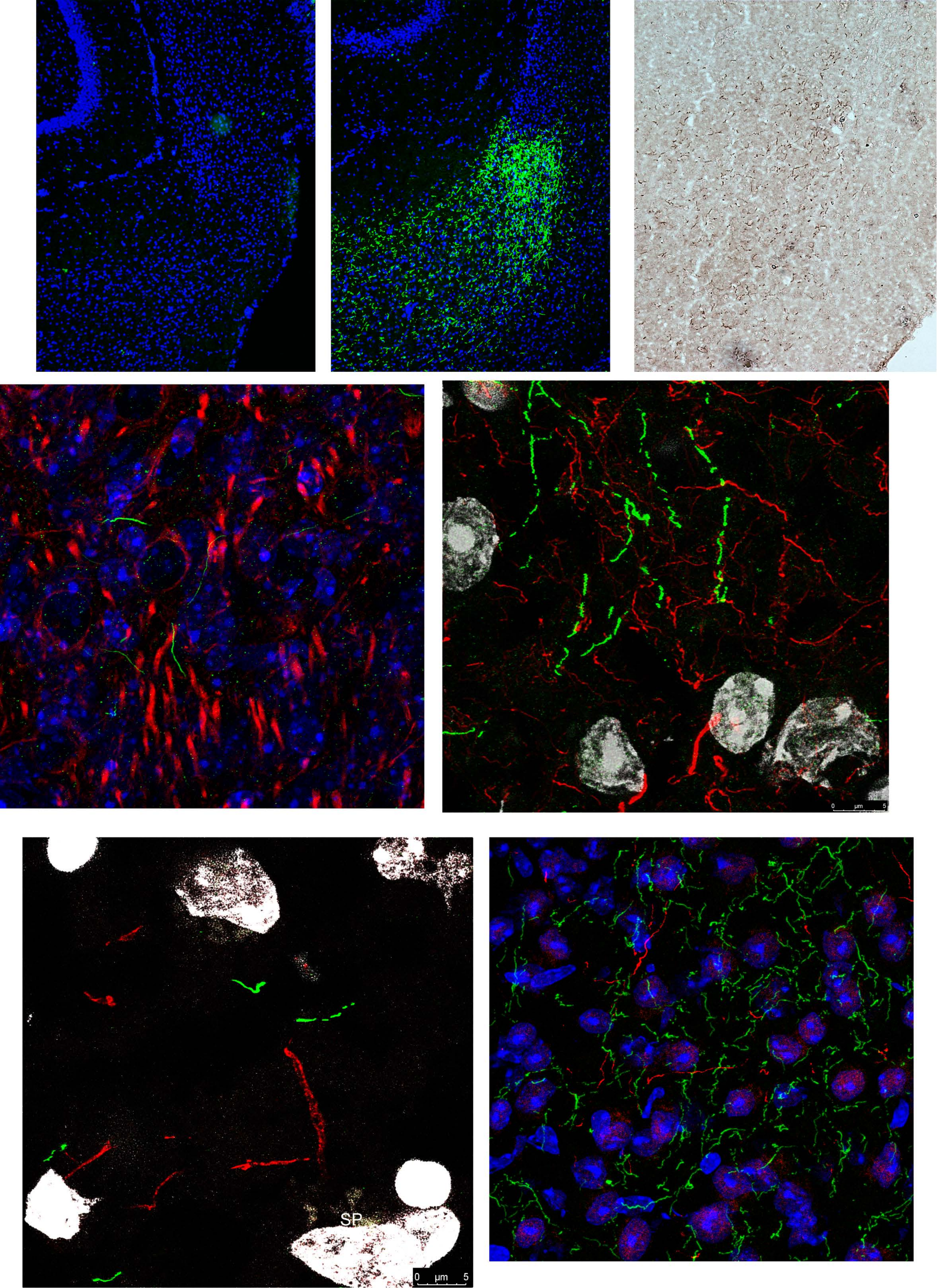
0 µm 5
SP
0 µm 5

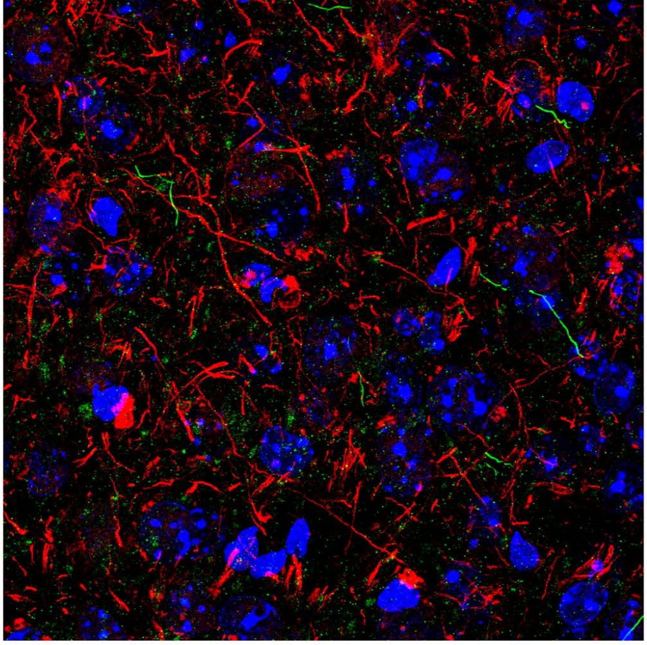
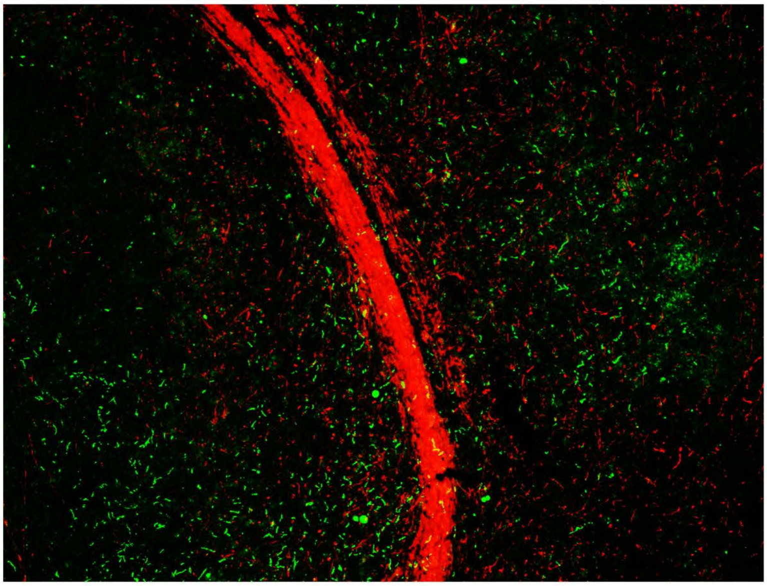
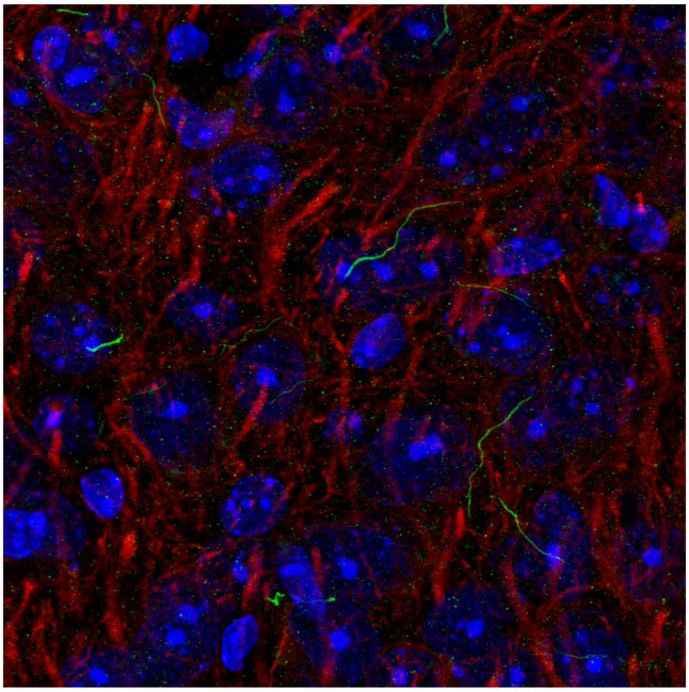
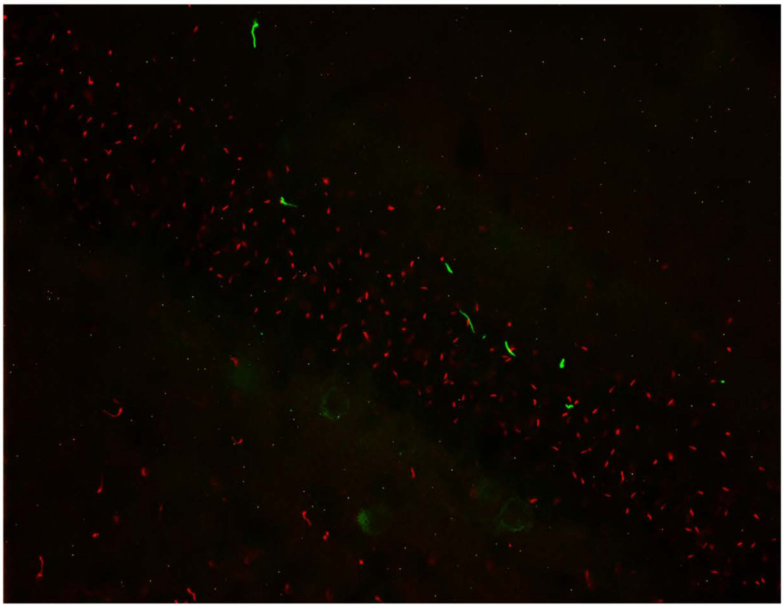
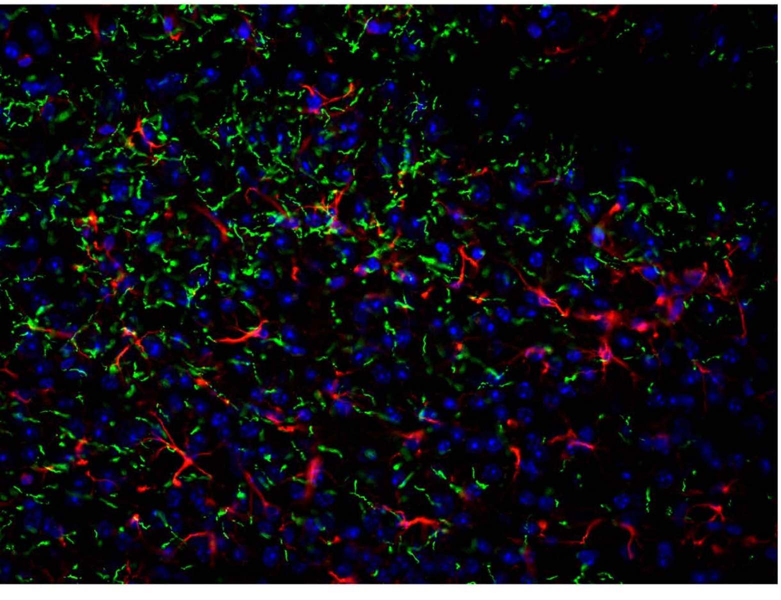
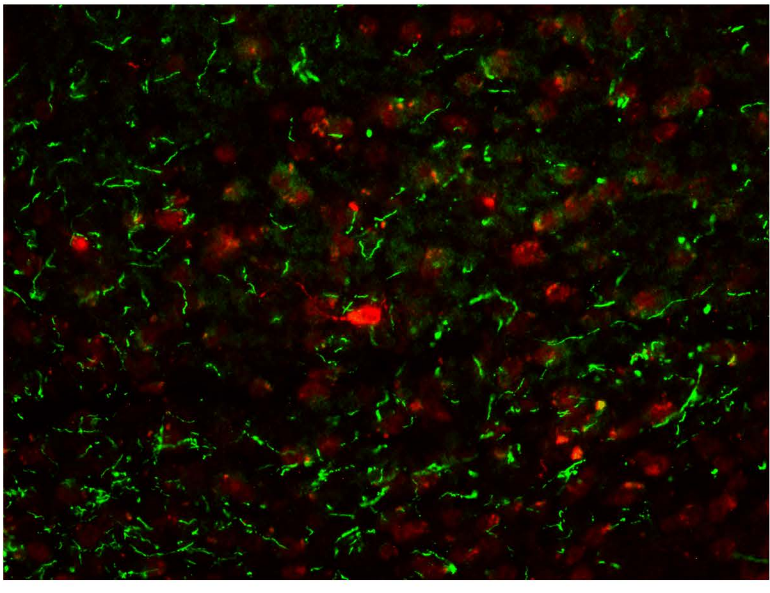

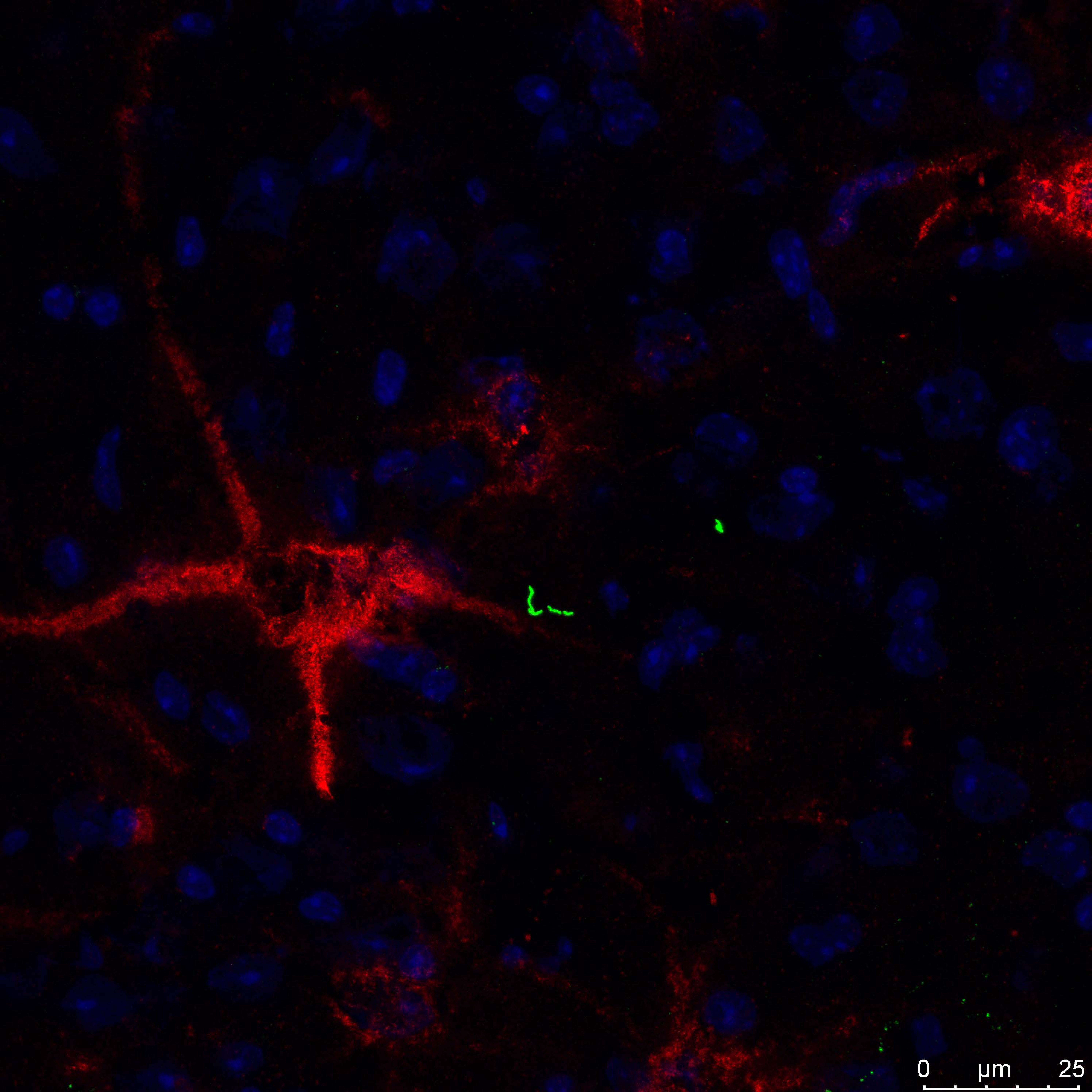
0 µm 25

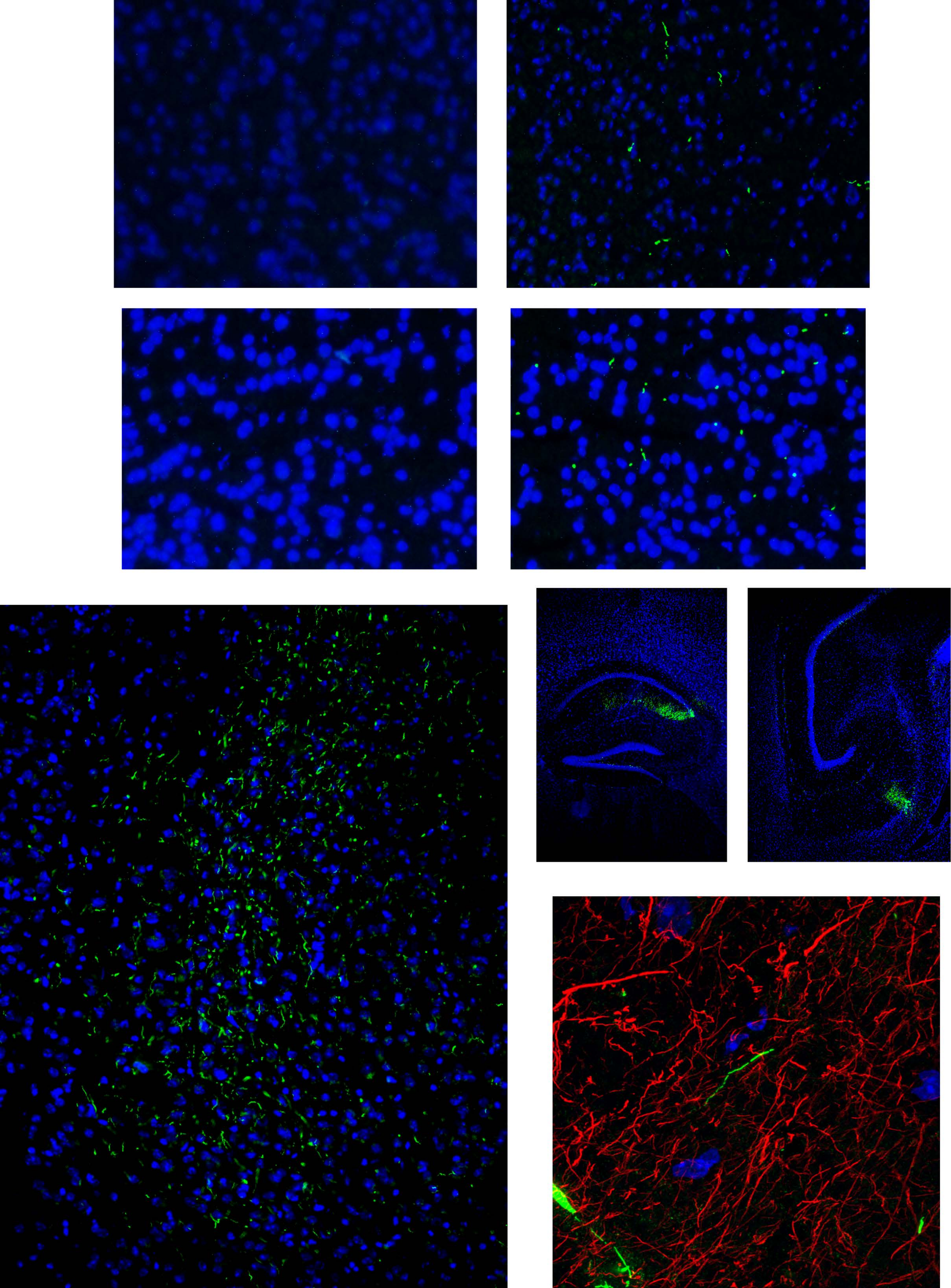

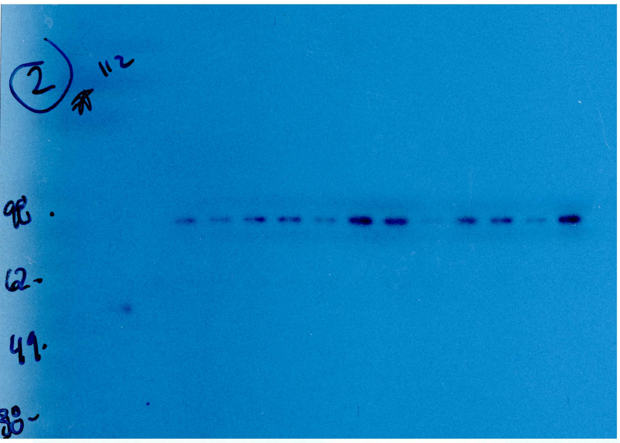
2
98
62
49

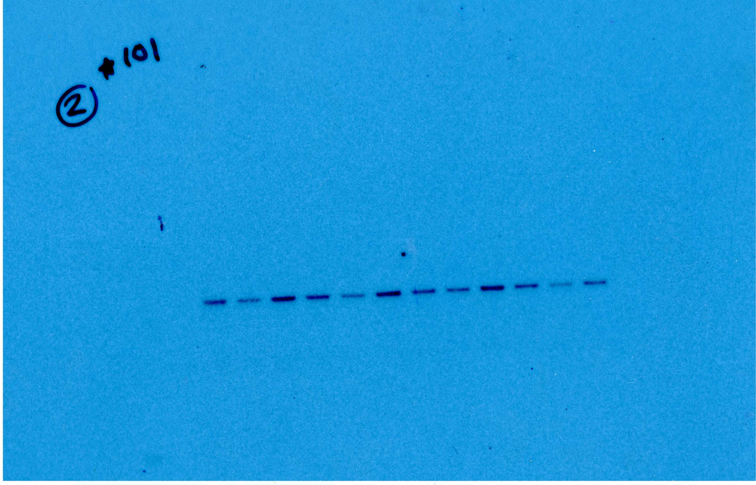
2
#101

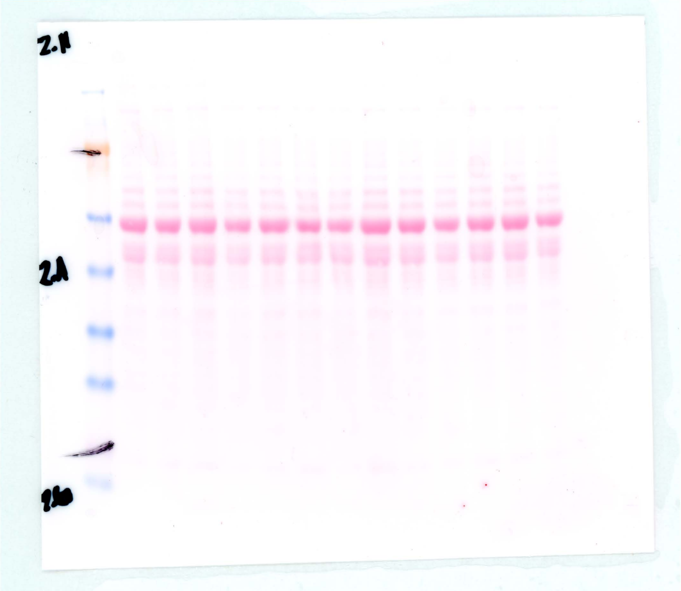
2.A

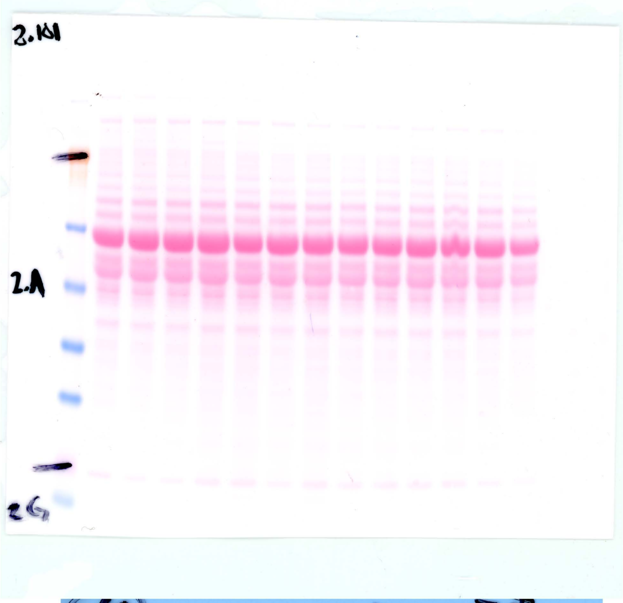
2.A

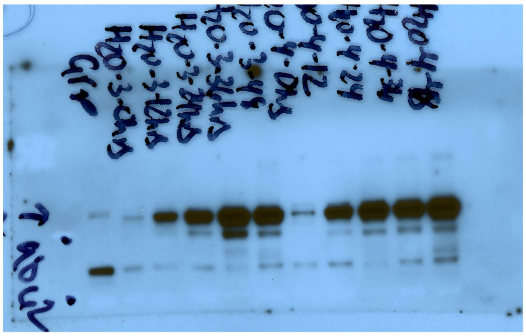

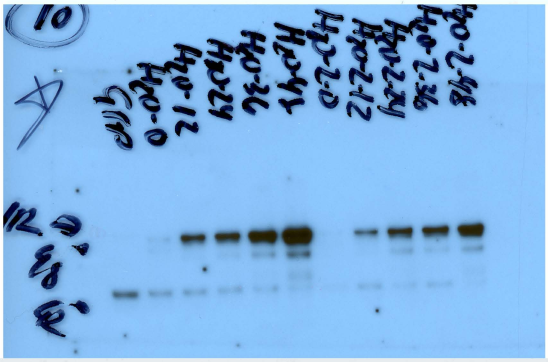
10

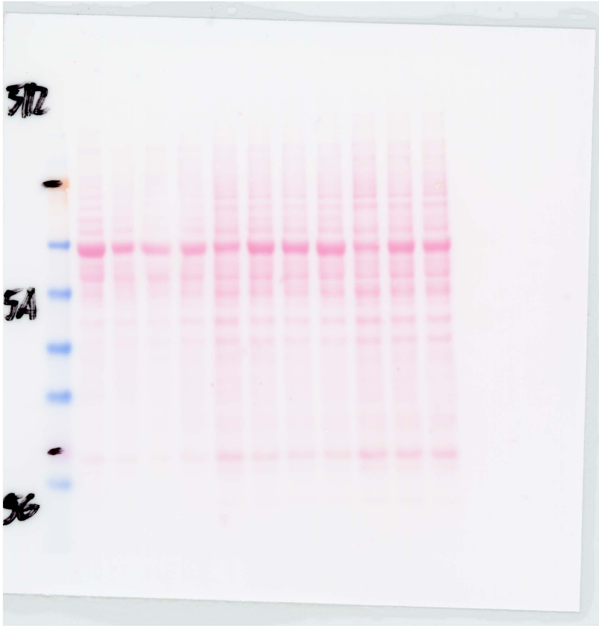
5A
5G

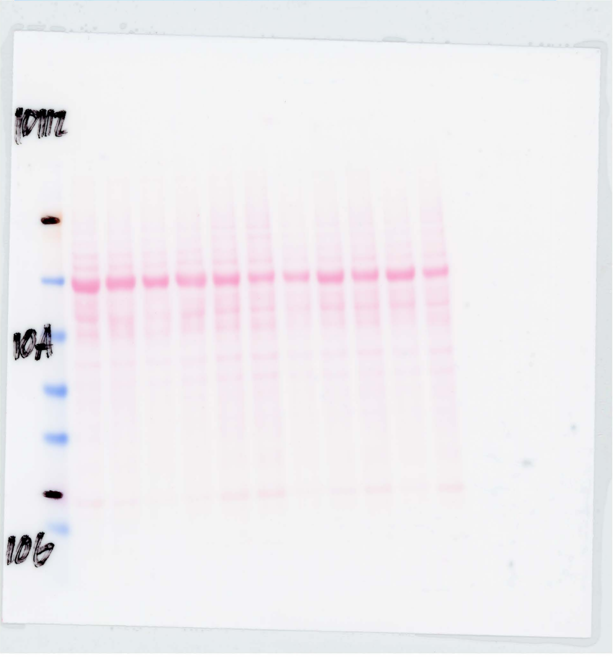
10A
10G

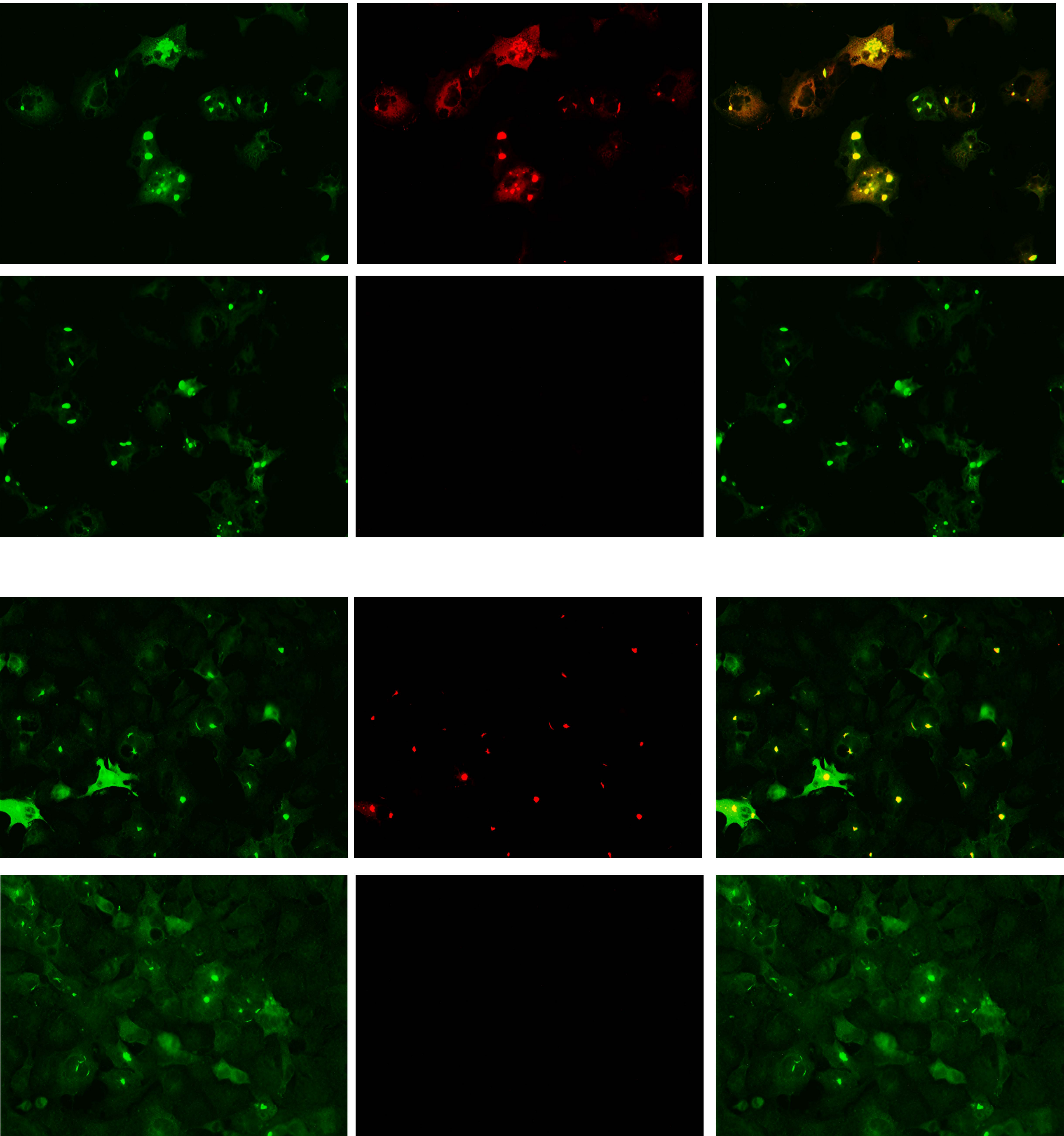

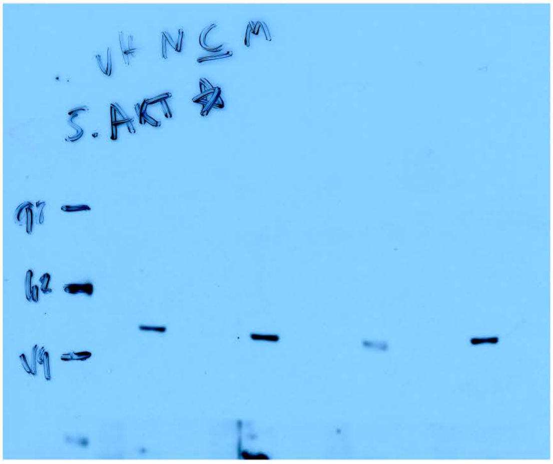
VH N C M
S. AKT
97
62
49

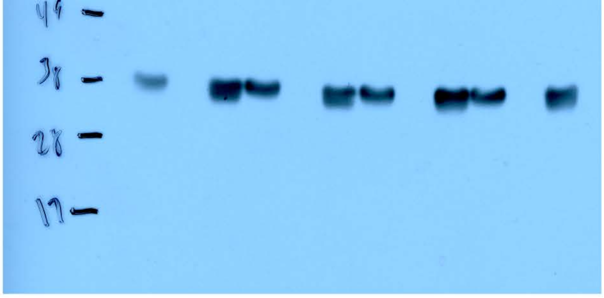
38
28
17

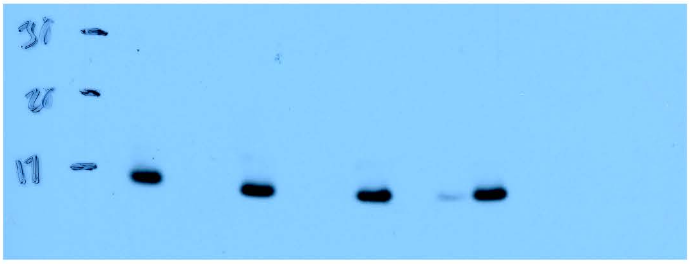
17

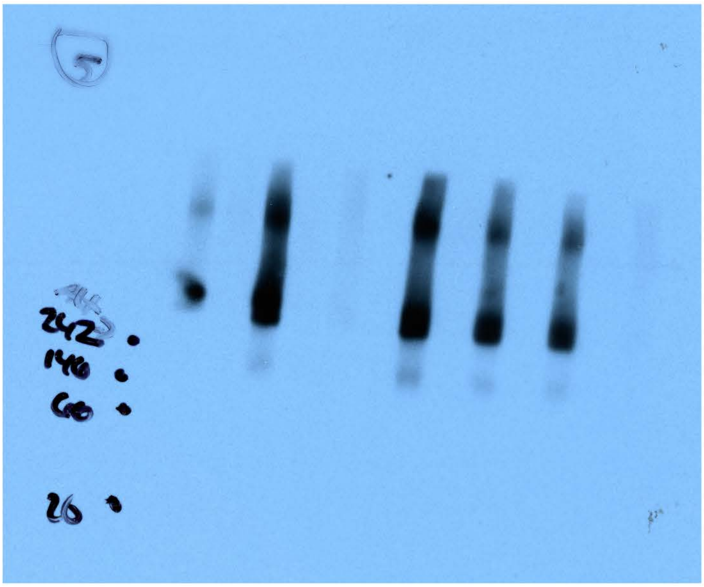
242
146
66
20

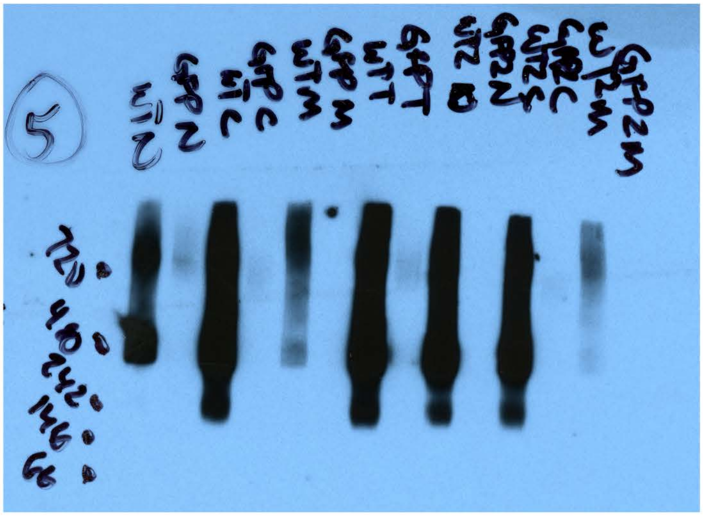
5
480
242
146
66

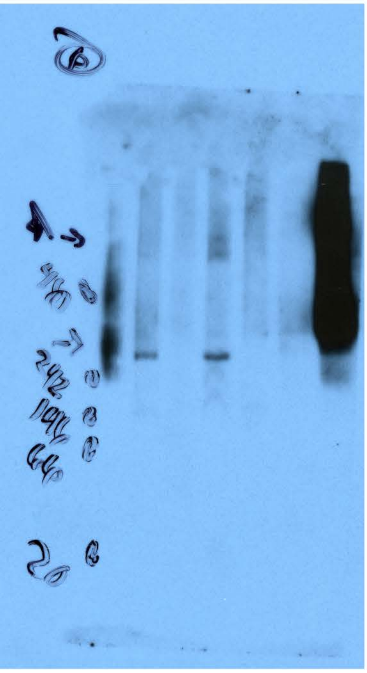
242
146
66
20

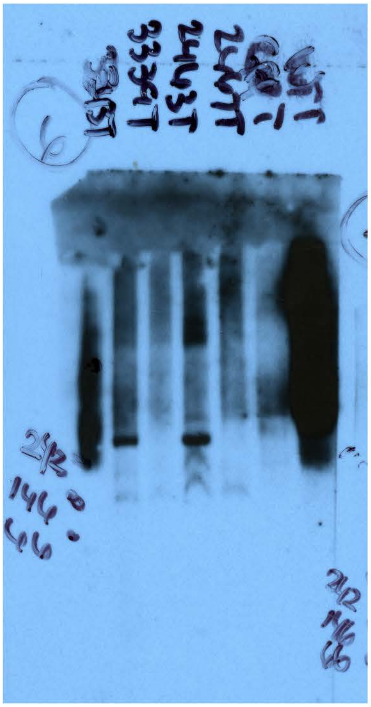
242
146
66

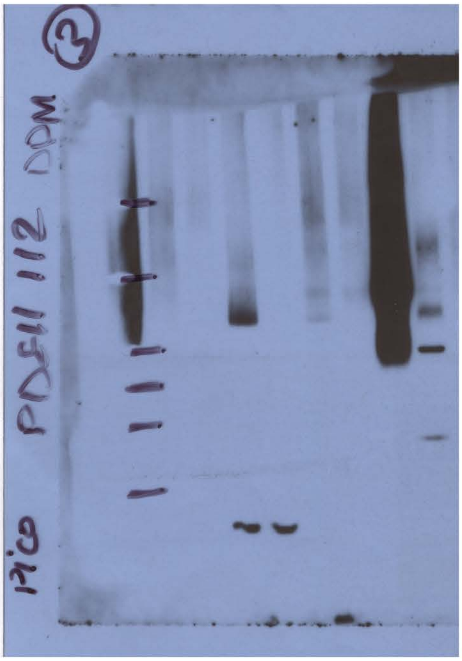
PDE11 112
DDM

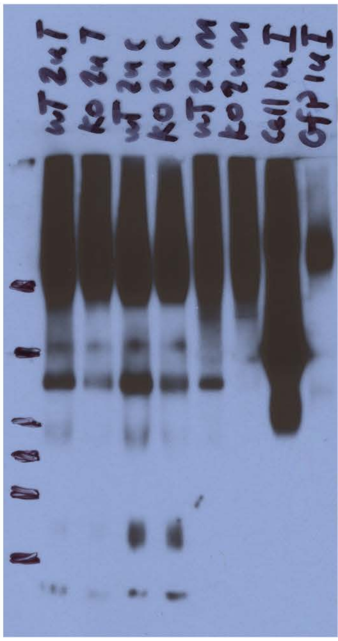

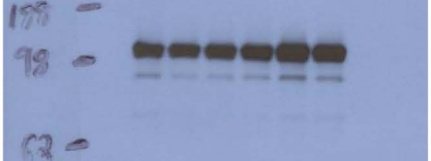
198
98
62

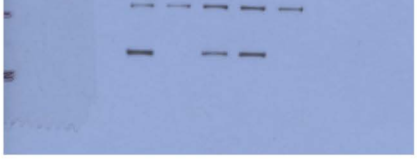

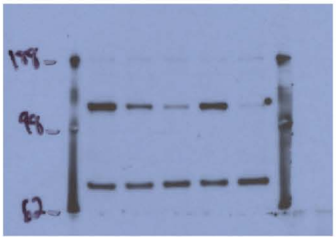
198
98
62

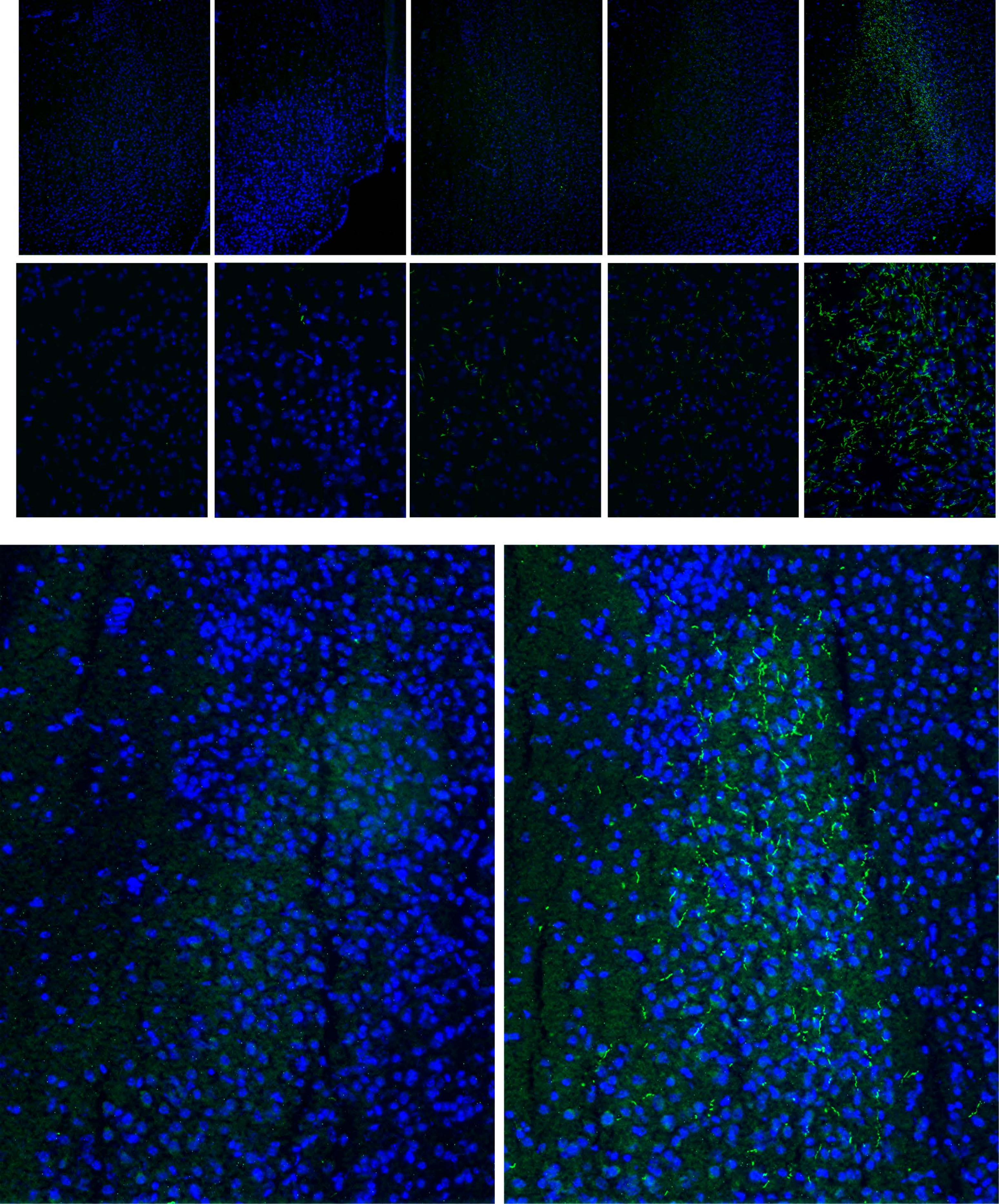

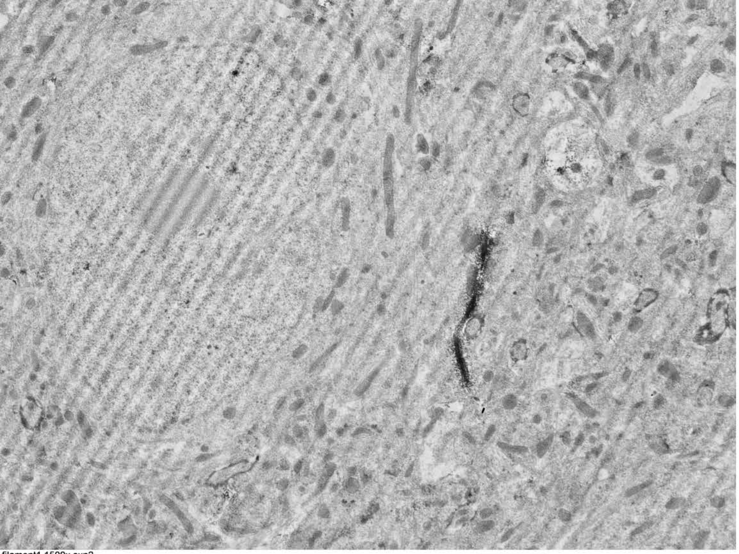

filament1 1500x sxn2
Print Mag: 8050x @ 7.0 in
14:27:07 10/3/2018

Camera: XR81, Exposure(ms): 960 Gain: 1, Bin: 1
Gamma: 1.00, No Sharpening, Normal Contrast

2 µm
HV=120.0kV
Direct Mag: 1500x
AMT Camera System

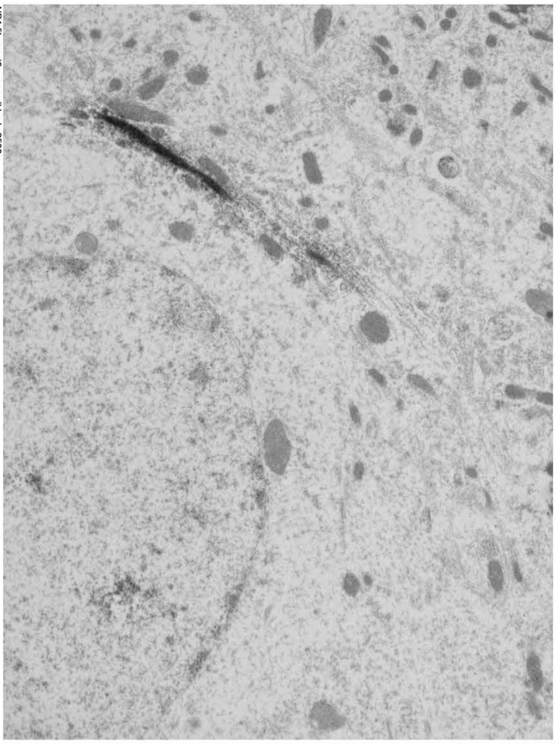

NB1 filament3 near cell body 2500x
Print Mag: 13400x @ 7.0 in
12:11:50 10/3/2018

Camera: XR81, Exposure(ms): 960 Gain: 1, Bin: 1
Gamma: 1.00, No Sharpening, Normal Contrast

1 µm
HV=120.0kV
Direct Mag: 2500x
AMT Camera System

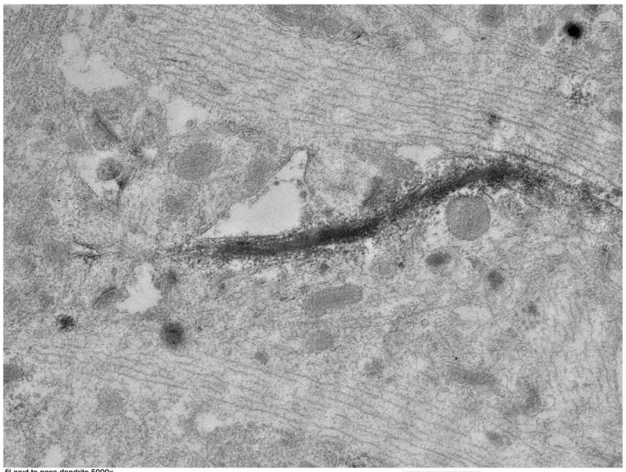

fil next to poss dendrite 5000x
Print Mag: 26800x @ 7.0 in
14:42:18 10/3/2018

Camera: XR81, Exposure(ms): 960 Gain: 1, Bin: 1
Gamma: 1.00, No Sharpening, Normal Contrast

800 nm
HV=120.0kV
Direct Mag: 5000x
AMT Camera System

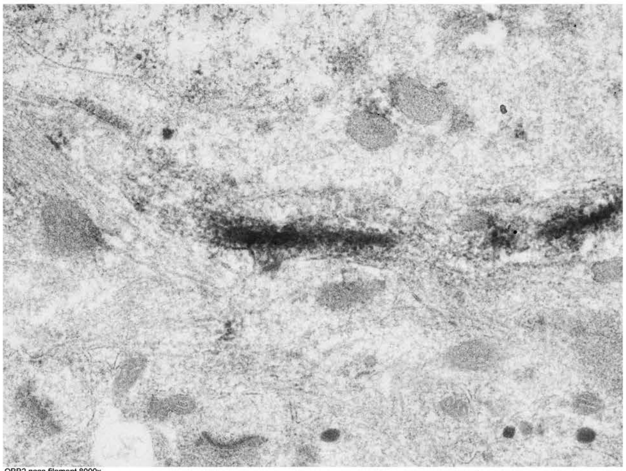

OBB2 poss filament 8000x
Print Mag: 42200x @ 7.0 in
10:39:09 8/2/2018

Camera: XR81, Exposure(ms): 960 Gain: 1, Bin: 1
Gamma: 1.00, No Sharpening, Normal Contrast

500 nm
HV=120.0kV
Direct Mag: 8000x
AMT Camera System

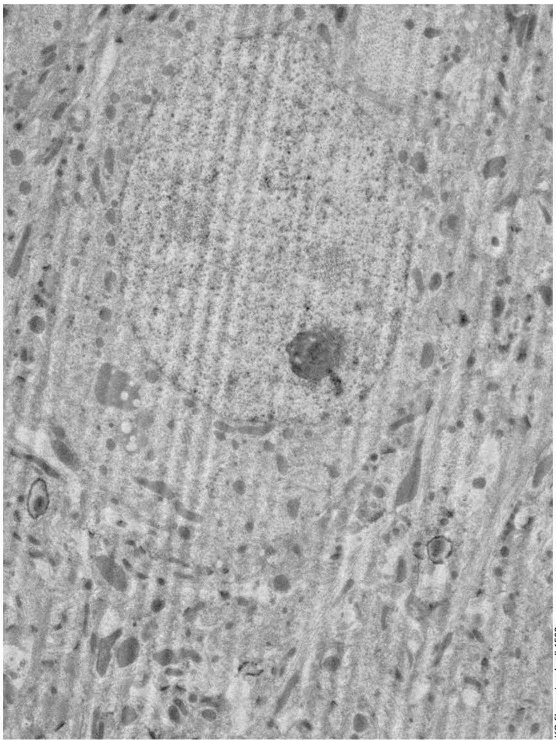

KO filament and cell 1500x
Print Mag: 8050x @ 7.0 in
13:43:40 10/3/2018

Camera: XR81, Exposure(ms): 960 Gain: 1, Bin: 1
Gamma: 1.00, No Sharpening, Normal Contrast

2 µm
HV=120.0kV
Direct Mag: 1500x
AMT Camera System

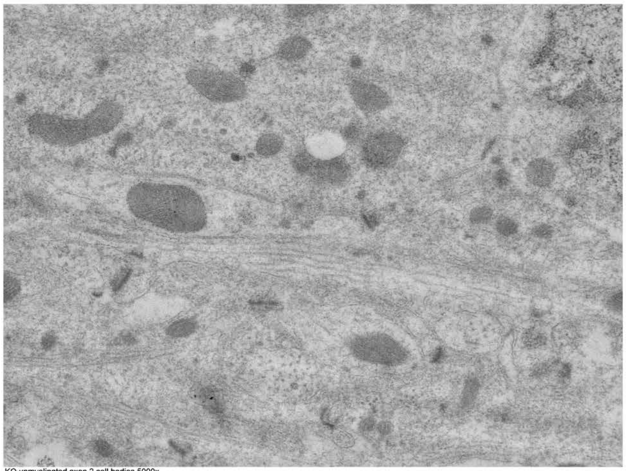

KO unmyelinated axon 2 cell bodies 5000x
Print Mag: 26800x @ 7.0 in
13:37:58 10/3/2018

Camera: XR81, Exposure(ms): 960 Gain: 1, Bin: 1
Gamma: 1.00, No Sharpening, Normal Contrast

800 nm
HV=120.0kV
Direct Mag: 5000x
AMT Camera System

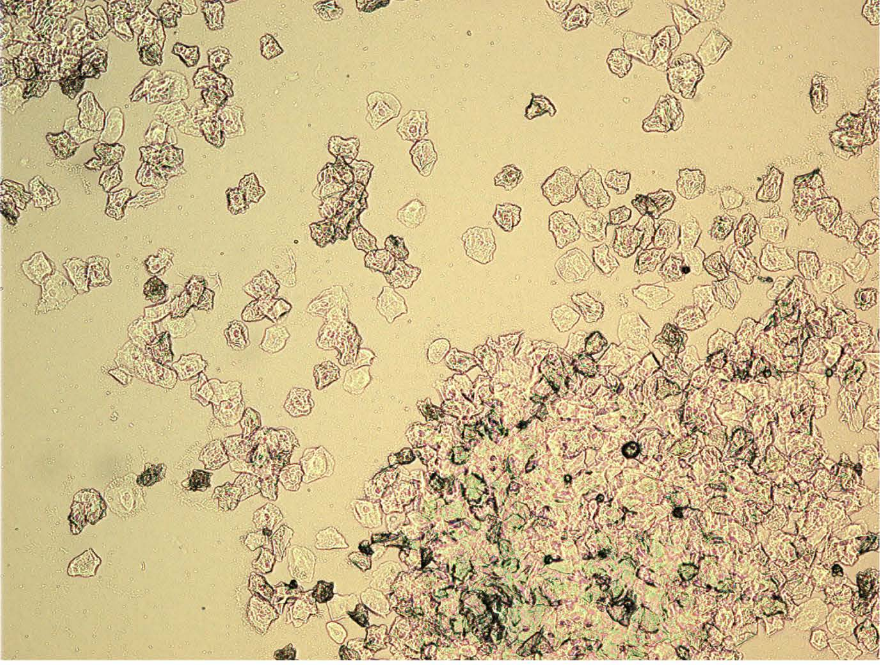

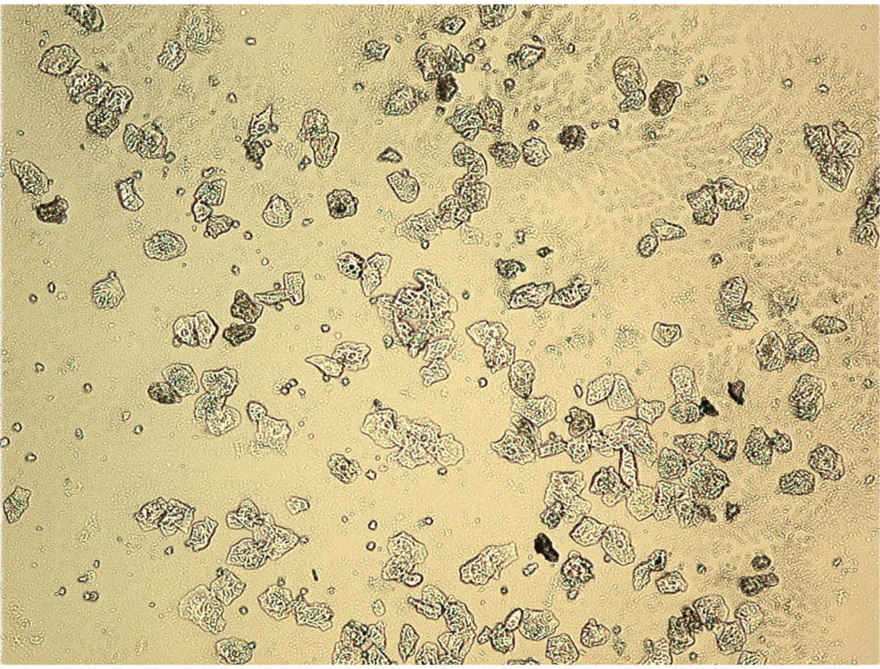

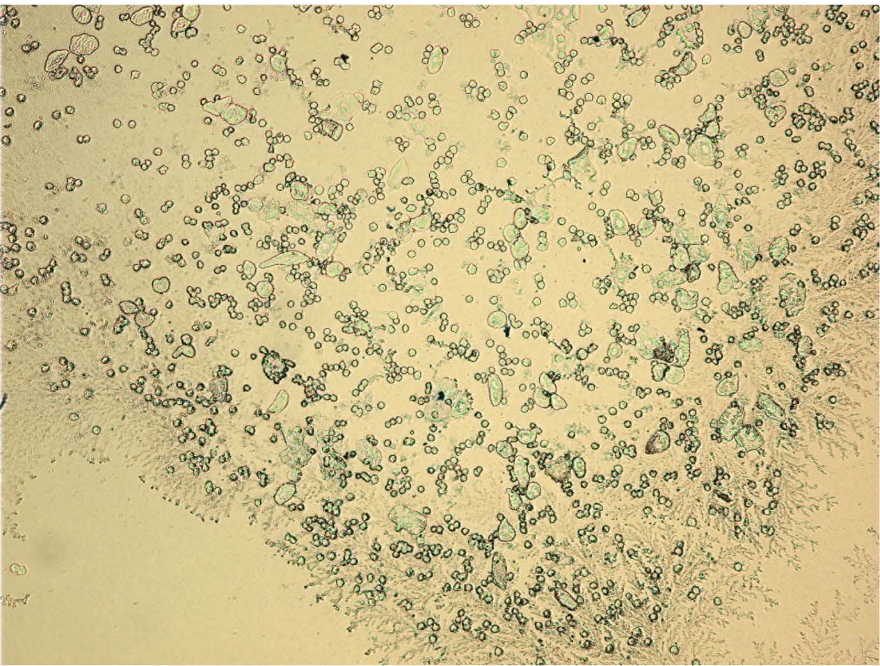

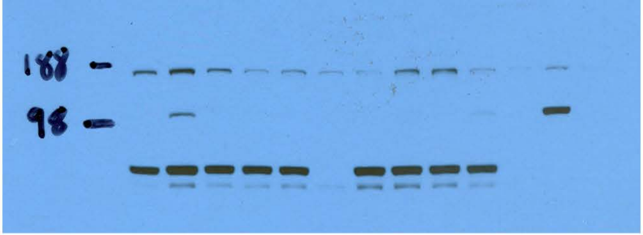
188
98

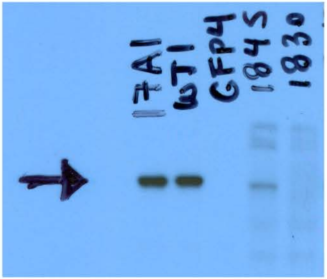
17A1
WT1
GFP4
1845
1830

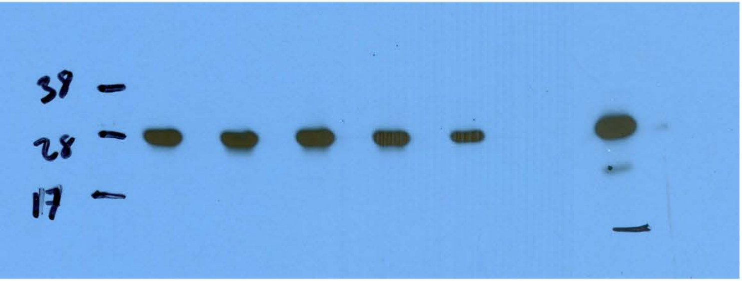
38
28
17

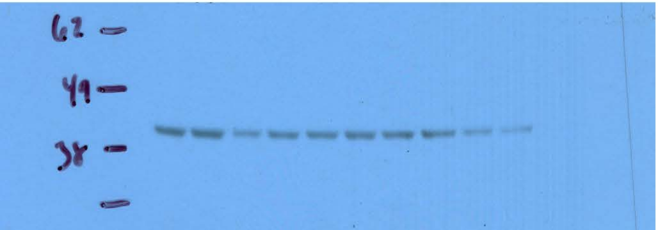
62
49
38

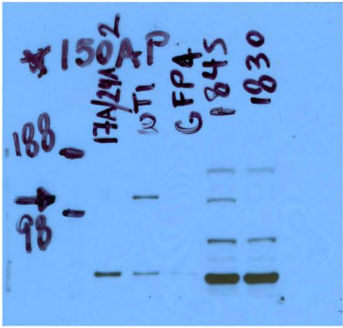
150AP
WT1
GFP4
1845
1830
188
98

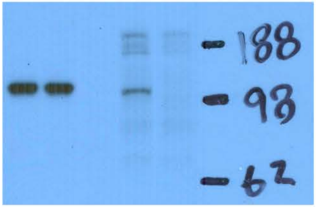
188
98
62

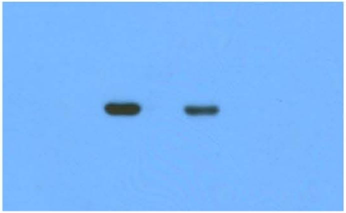

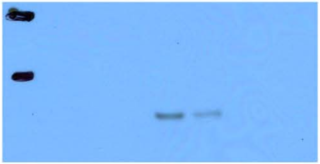

lysosome

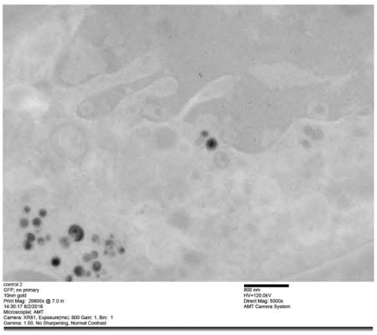
control 2
GFP; no primary
10nm gold
Print Mag: 26800x @ 7.0 in
14:30:17 6/2/2016
Microscopist: AMT
Camera: XR81, Exposure(ms): 800 Gain: 1, Bin: 1
Gamma: 1.00, No Sharpening, Normal Contrast
800 nm
HV=120.0kV
Direct Mag: 5000x
AMT Camera System

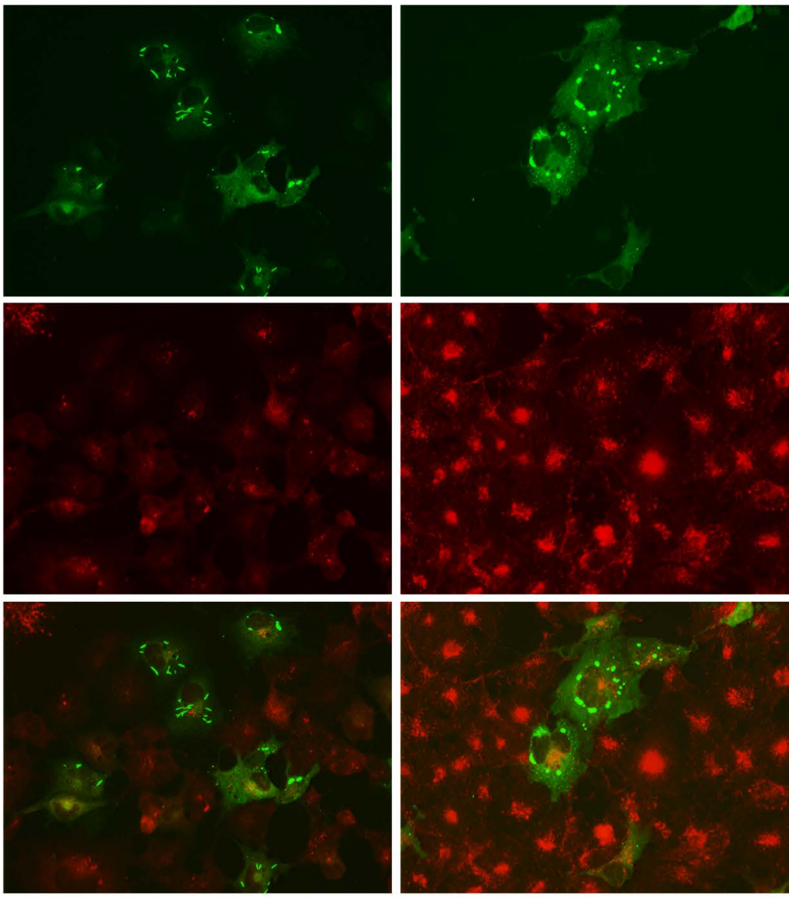

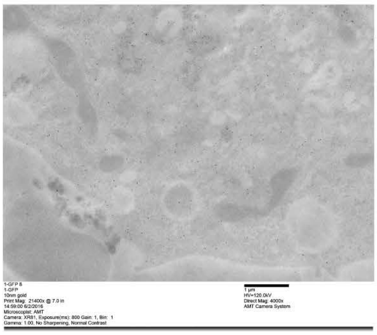
1-GFP 8
1-GFP
10nm gold
Print Mag: 21400x @ 7.0 in
14:59:00 6/2/2016
Microscopist: AMT
Camera: XR81, Exposure(ms): 800 Gain: 1, Bin: 1
Gamma: 1.00, No Sharpening, Normal Contrast
1 µm
HV=120.0kV
Direct Mag: 4000x
AMT Camera System

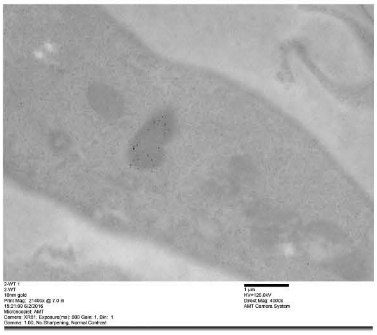
2-WT 1
2-WT
10nm gold
Print Mag: 21400x @ 7.0 in
15:21:09 6/2/2016
Microscopist: AMT
Camera: XR81, Exposure(ms): 800 Gain: 1, Bin: 1
Gamma: 1.00, No Sharpening, Normal Contrast
1 µm
HV=120.0kV
Direct Mag: 4000x
AMT Camera System

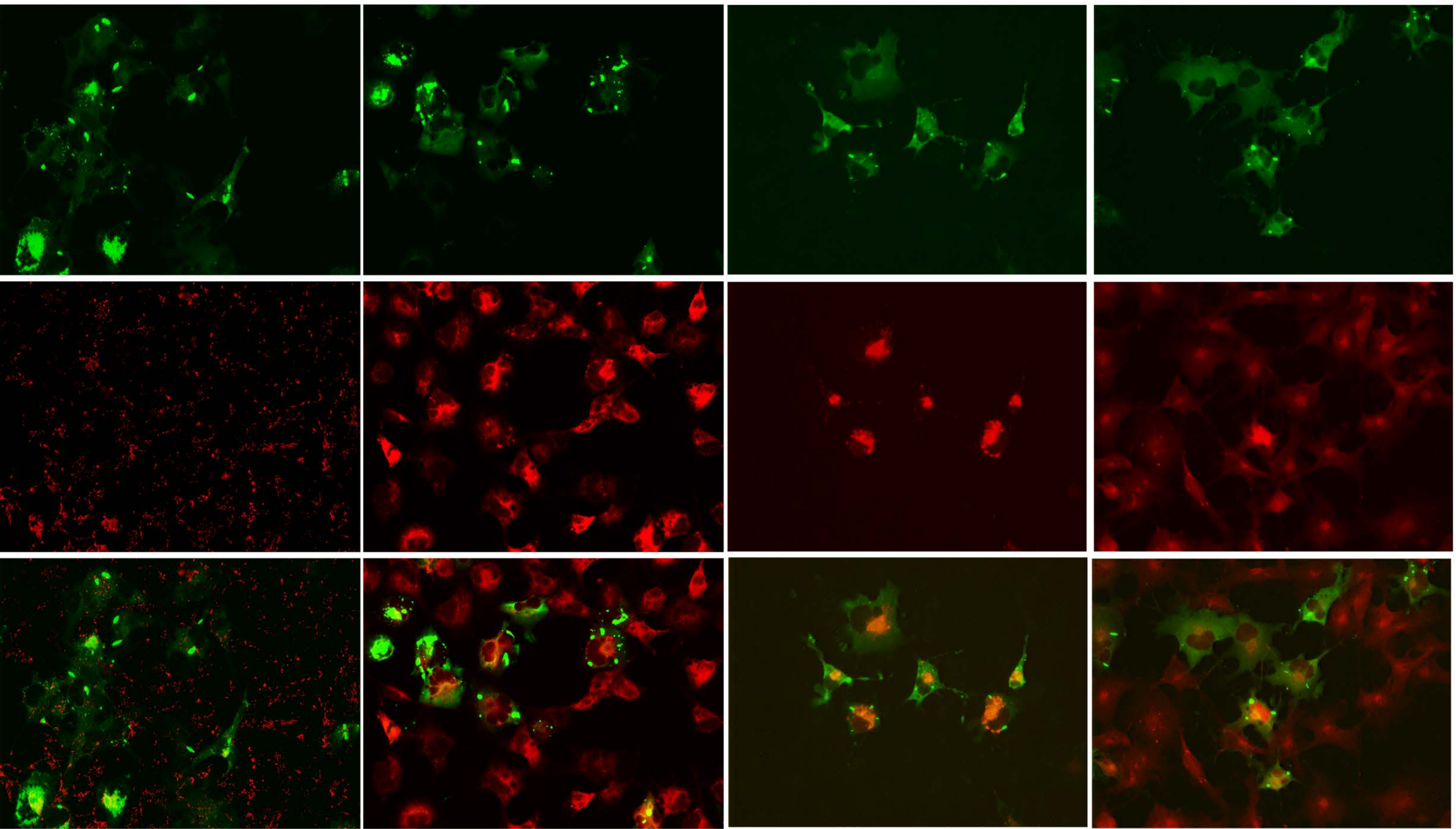

Supplement: Supplementary file 3 — Appendix S3: Uncropped images. [file ACEL-21-e13687-s005.pdf]
