# Supplementary material for: Conserved age‐related increases in hippocampal PDE11A4 cause unexpected proteinopathies and cognitive decline of social associative memories
Source: Aging Cell. 2022 Sep 8;21(10):e13687. doi: 10.1111/acel.13687 (PMC9577960; doi:10.1111/acel.13687)
Supplement: Supplementary file 6 — Table S1: Amount of food (grams) eaten during STFP tests or amount of time spent invenstigating (seconds) during SOR/NSOR tests by young (Y) and old (O) wild‐type (WT) and knockout(KO) mice during the short‐term memory (STM), 24‐hour long‐term memory (LTM), and 7‐day LTM tests (Data expressed as mean ±SEM). [file ACEL-21-e13687-s002.pdf]

Table S1. Amount of food (grams) eaten during STFP tests or amount of time spent investigating (seconds) during SOR/NSOR tests by young (Y) and old (O) wild-type (WT) and knockout (KO) mice during the short-term memory (STM), 24-hour long-term memory (LTM), and 7-day LTM tests (Data expressed as mean ±SEM).

	WT-Y	KO-Y	WT-O	KO-O	all Females	all Males	*effect of age:	*effect of sex:	*effect of genotype:
**STFP STM**	0.86 ±0.06	0.85 ±0.04	0.66 ±0.05	0.70 ±0.06	0.89 ±0.06	0.74 ±0.03	F(1,77)=7.56, P=0.007	F(1,77)=7.05, P=0.01	F(1,77)=0.33, P=0.567
**STFP 24-hr LTM**	1.3 ±0.06	1.19 ±0.07	1.10 ±0.06	1.10 ±0.05	1.22 ±0.04	1.08 ±0.05	F(1,63)=6.45, P=0.014	F(1,63)=5.09, P=0.028	F(1,63)=0.53, P=0.468
**STFP 7-d LTM**	0.86 ±0.05	0.84 ±0.05	0.71 ±0.06	0.64 ±0.05	0.82 ±0.04	0.72 ±0.04	T(49,54)=2036, P<0.001	T(45,58)=2652, P=0.038	T(50,53)=2734, P=0.376
**SOR STM**	37 ±5.78	38.5 ±4.91	28.07 ±4.63	26.8 ±3.66	39.75 ±3.46	25.94 ±3.02	T(29,30)=1006.5, P=0.039	T(28,31)=1031, P=0.004	T(29,30)=890.5, P=0.762
**SOR 24-hr LTM**	51.33 ±2.95	41.44 ±7.27	40.42 ±3.43	38.42 ±3.09	48.67 ±2.51	36.14 ±3.05	F(1,34)=4.45, P=0.043	F(1,34)=13.86, P<0.001	F(1,34)=1.74, P=0.196
**SOR 7-d LTM**	52.16 ±2.83	51.3 ±2.82	38.78 ±2.71	38.94 ±3.48	51.85 ±1.79	40.6 ±2.3	T(36,39)=956.5, P<0.001	T(33,42)=1579, P<0.001	T(37,38)=1411, P=0.962
**NSOR STM**	36.5 ±6.27	35.83 ±3.02	27.83 ±2.26	30.33 ±9.12	31.33 ±3.63	33.92 ±4.47	F(1,16)=1.29, P=0.273	F(1,16)=0.172, P=0.684	F(1,16)=0.02, P=0.885
**NSOR 24-hr LTM**	29.33 ±2.772	31 ±4.17	24.73 ±2.68	26.67 ±2.52	27.81 ±2.072	26.81 ±2.12	F(1,40)=1.99, P=0.167	F(1,40)=0.89, P=0.351	F(1,40)=0.79, P=0.379
**NSOR 7-d LTM**	12.88 ±2.52	16.56 ±2.25	10.07 ±1.8	10.68 ±1.35	12.58 ±1.31	12.29 ±1.48	T(32, 37)=1261, P=0.09	T(31,38)=1155.5, P=0.397	T(31,38)=976, P=0.19

*Data analyzed for effect of age, sex and genotype by 3-Way ANOVA (F) when data passed assumptions of normality and equal variance or by Rank Sum Test (T) when assumptions failed. Old mice ate less food in social transmission of food preference (STFP) and spent less time sniffing during social odor recognition (SOR) than young mice. Females ate more food in STFP and spent more time sniffing during SOR than males; however, there were no significant main effects of genotype nor signficant interactions with genotype. There were no effects of age, sex, or genotype on time spent sniffing during non-social odor recognition (NSOR).
