# Supplementary material for: Conserved age‐related increases in hippocampal PDE11A4 cause unexpected proteinopathies and cognitive decline of social associative memories
Source: Aging Cell. 2022 Sep 8;21(10):e13687. doi: 10.1111/acel.13687 (PMC9577960; doi:10.1111/acel.13687)
Supplement: Supplementary file 7 — Table S2: GO Processes Pathways identified as significantly enriched for both mRNA changes and phosphoproteomic changes in VHIPP old Pde11a KO vs WT [file ACEL-21-e13687-s004.pdf]

Table S2. GO Processes Pathways identified as significantly enriched for both mRNA changes and phosphoproteomic changes in VHIPP old *Pde11a* KO vs WT

			RNA sequencing pathway			Phosphoproteomics pathway		
**#term ID**	**term description**	**background gene count**	**observed gene count**	**strength**	**FDR-P=**	**observed gene count**	**strength**	**FDR-P=**
GO:0050896	response to stimulus	6616	664	0.14	7.61E-19	17	0.41	0.0034
GO:0010243	response to organonitrogen compound	867	105	0.22	3.86E-05	7	0.91	0.0052
GO:0006950	response to stress	2899	248	0.07	0.043	11	0.58	0.0072
GO:0051049	regulation of transport	1782	202	0.19	9.30E-08	9	0.7	0.0072
GO:0009987	cellular process	12459	1138	0.1	6.33E-29	21	0.23	0.0093
GO:0072347	response to anesthetic	81	14	0.38	0.0441	3	1.57	0.01
GO:0010035	response to inorganic substance	505	61	0.22	0.0035	5	1	0.0118
GO:0043279	response to alkaloid	115	18	0.33	0.0376	3	1.42	0.0182
GO:0007268	chemical synaptic transmission	321	55	0.37	2.54E-06	4	1.1	0.0183
GO:0010038	response to metal ion	344	42	0.23	0.0193	4	1.07	0.0183
GO:0032501	multicellular organismal process	5888	643	0.18	6.03E-27	14	0.38	0.0183
GO:0042493	response to drug	926	114	0.23	8.11E-06	6	0.81	0.0183
GO:0051128	regulation of cellular component organization	2337	272	0.21	7.00E-12	9	0.59	0.0183
GO:0060284	regulation of cell development	992	157	0.34	1.86E-15	6	0.78	0.0183
GO:0060359	response to ammonium ion	122	19	0.33	0.0326	3	1.39	0.0183
GO:0045664	regulation of neuron differentiation	714	117	0.35	2.71E-12	5	0.85	0.0214
GO:0007399	nervous system development	2181	334	0.33	4.19E-32	8	0.57	0.0273
GO:0051239	regulation of multicellular organismal process	2858	343	0.22	3.32E-17	9	0.5	0.03
GO:0042221	response to chemical	3532	364	0.15	7.51E-10	10	0.45	0.0309
GO:0045595	regulation of cell differentiation	1751	229	0.26	2.18E-14	7	0.6	0.0318
GO:0006816	calcium ion transport	228	32	0.29	0.0105	3	1.12	0.0367
GO:0010975	regulation of neuron projection development	547	101	0.41	3.13E-13	4	0.87	0.0448
GO:0006810	transport	3187	316	0.14	8.43E-07	9	0.45	0.0473
GO:0010033	response to organic substance	2553	267	0.16	2.00E-07	8	0.5	0.0473
GO:1901700	response to oxygen-containing compound	1429	170	0.22	9.89E-08	6	0.62	0.0473
GO:0014015	positive regulation of gliogenesis	73	15	0.45	0.0107	2	1.44	0.0496
GO:0042981	regulation of apoptotic process	1476	146	0.14	0.0034	6	0.61	0.0496
GO:0051050	positive regulation of transport	987	101	0.15	0.0098	5	0.71	0.0496

sorted by phosphoproteomic FDR-P value
