# Supplementary material for: Conserved age‐related increases in hippocampal PDE11A4 cause unexpected proteinopathies and cognitive decline of social associative memories
Source: Aging Cell. 2022 Sep 8;21(10):e13687. doi: 10.1111/acel.13687 (PMC9577960; doi:10.1111/acel.13687)
Supplement: Supplementary file 8 — Table S3: GO Component Pathways identified as significantly enriched for both mRNA changes and phosphoproteomic changes in VHIPP old Pde11a KO vs WT mice. [file ACEL-21-e13687-s008.pdf]

Table S3. GO Component Pathways identified as significantly enriched for both mRNA changes and phosphoproteomic changes in VHIPP old *Pde11a* KO vs WT mice.

			RNA sequencing pathway			Phosphoproteomics pathway		
**#term ID**	**term description**	**background gene count**	**observed gene count**	**strength**	**FDR-P=**	**observed gene count**	**strength**	**FDR-P=**
GO:0120025	plasma membrane bounded cell projection	2172	273	0.24	1.22E-15	12	0.74	2.11E-05
GO:0043005	neuron projection	1429	207	0.3	4.05E-17	10	0.85	2.11E-05
GO:0005737	cytoplasm	9909	858	0.08	3.08E-10	21	0.33	2.54E-05
GO:0008021	synaptic vesicle	183	24	0.26	0.0465	5	1.44	3.27E-05
GO:0045202	synapse	968	150	0.33	2.44E-14	8	0.92	6.43E-05
GO:0005622	intracellular	12462	1099	0.09	6.33E-20	22	0.25	7.08E-05
GO:0030425	dendrite	694	117	0.37	2.56E-13	7	1	7.08E-05
GO:0098793	presynapse	429	60	0.29	9.86E-05	6	1.15	7.08E-05
GO:0032991	protein-containing complex	4701	479	0.15	2.56E-13	14	0.47	0.00023
GO:0005829	cytosol	3326	283	0.07	0.0249	12	0.56	0.00023
GO:0098796	membrane protein complex	1009	119	0.21	1.56E-05	7	0.84	0.00035
GO:0005623	cell	14044	1233	0.08	1.32E-26	22	0.2	0.00041
GO:0030424	axon	712	123	0.38	1.48E-14	6	0.93	0.00044
GO:0043232	intracellular non-membrane-bounded organelle	3809	352	0.11	3.01E-05	12	0.5	0.00062
GO:0099512	supramolecular fiber	809	96	0.21	0.00012	6	0.87	0.00079
GO:0030018	Z disc	127	23	0.4	0.0022	3	1.37	0.0016
GO:0043226	organelle	10897	933	0.07	9.82E-11	19	0.24	0.0019
GO:0042383	sarcolemma	147	31	0.46	2.11E-05	3	1.31	0.0022
GO:0044297	cell body	776	111	0.3	6.54E-09	5	0.81	0.0043
GO:1902494	catalytic complex	1266	131	0.15	0.00094	6	0.68	0.0046
GO:0043229	intracellular organelle	10645	907	0.07	1.19E-09	18	0.23	0.0049
GO:0005886	plasma membrane	4328	480	0.19	1.98E-19	11	0.41	0.005
GO:0031982	vesicle	1837	185	0.14	0.00016	7	0.58	0.0054
GO:0031410	cytoplasmic vesicle	1710	173	0.15	0.00023	6	0.55	0.016
GO:0043227	membrane-bounded organelle	9775	822	0.06	5.17E-07	16	0.21	0.0182
GO:0005911	cell-cell junction	417	53	0.24	0.0021	3	0.86	0.0214
GO:0043231	intracellular membrane-bounded organelle	9088	751	0.06	6.42E-05	15	0.22	0.0257
GO:0043204	perikaryon	170	25	0.31	0.0138	2	1.07	0.0318
